# Correction: Amelioration of non-alcoholic fatty liver disease by targeting adhesion G protein-coupled receptor F1 (Adgrf1)

**DOI:** 10.7554/eLife.96183

**Published:** 2024-01-22

**Authors:** Mengyao Wu, Tak-Ho Lo, Liping Li, Jia Sun, Chujun Deng, Ka-Ying Chan, Xiang Li, Steve Ting-Yuan Yeh, Jimmy Tsz Hang Lee, Pauline Po Yee Lui, Aimin Xu, Chi-Ming Wong

**Keywords:** Human

Wu M, Lo T-H, Li L, Sun J, Deng C, Chan K-Y, Li X, Yeh ST-Y, Lee JTH, Lui PPY, Xu A, Wong C-M. 2023. Amelioration of non-alcoholic fatty liver disease by targeting adhesion G protein-coupled receptor F1 (Adgrf1). eLife **12**:e85131. doi: 10.7554/eLife.85131.

Published 15 August 2023

We were informed through PubPeer about errors in several figures in this paper. Specifically, they referred to a duplication of the blot of β-actin in Figure 1D and Figure 2—figure supplement 1C, and overlapping images among three Oil Red O panels and two of the Masson panels in Figure 4G and Figure 8G. In addition, upon careful reanalysis of all data, we noticed a duplication of the blot of β-Tubulin for Figure 6B and Figure 3C, and an error in Figure 2—figure supplement 1C that were not mentioned by PubPeer. We now provided explanation and corrective action for each of these errors.

We have made the following corrections:

There is a duplication of the blot of β-actin in Figure 1D and Figure 2—figure supplement 1C. The β-actin blot in Figure 1D was found to be incorrect upon investigation. This error was due to the use of Figure 2—figure supplement 1C as the template for generating Figure 1D in order to make the same font size and image dimensions. During the process, the image of the loading control β-actin for the liver in Figure 2—figure supplement 1C was inadvertently left partially unreplaced, resulting in the duplication of images. The blot of β-actin in Figure 2—figure supplement 1C is correct. We revised Figure 1D with the correct image of the loading control β-actin. The quantification of band intensities in Figure 1D was based on the correct image of the loading control β-actin in the original published figure, therefore the bar chart on the right remains valid. All the statistics, the other panels and the caption of Figure 1D are correct. The changes do not affect the results or conclusions of the paper. The source data has been updated accordingly.

The corrected Figure 1 is shown here:

**Figure 1:**
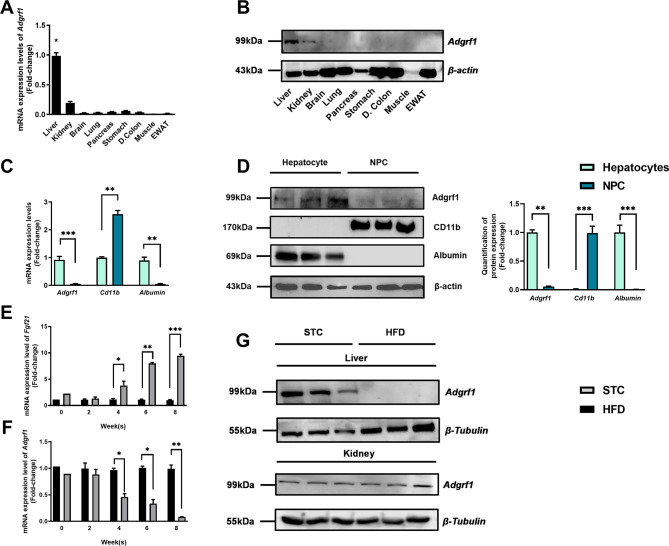


The originally published Figure 1 is shown for reference:

**Figure 2:**
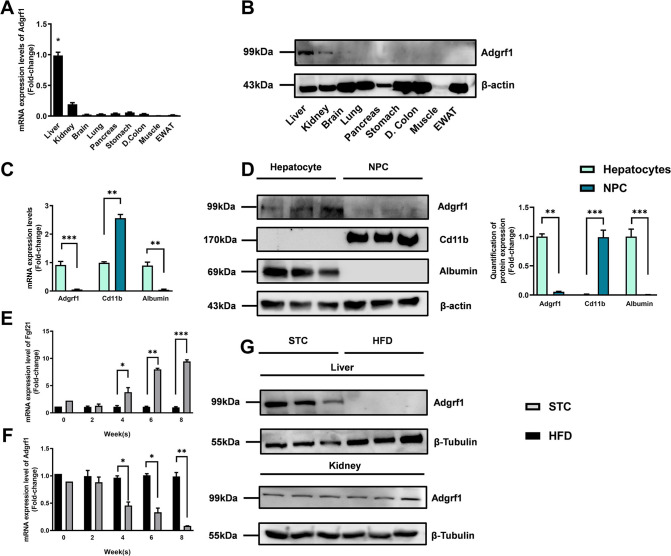


There is a duplication of the blot of β-Tubulin in Figure 6B and Figure 3C. The β-Tubulin blot in Figure 6B was found to be incorrect upon investigation. This error was due to the use of Figure 3C as the template to generate Figure 6B in order to make the same font size and image dimensions. During the process, the image of the loading control β-Tubulin used to demonstrate equal loading in Figure 3C was inadvertently left partially unreplaced, resulting in the duplication of images. The blot of β-Tubulin in Figure 3C is correct. We revised Figure 6B with the correct image of the loading control β-Tubulin. We would like to highlight that the quantification of band intensities in Figure 6B was based on the correct image of the loading control β-Tubulin in the original published figure, therefore the bar chart for quantification on the right remains valid. All the statistics, the other panels and the caption of Figure 6 are correct. The changes do not affect the results or conclusions of the paper. The source data has been updated accordingly.

The corrected Figure 6 is shown here:

**Figure 3:**
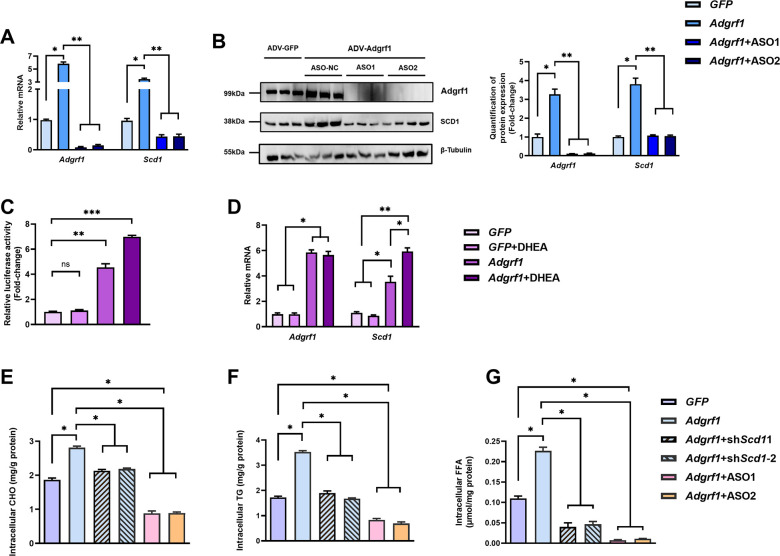


The originally published Figure 6 is shown for reference:

**Figure 4:**
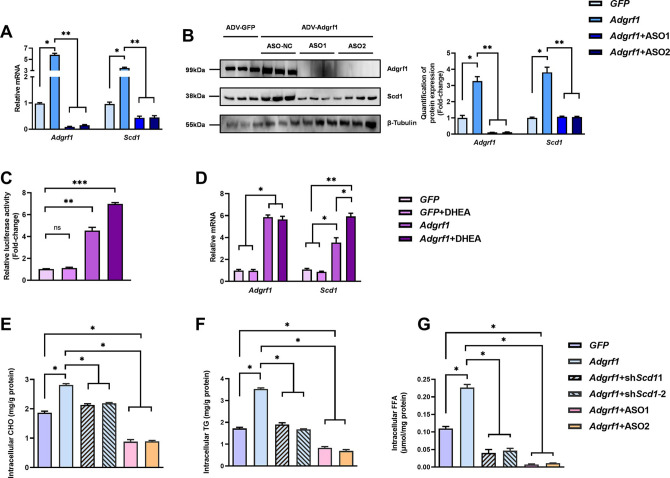


There has been a copy and paste error identified in the image of the loading control β-actin for the kidney in Figure 2—figure supplement 1C. Upon investigation, it was determined that the copied image was incorrect. We revised Figure 2—figure supplement 1 with the correct image of the loading control β-actin for kidney. We re-quantified the band intensity with the correct image of the loading control. There was no significant difference in the expression of Adgrf1 in the kidneys and the conclusion remained unchanged. All the statistics, the other panels and the caption of Figure 2—figure supplement 1 are correct. The changes do not affect the results or conclusions of the paper. The source data has been updated accordingly.

The corrected Figure 2—figure supplement 1 is shown here:

**Figure 5:**
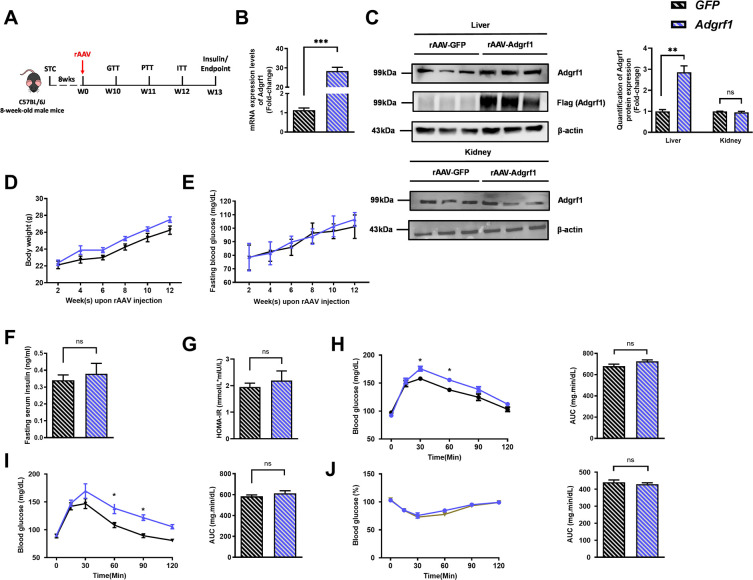


The originally published Figure 2—figure supplement 1 is shown for reference:

**Figure 6:**
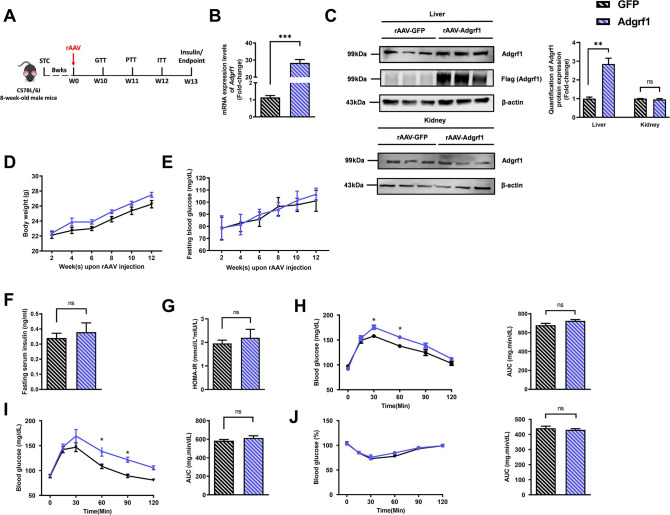


There is an overlap of three Oil Red O staining images for rAAV-GFP treated with vehicle and rAAV-Adgrf1 treated with MK8245 in Figure 8G with the images for rAAV-Adgrf1 treated with ASO2-Adgrf1 in Figure 4G. The Oil Red O staining images in Figure 8G were found to be incorrect upon investigation. This error was due to the large number of Oil Red O staining images captured and the existence of different microscopic views obtained from a single slide, which were saved with numerical codes in the same folder. There were some inadvertent image mix-ups during the process of assembling Figure 8G. The Oil Red O staining image for rAAV-Adgrf1 treated with ASO2-Adgrf1 in Figure 4G are correct. We revised Figure 8G with correct Oil Red O images. The changes do not affect the results or conclusions of the paper. In addition, we have included the original Oil Red O staining images for rAAV-GFP treated with vehicle and rAAV-Adgrf1 treated with MK8245 in the published article as the source data.

There is a concern raised on PubPeer regarding the potential overlap between two Masson panels in Figure 4G and 8G for the control group HFD rAAV-GFP mice. It has been noted that one panel appears stretched relative to the other. We would like to clarify that that both Masson staining panels in question are indeed individual images obtained from the same sample slide but with overlapping views. They represent the control group of HFD rAAV-GFP mice treated with the vehicle under identical experimental conditions. In this case, using a sample image is acceptable as it accurately represents the experimental findings. To prevent any potential confusion among readers, we have taken this opportunity to replace the Masson staining image for the control group in Figure 8G. The revised figure ensures clarity for all readers. The changes do not affect the results or conclusions of the paper. We apologize for the confusion that this may have caused. The source data has been updated accordingly.

The corrected Figure 8 is shown here:

**Figure 7:**
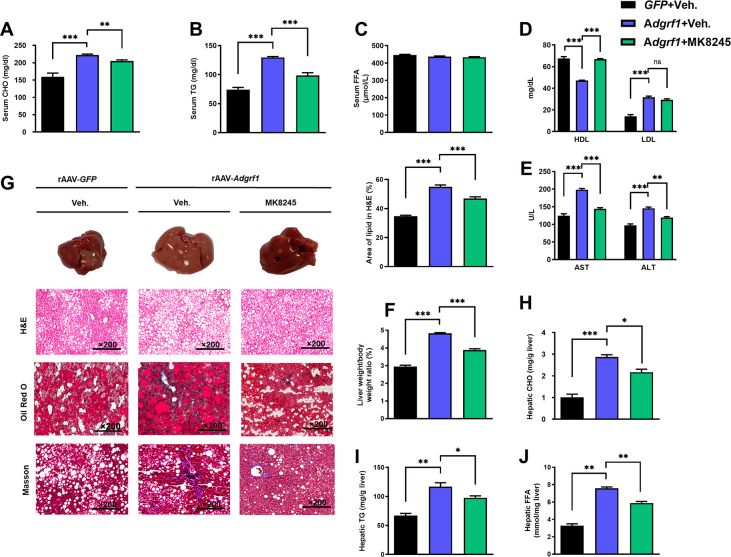


The originally published Figure 8 is shown for reference:

**Figure 8:**
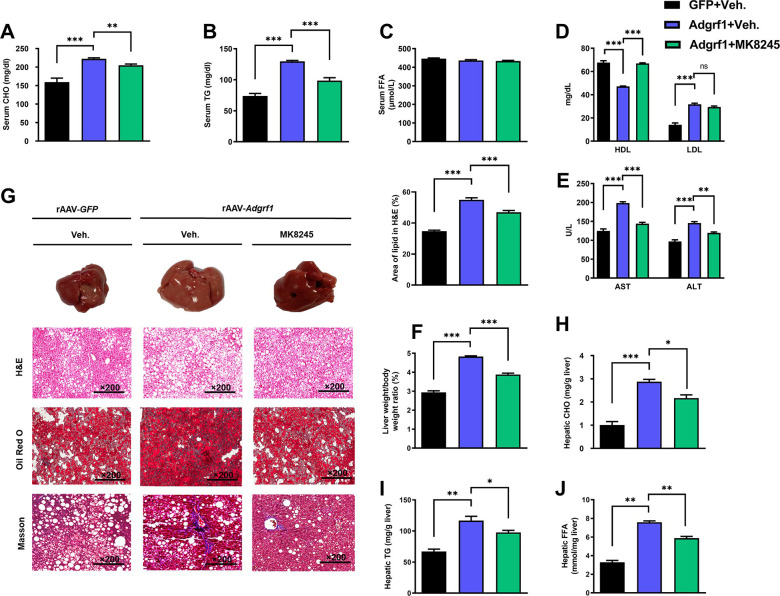


Finally, source data for all figures has been updated to include all individual original gel blot images. The article has been corrected accordingly.

