# Supplementary material for: Amelioration of non-alcoholic fatty liver disease by targeting adhesion G protein-coupled receptor F1 (Adgrf1)
Source: eLife. 2023 Aug 15;12:e85131. doi: 10.7554/eLife.85131 (PMC10427146; doi:10.7554/eLife.85131)
Supplement: Figure 1—source data 1. [file elife-85131-fig1-data1.zip › Figure 1-source data 1/Figure 1-Source 2.pptx]

## Slide 1
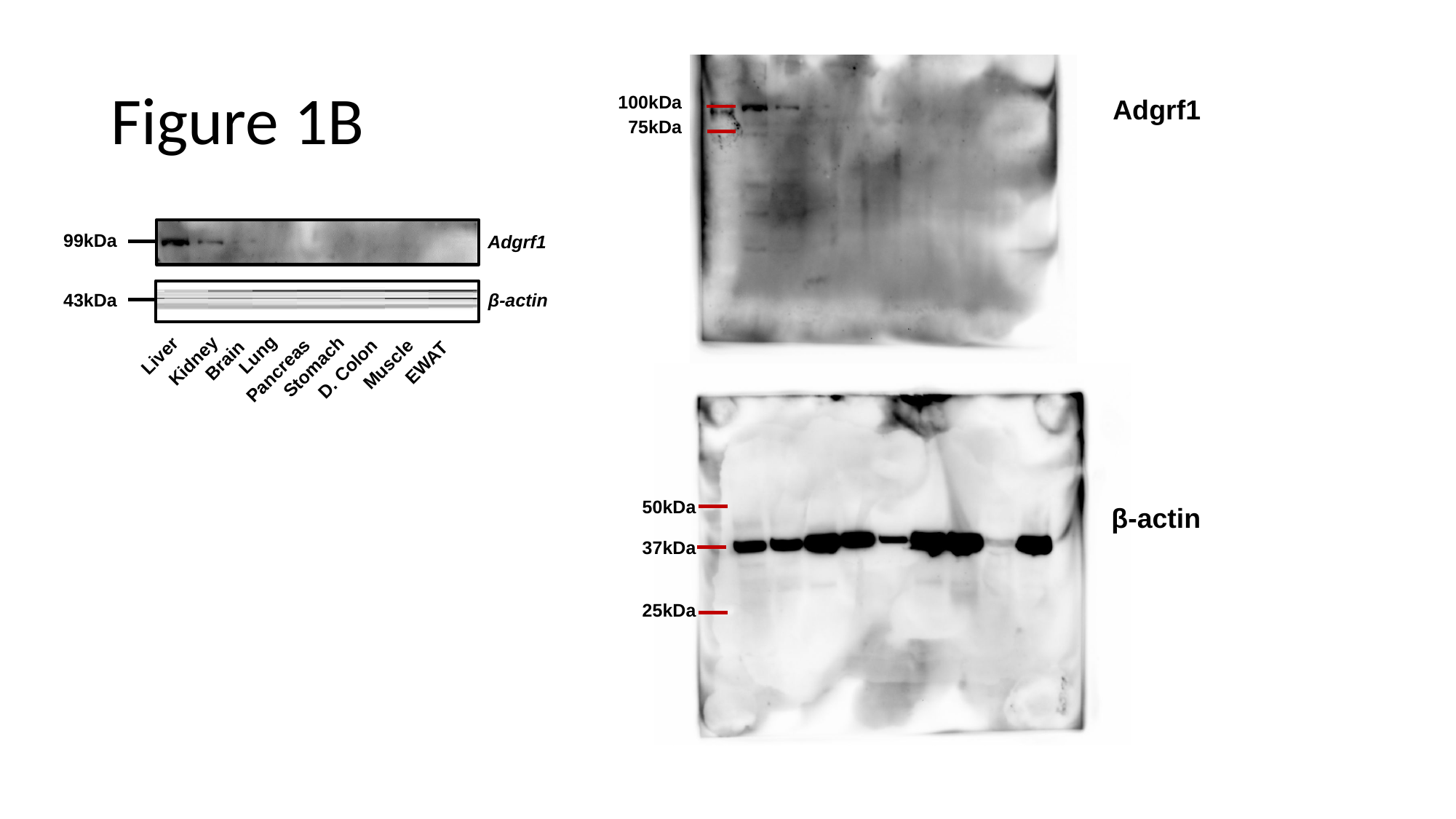

# Figure 1B
100kDa
75kDa
Adgrf1
99kDa
Adgrf1
43kDa
β-actin
Lung
Liver
Kidney
EWAT
Muscle
Stomach
D. Colon
Pancreas
Brain
50kDa
37kDa
25kDa
β-actin

## Slide 2
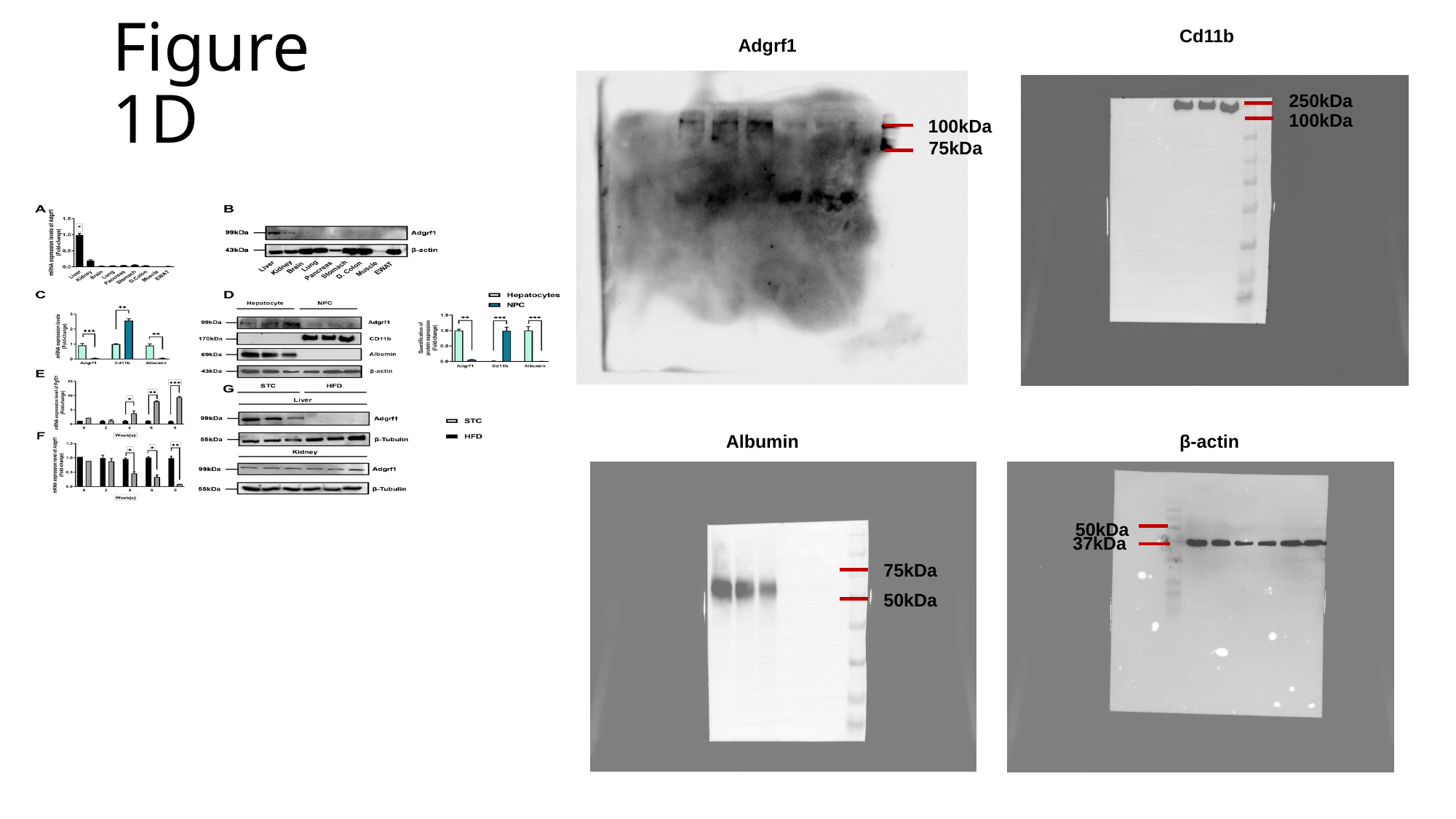

Figure 1D
Cd11b
Adgrf1
250kDa
100kDa
100kDa
 75kDa
Albumin
β-actin
50kDa
 37kDa
75kDa
50kDa

## Slide 3
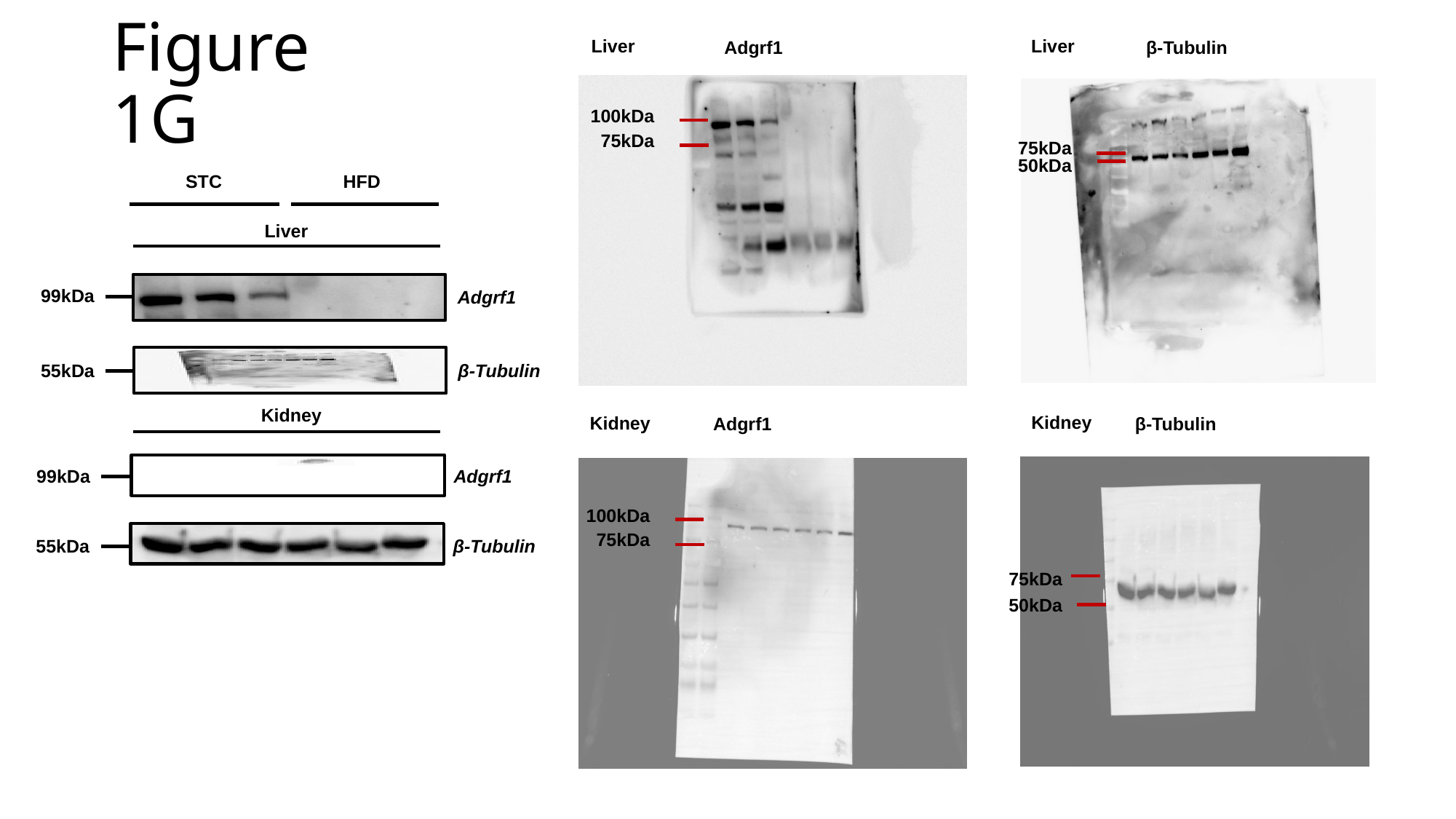

Figure 1G
Liver
Liver
Adgrf1
β-Tubulin
100kDa
75kDa
75kDa
50kDa
STC
HFD
Liver
99kDa
Adgrf1
55kDa
β-Tubulin
Kidney
99kDa
Adgrf1
55kDa
β-Tubulin
Kidney
Kidney
Adgrf1
β-Tubulin
100kDa
75kDa
75kDa
50kDa
