# Supplementary figures and images for: Amelioration of non-alcoholic fatty liver disease by targeting adhesion G protein-coupled receptor F1 (Adgrf1)
Source: eLife. 2023 Aug 15;12:e85131. doi: 10.7554/eLife.85131 (PMC10427146; doi:10.7554/eLife.85131)

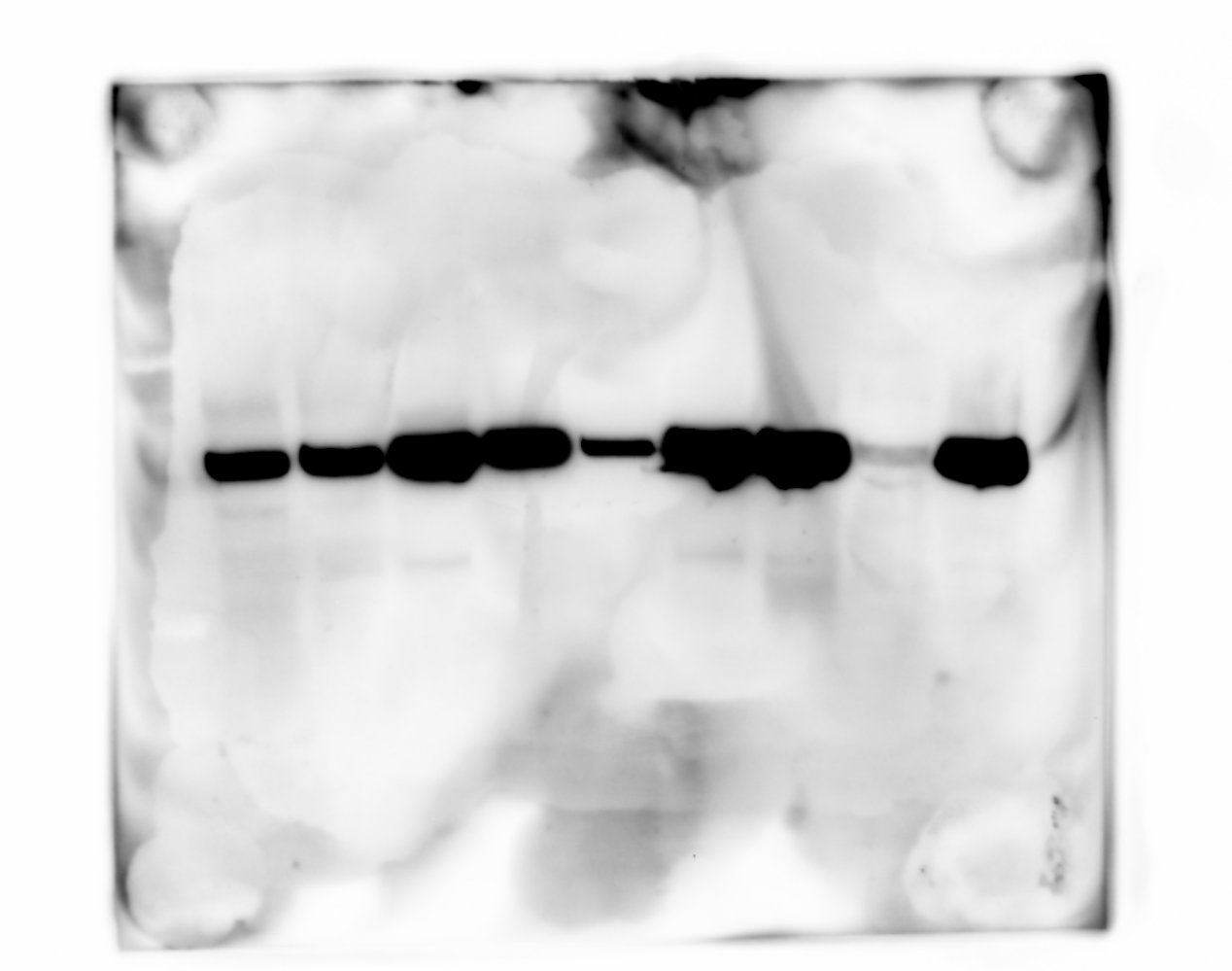

Supplement: Figure 1—source data 1. [file elife-85131-fig1-data1.zip › Figure 1-source data 1/Figure 1-raw gel image/Fig1B b-actin.tif]

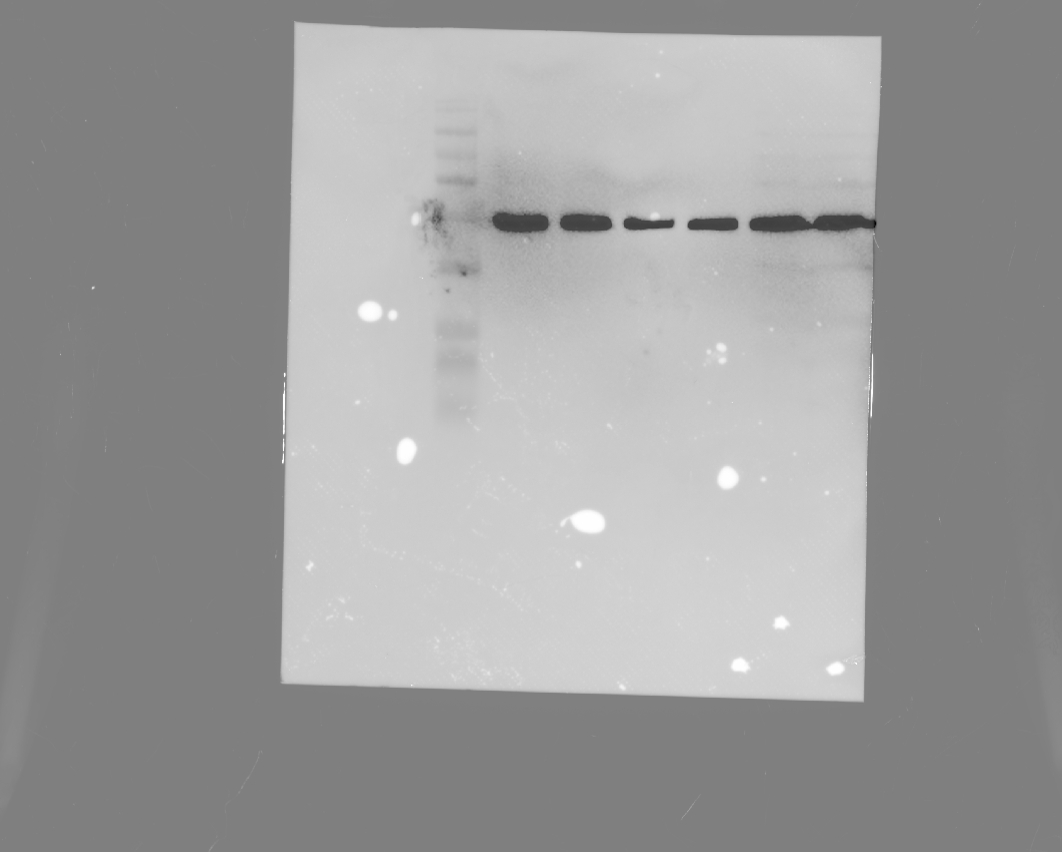

Supplement: Figure 1—source data 1. [file elife-85131-fig1-data1.zip › Figure 1-source data 1/Figure 1-raw gel image/Fig1D b-actin.tif]

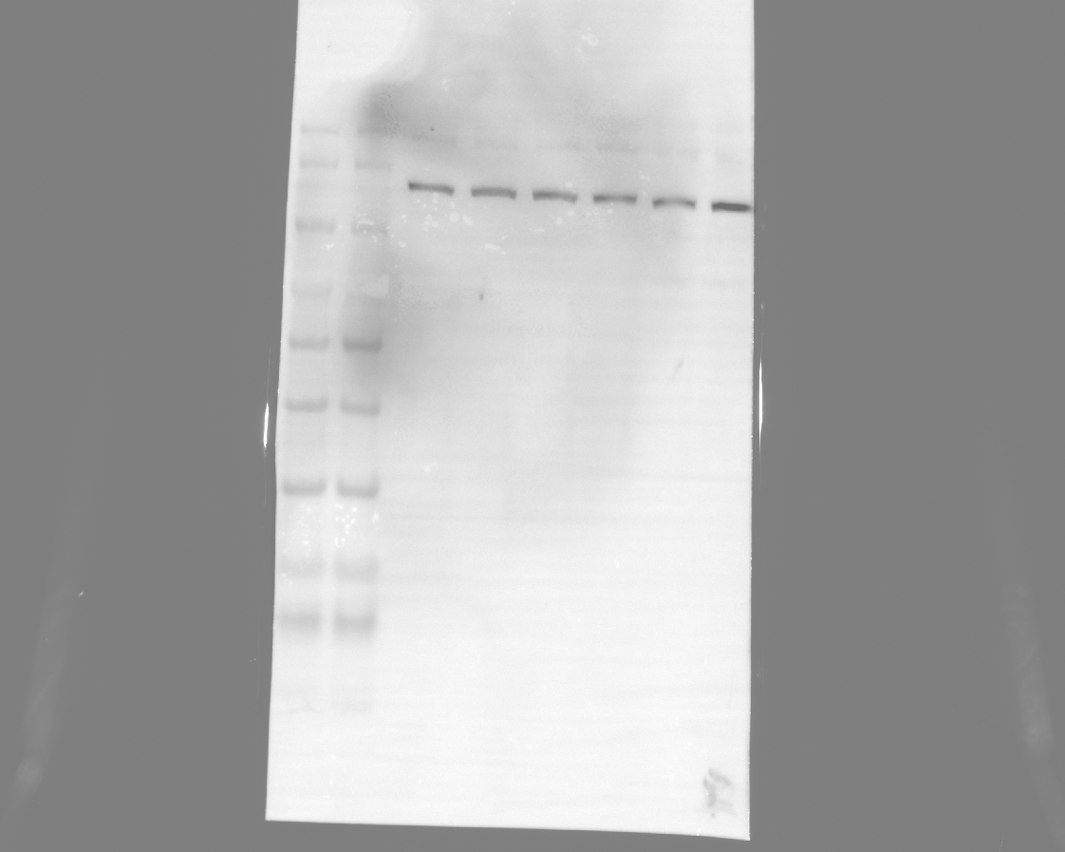

Supplement: Figure 1—source data 1. [file elife-85131-fig1-data1.zip › Figure 1-source data 1/Figure 1-raw gel image/Fig1G Adgrf1 (kidney).tif]

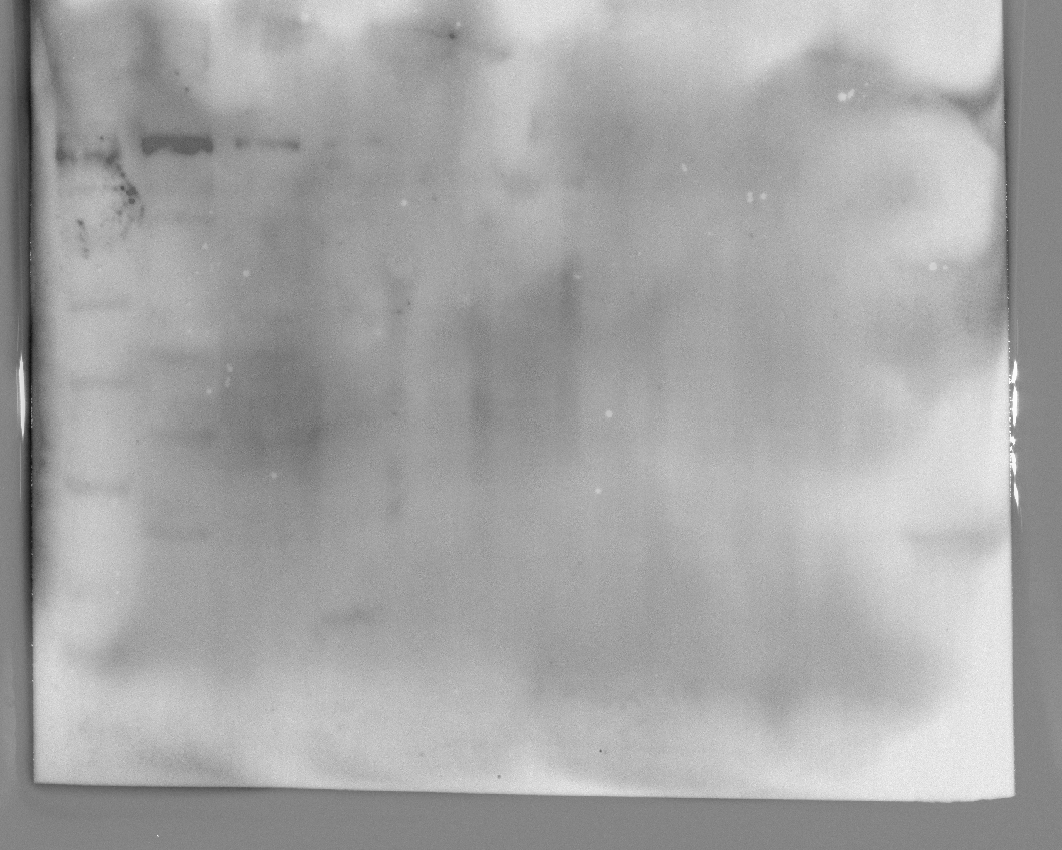

Supplement: Figure 1—source data 1. [file elife-85131-fig1-data1.zip › Figure 1-source data 1/Figure 1-raw gel image/Fig1B Adgrf1.tif]

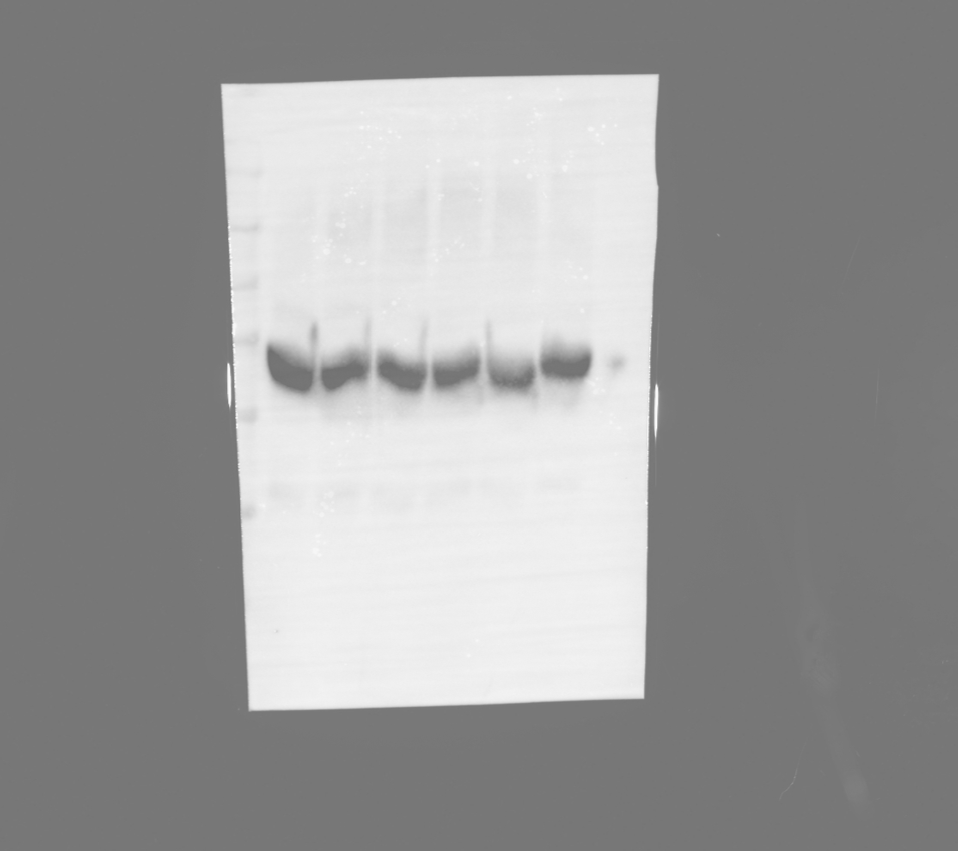

Supplement: Figure 1—source data 1. [file elife-85131-fig1-data1.zip › Figure 1-source data 1/Figure 1-raw gel image/Fig1G b-tubulin (kidney).tif]

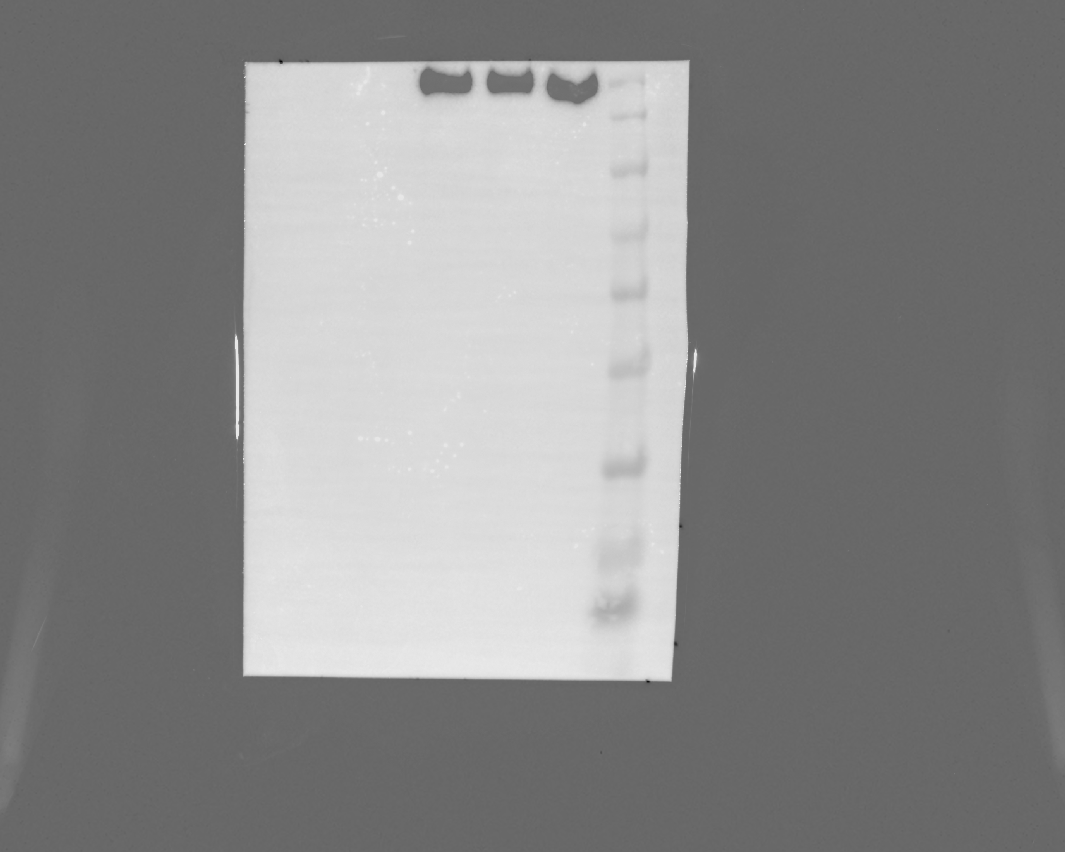

Supplement: Figure 1—source data 1. [file elife-85131-fig1-data1.zip › Figure 1-source data 1/Figure 1-raw gel image/Fig1D Cd11b.tif]

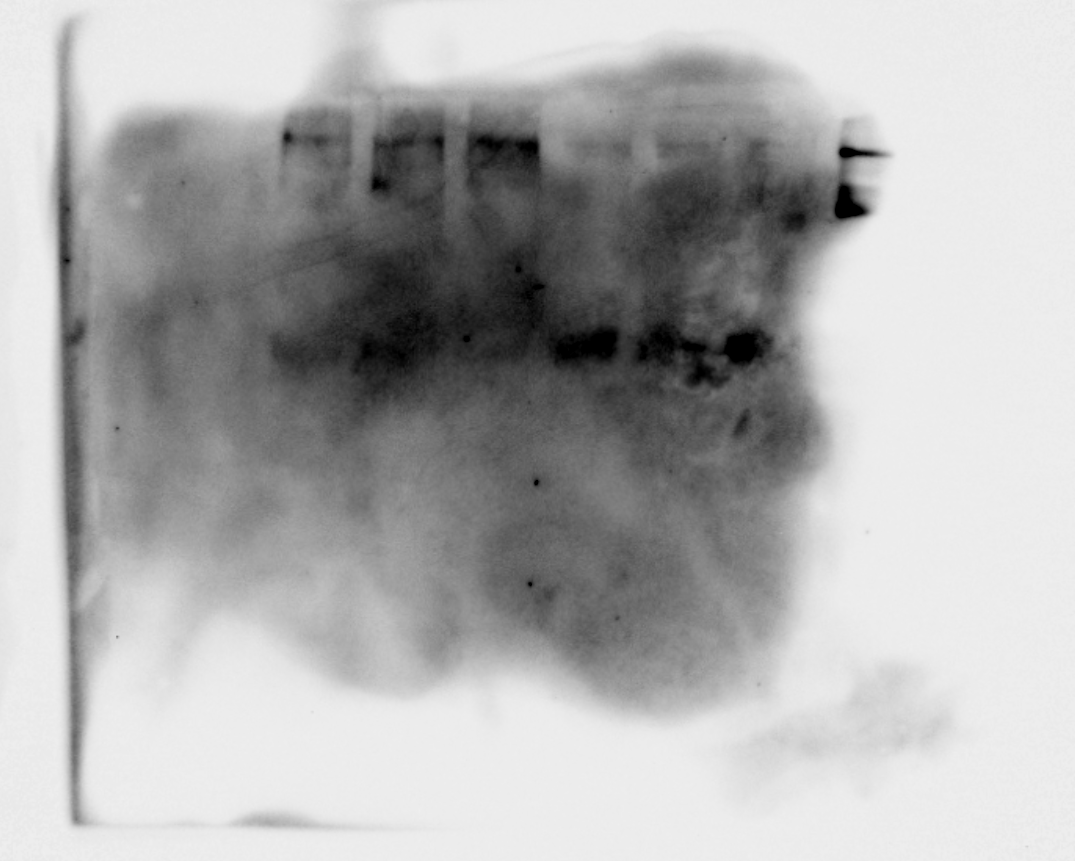

Supplement: Figure 1—source data 1. [file elife-85131-fig1-data1.zip › Figure 1-source data 1/Figure 1-raw gel image/Fig1D Adgrf1.tif]

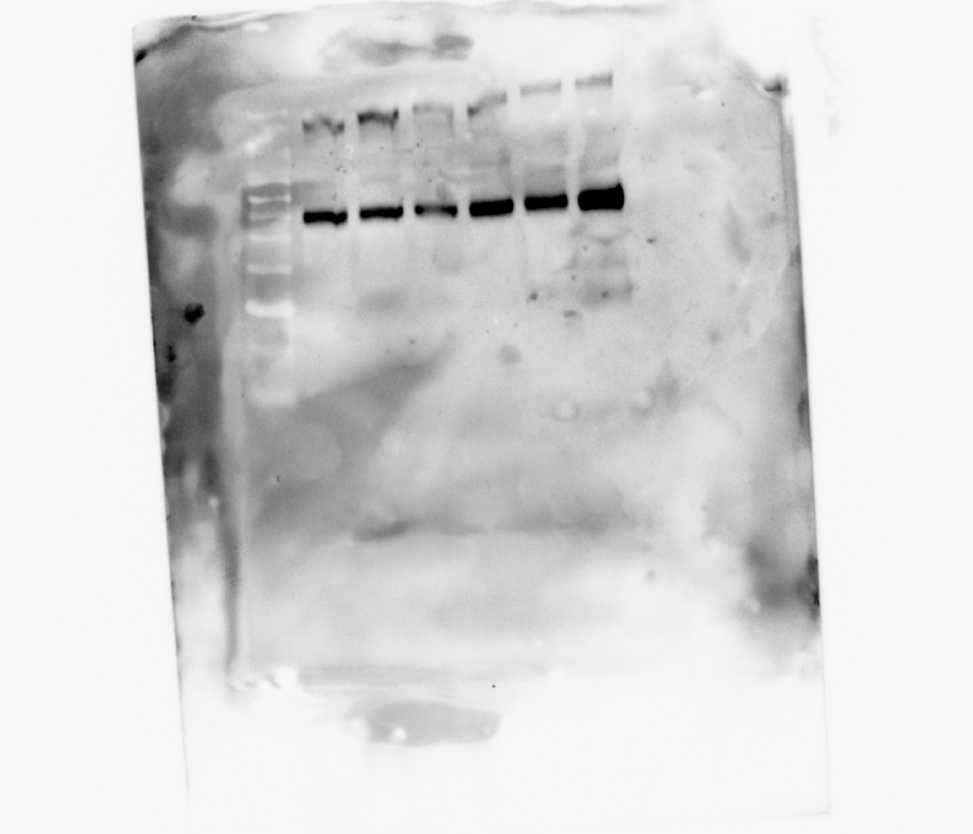

Supplement: Figure 1—source data 1. [file elife-85131-fig1-data1.zip › Figure 1-source data 1/Figure 1-raw gel image/Fig1G b-tubulin (liver).tif]

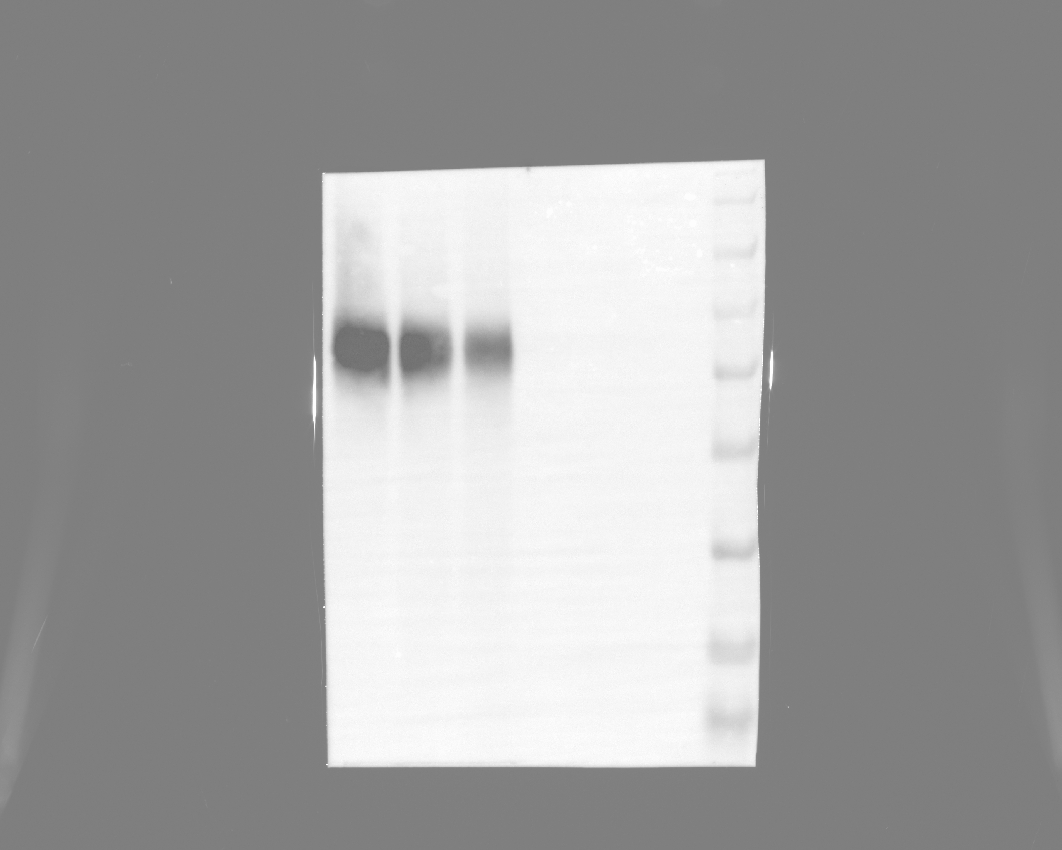

Supplement: Figure 1—source data 1. [file elife-85131-fig1-data1.zip › Figure 1-source data 1/Figure 1-raw gel image/Fig1D Albumin.tif]

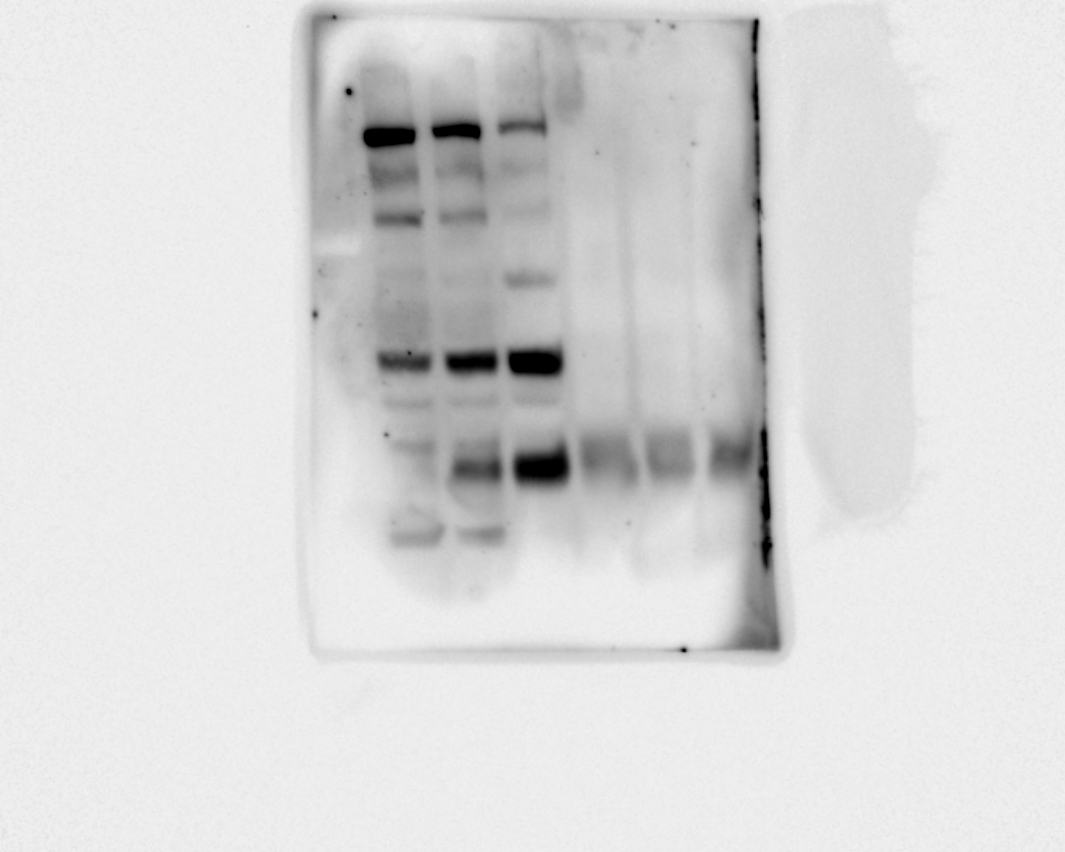

Supplement: Figure 1—source data 1. [file elife-85131-fig1-data1.zip › Figure 1-source data 1/Figure 1-raw gel image/Fig1G Adgrf1 (liver).tif]

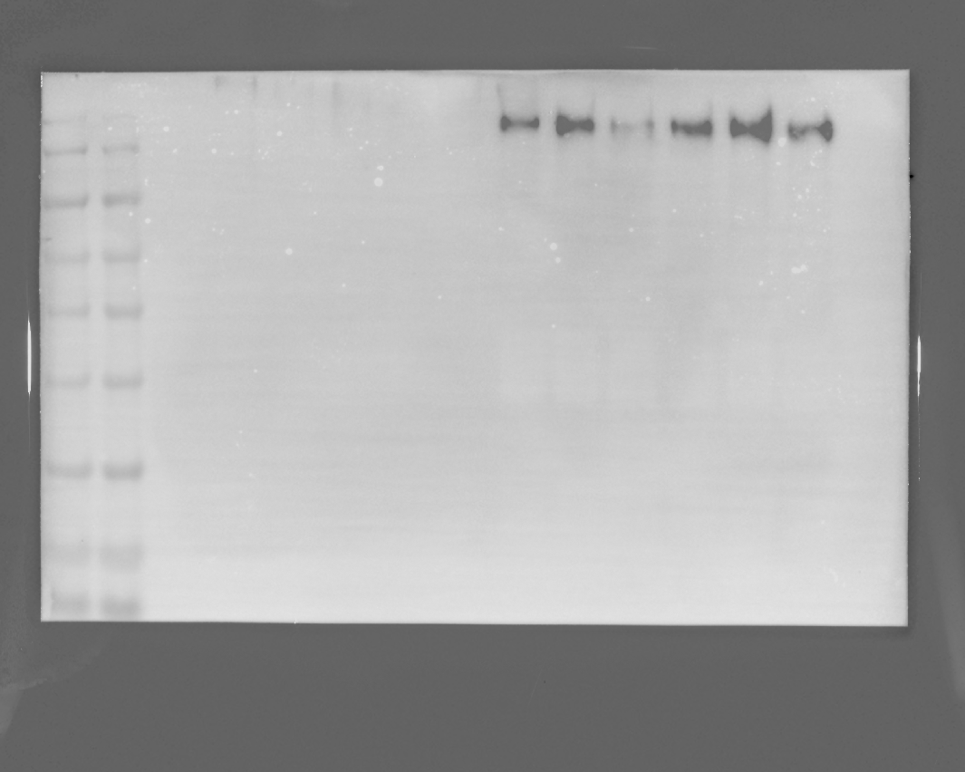

Supplement: Figure 2—source data 1. [file elife-85131-fig2-data1.zip › Figure 2-source data 1/Figure 2-raw gel image/Fig 2B Cd11b.tif]

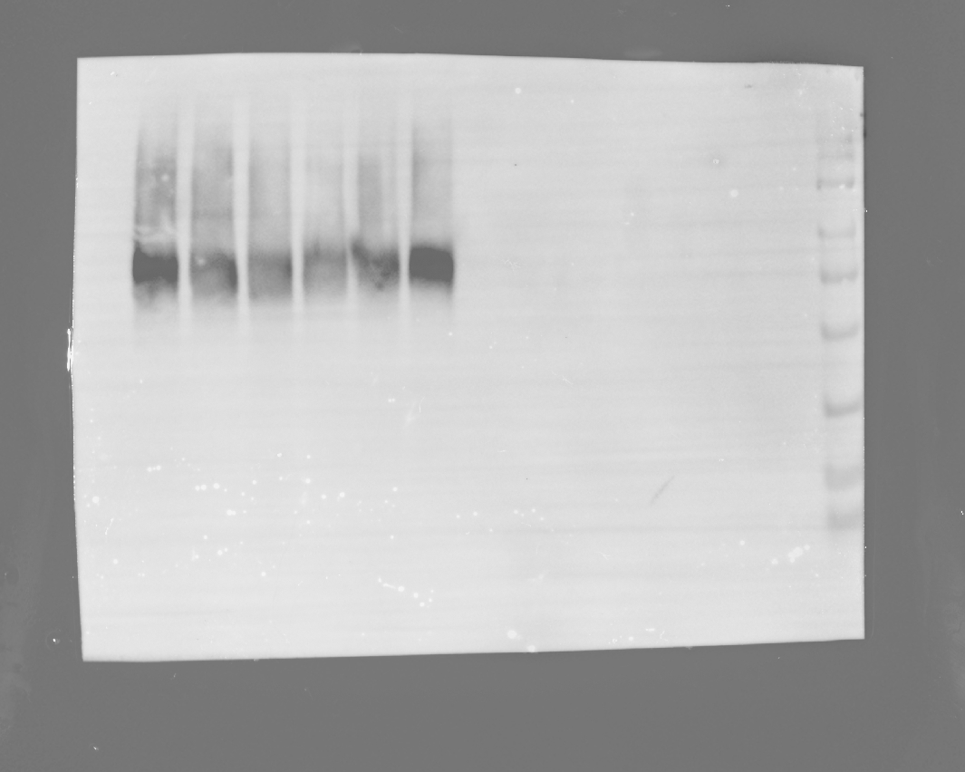

Supplement: Figure 2—source data 1. [file elife-85131-fig2-data1.zip › Figure 2-source data 1/Figure 2-raw gel image/Fig 2B Albumin.tif]

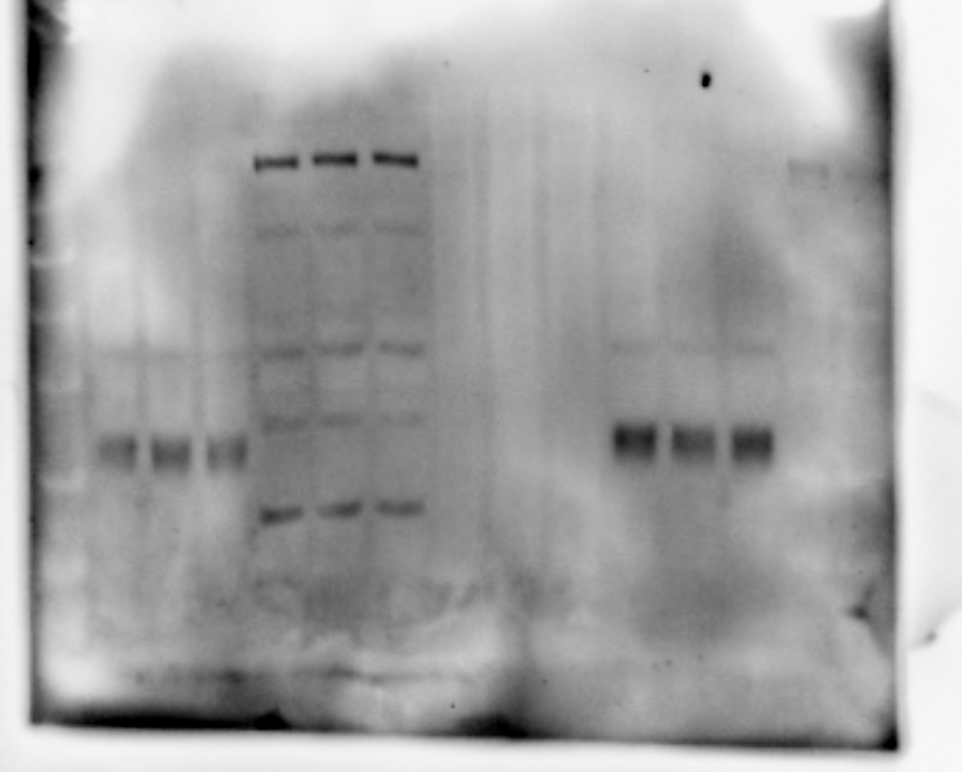

Supplement: Figure 2—source data 1. [file elife-85131-fig2-data1.zip › Figure 2-source data 1/Figure 2-raw gel image/Fig 2B Adgrf1.tif]

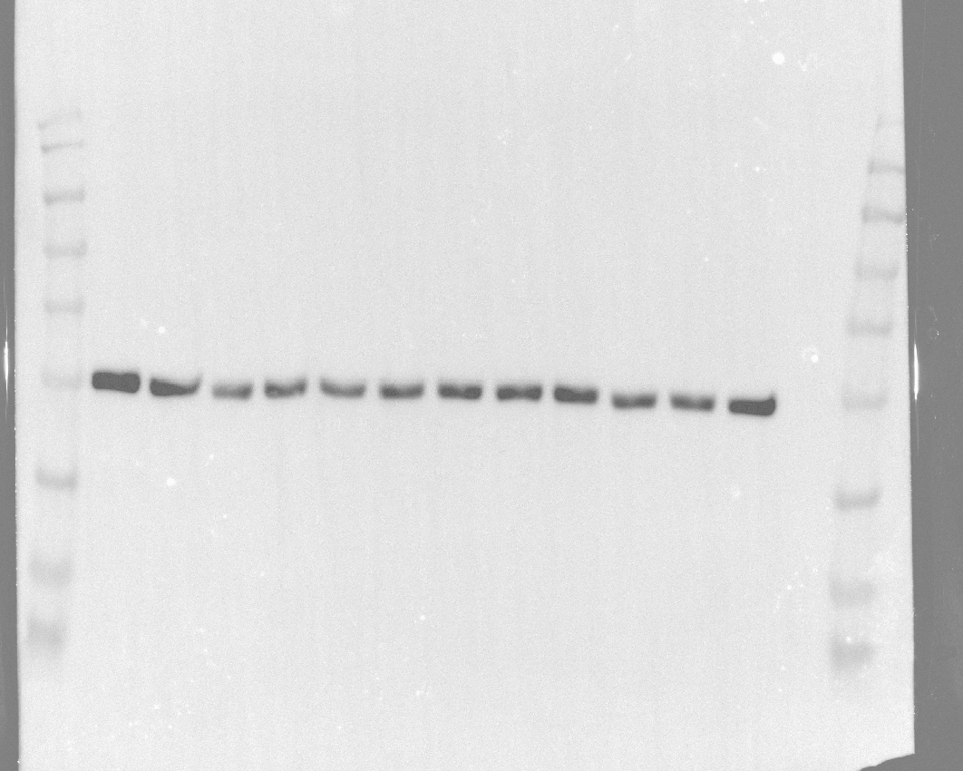

Supplement: Figure 2—source data 1. [file elife-85131-fig2-data1.zip › Figure 2-source data 1/Figure 2-raw gel image/Fig 2B b-actin.tif]

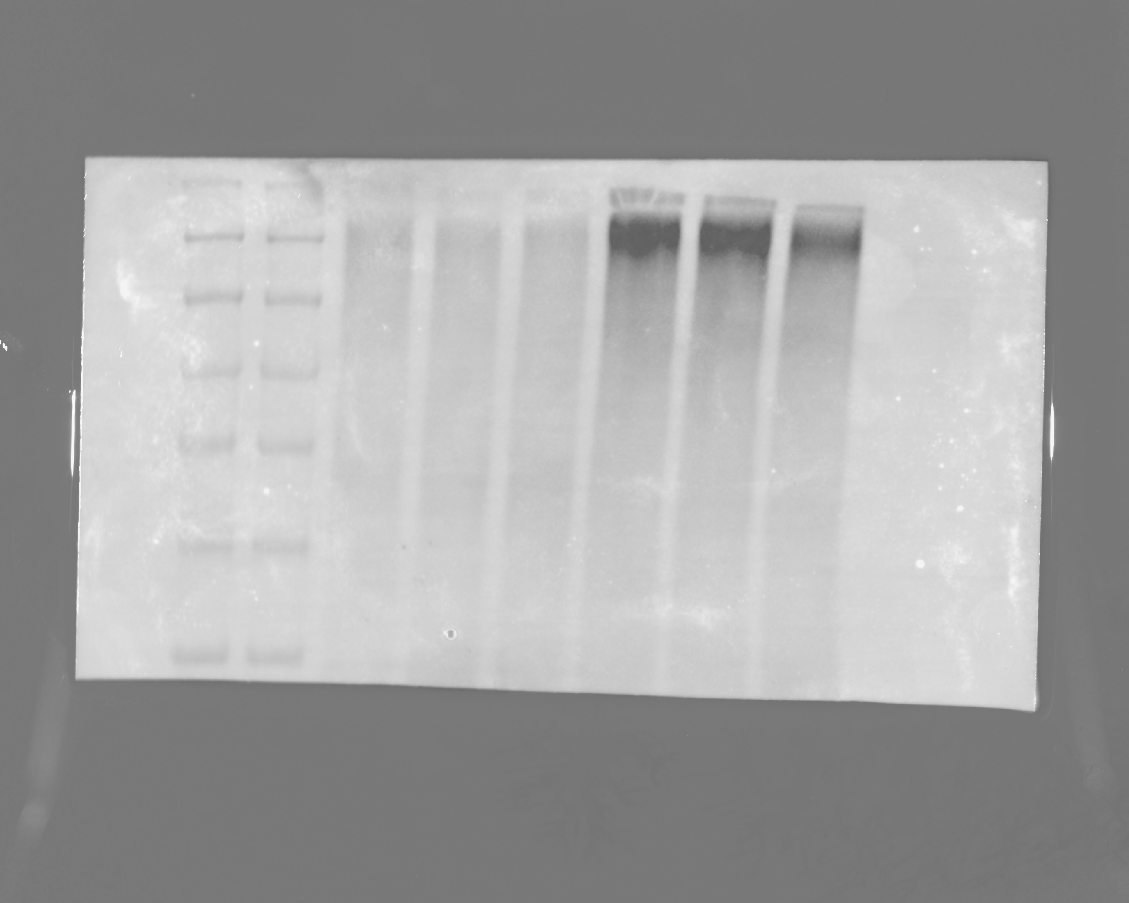

Supplement: Figure 2—figure supplement 1—source data 1. [file elife-85131-fig2-figsupp1-data1.zip › Figure 2-figure supplement 1-source data 1/Figure 2-figure supplement 1-raw gel image/SFig 1C Flag (liver).tif]

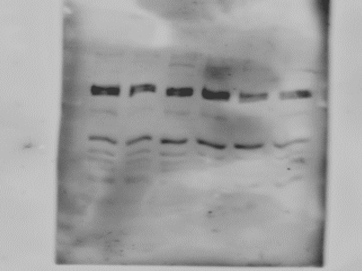

Supplement: Figure 2—figure supplement 1—source data 1. [file elife-85131-fig2-figsupp1-data1.zip › Figure 2-figure supplement 1-source data 1/Figure 2-figure supplement 1-raw gel image/SFig 1C Adgrf1 (kidney).tif]

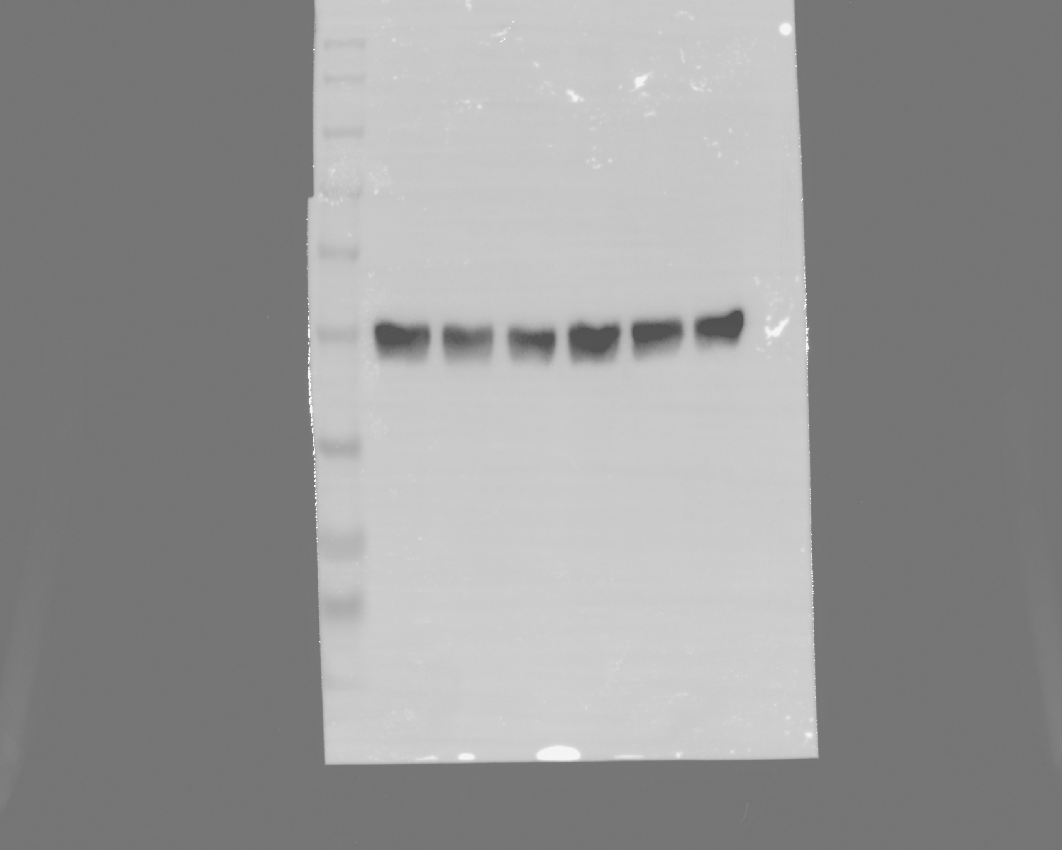

Supplement: Figure 2—figure supplement 1—source data 1. [file elife-85131-fig2-figsupp1-data1.zip › Figure 2-figure supplement 1-source data 1/Figure 2-figure supplement 1-raw gel image/SFig 1C b-actin (liver).tif]

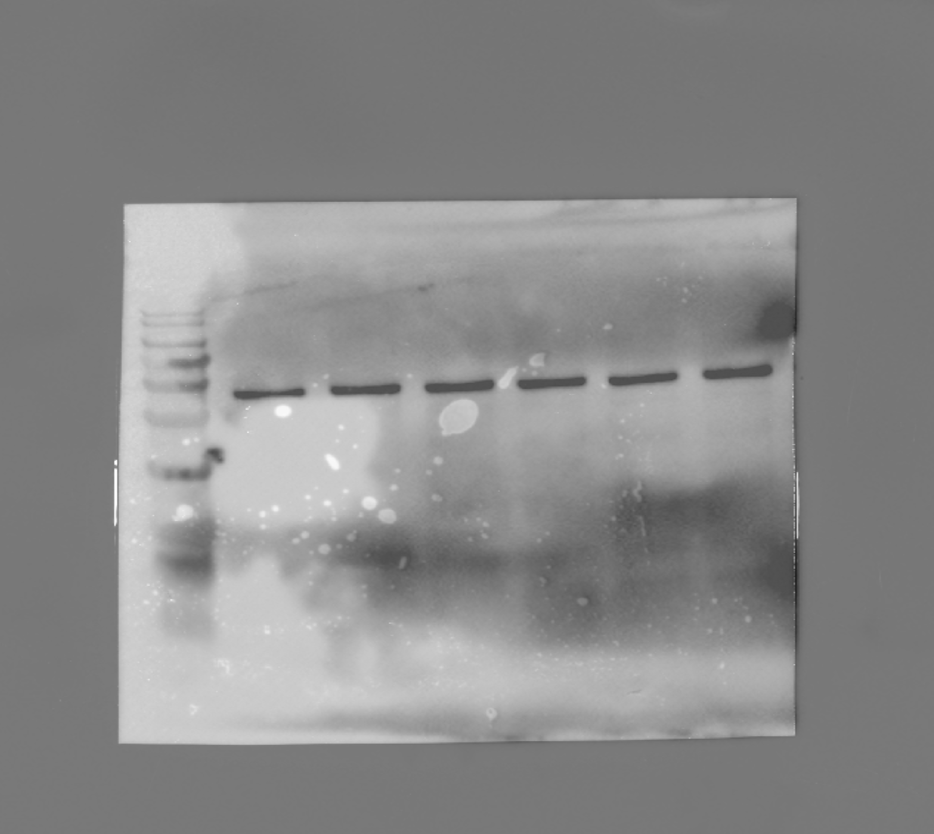

Supplement: Figure 2—figure supplement 1—source data 1. [file elife-85131-fig2-figsupp1-data1.zip › Figure 2-figure supplement 1-source data 1/Figure 2-figure supplement 1-raw gel image/SFig 1C b-actin (kidney).tif]

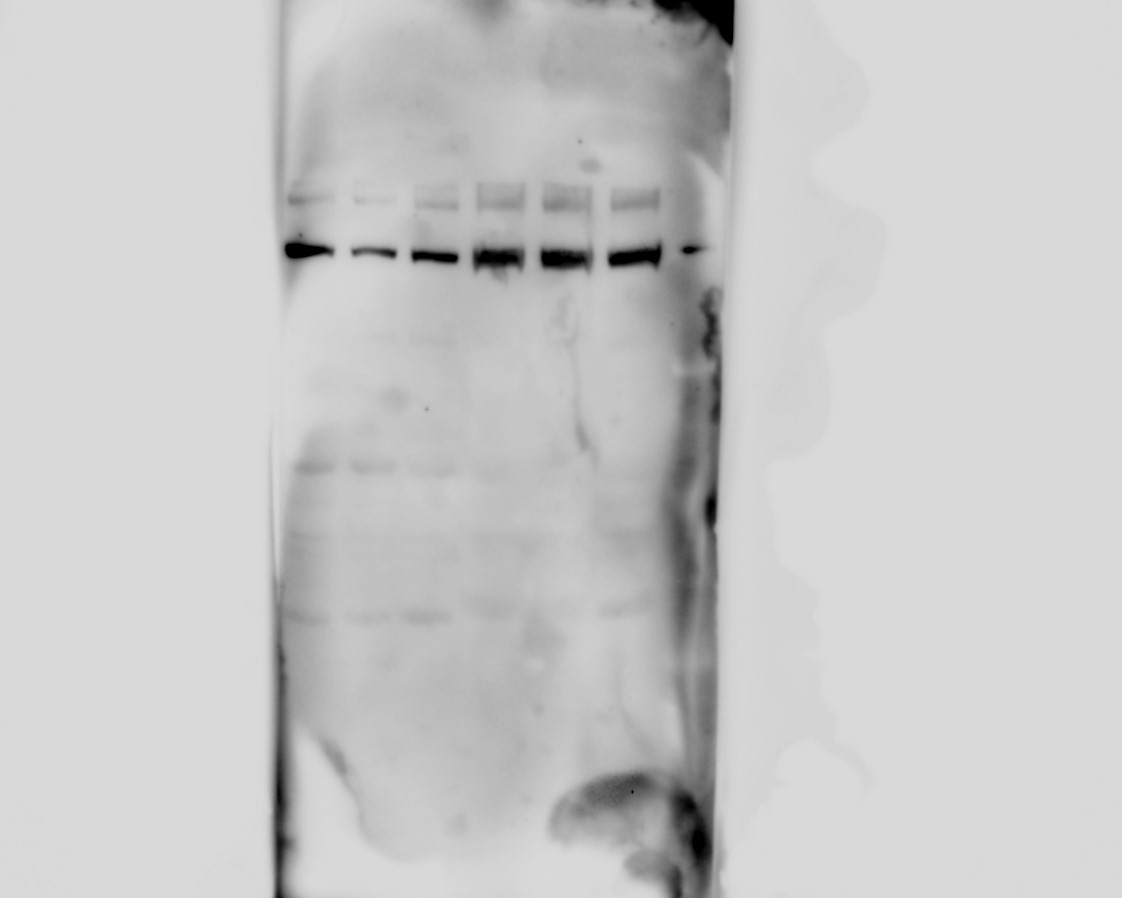

Supplement: Figure 2—figure supplement 1—source data 1. [file elife-85131-fig2-figsupp1-data1.zip › Figure 2-figure supplement 1-source data 1/Figure 2-figure supplement 1-raw gel image/SFig 1C Adgrf1 (liver).tif]

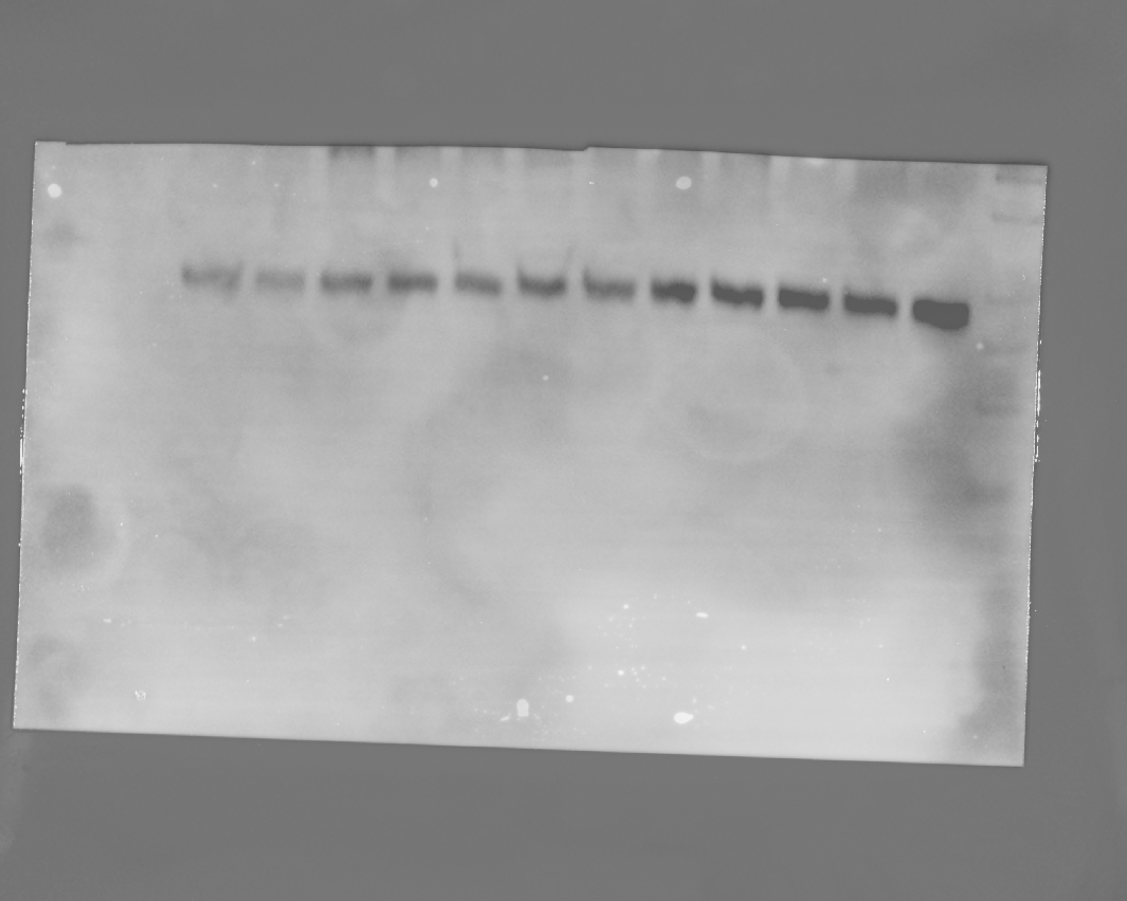

Supplement: Figure 3—source data 1. [file elife-85131-fig3-data1.zip › Figure 3-source data 1/Figure 3-raw gel image/Fig 3C b-tubulin.tif]

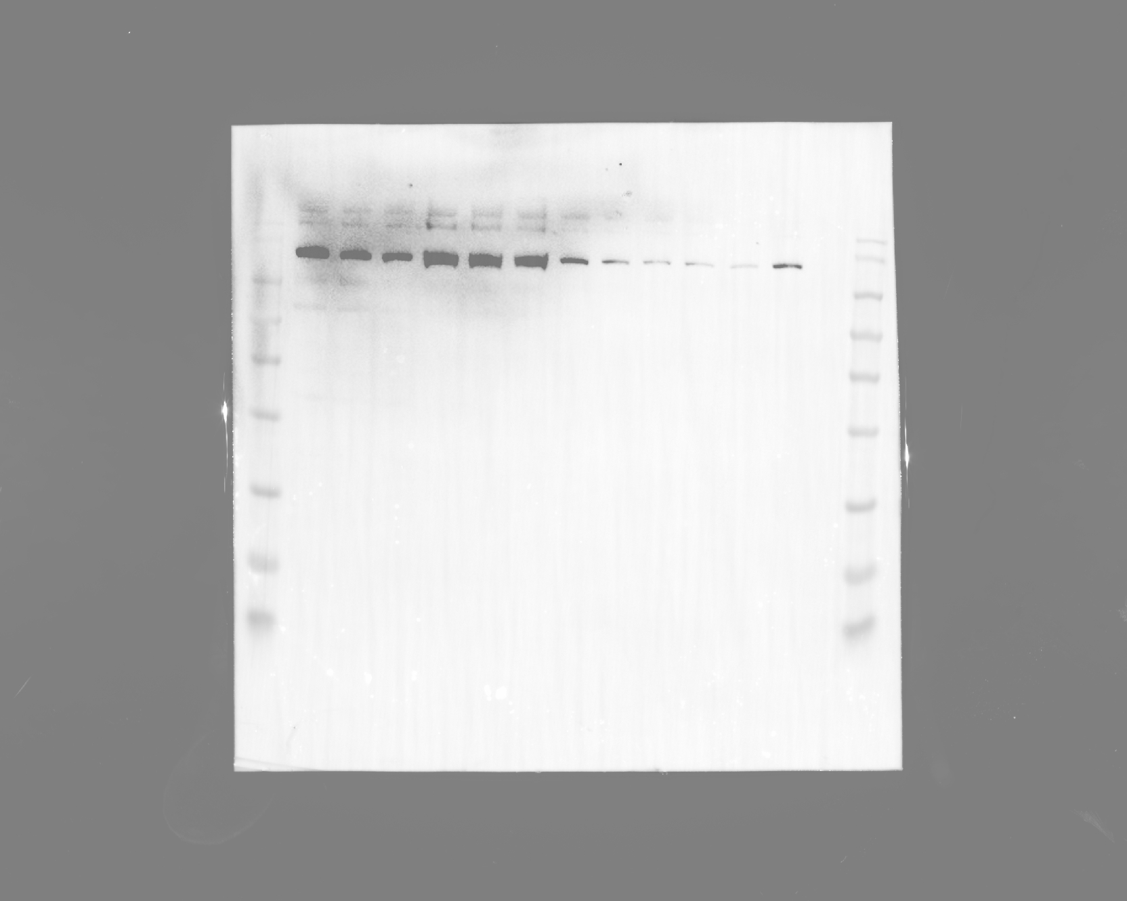

Supplement: Figure 3—source data 1. [file elife-85131-fig3-data1.zip › Figure 3-source data 1/Figure 3-raw gel image/Fig 3C Adgrf1.tif]

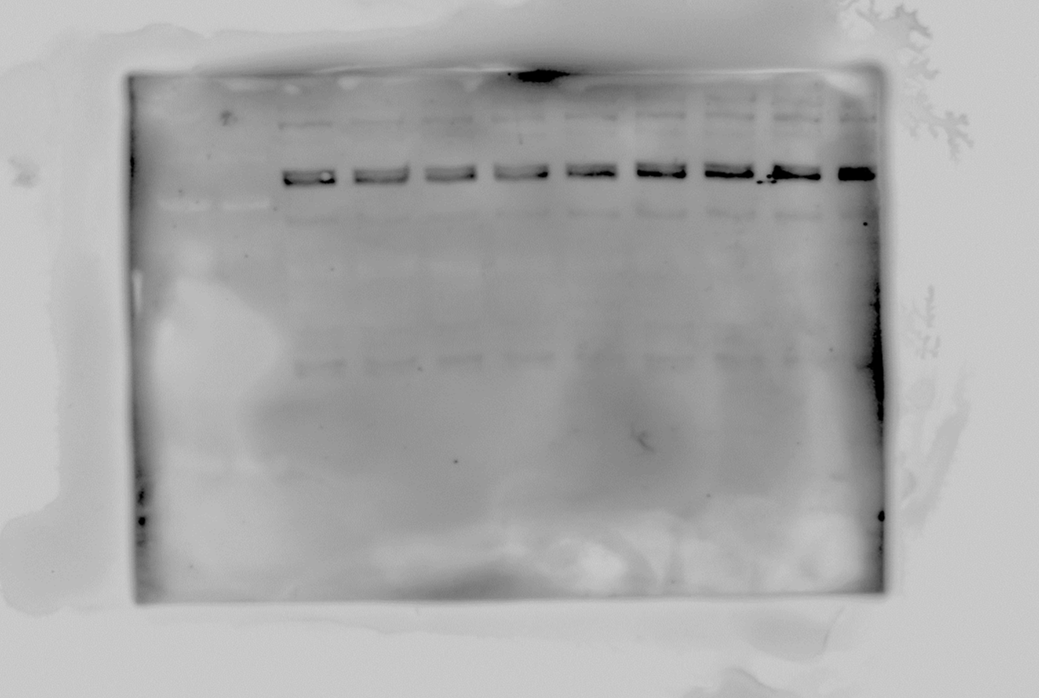

Supplement: Figure 3—figure supplement 1—source data 1. [file elife-85131-fig3-figsupp1-data1.zip › Figure 3-figure supplement 1-source data 1/Figure 3-figure supplement 1-raw gel image/SFig 3C Adgrf1 (kidney).tif]

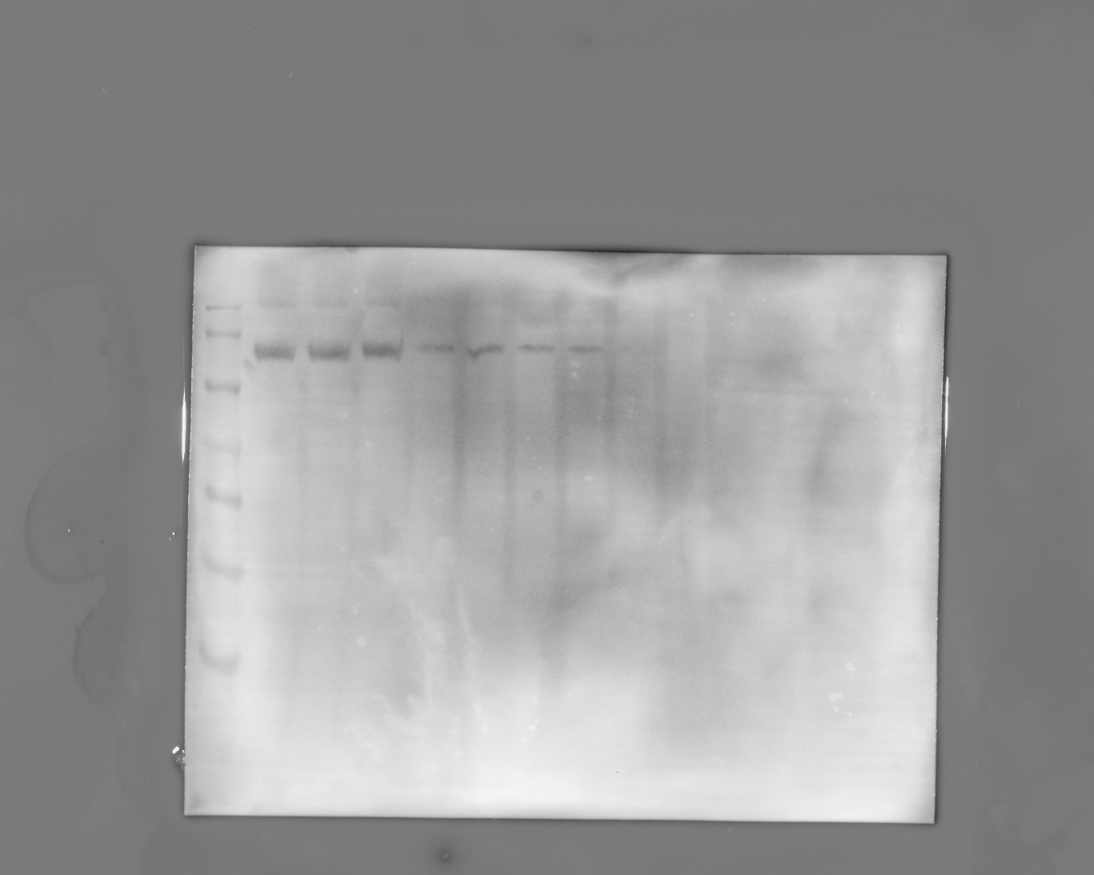

Supplement: Figure 3—figure supplement 1—source data 1. [file elife-85131-fig3-figsupp1-data1.zip › Figure 3-figure supplement 1-source data 1/Figure 3-figure supplement 1-raw gel image/SFig 3C Adgrf1 (liver).tif]

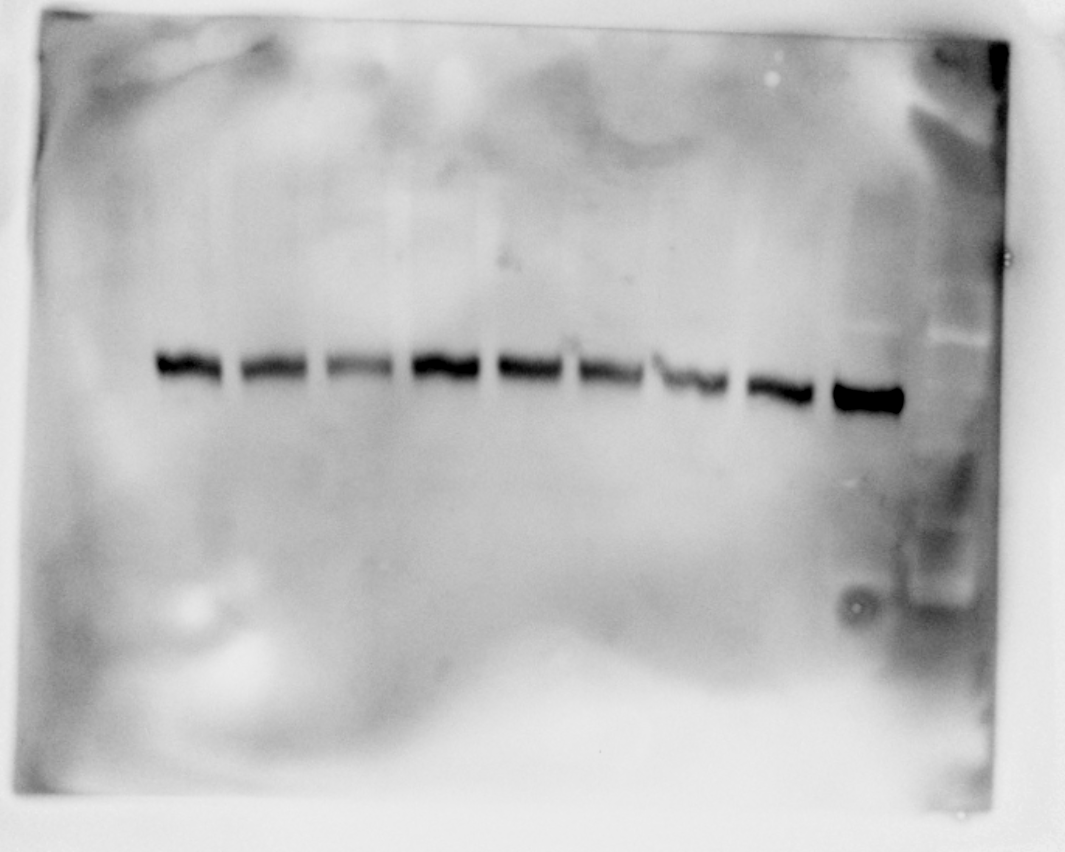

Supplement: Figure 3—figure supplement 1—source data 1. [file elife-85131-fig3-figsupp1-data1.zip › Figure 3-figure supplement 1-source data 1/Figure 3-figure supplement 1-raw gel image/SFig 3C b-tubulin (kidney).tif]

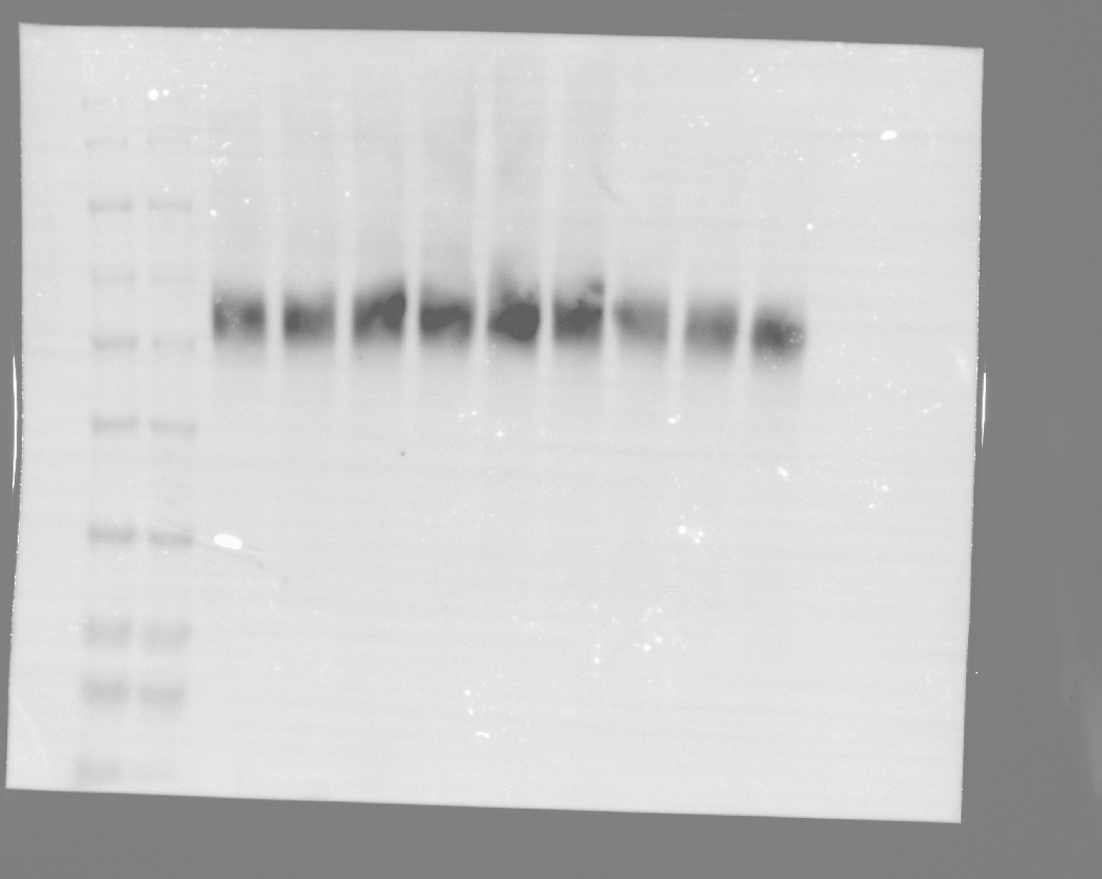

Supplement: Figure 3—figure supplement 1—source data 1. [file elife-85131-fig3-figsupp1-data1.zip › Figure 3-figure supplement 1-source data 1/Figure 3-figure supplement 1-raw gel image/SFig 3C b-tubulin (liver).tif]

## Slide 1
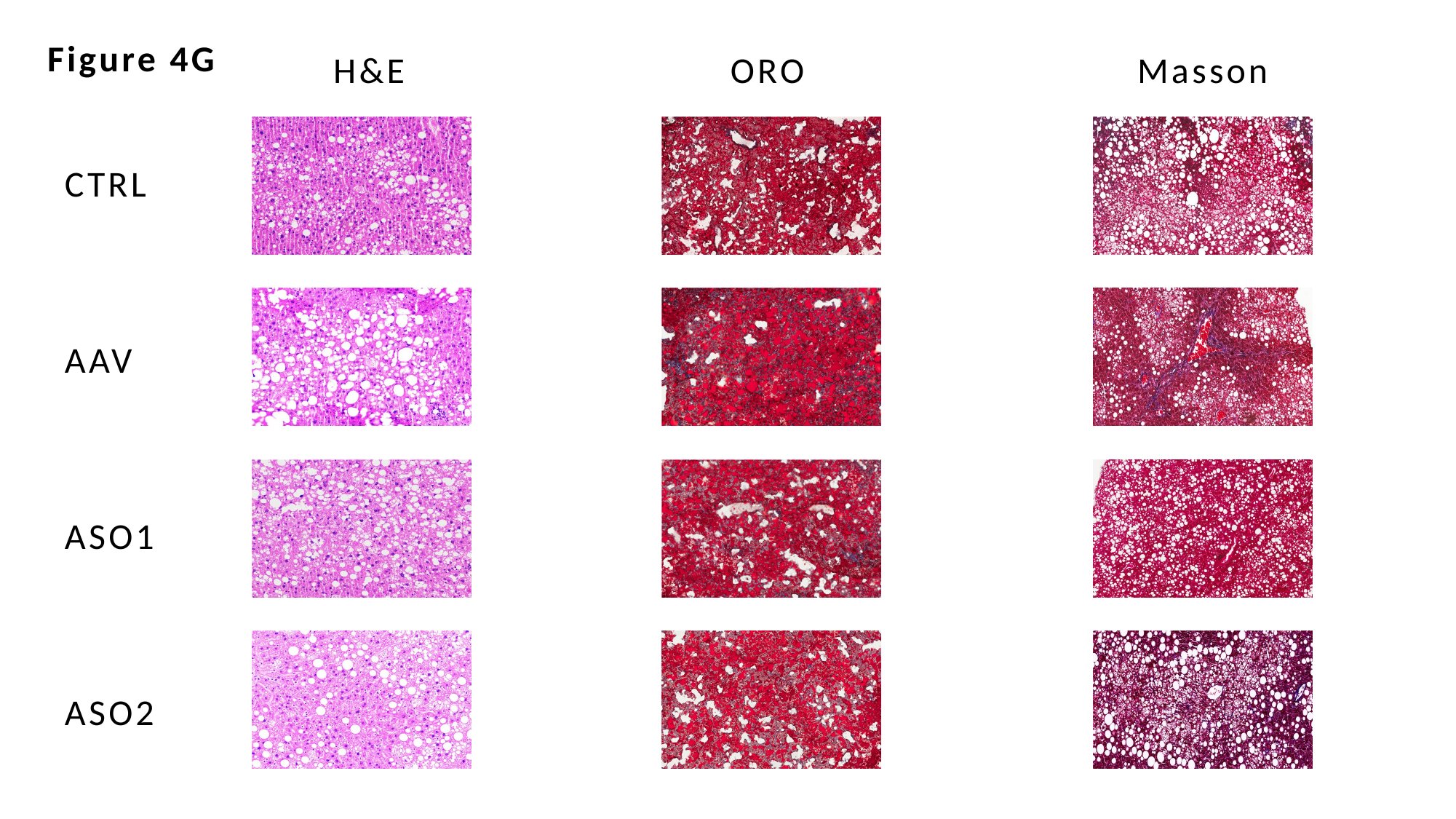

Figure 4G
# H&E
ORO
Masson
CTRL
AAV
ASO1
ASO2

Supplement: Figure 4—source data 1. [file elife-85131-fig4-data1.zip › Figure 4-source data 1/Figure 4-Source 2.pptx]

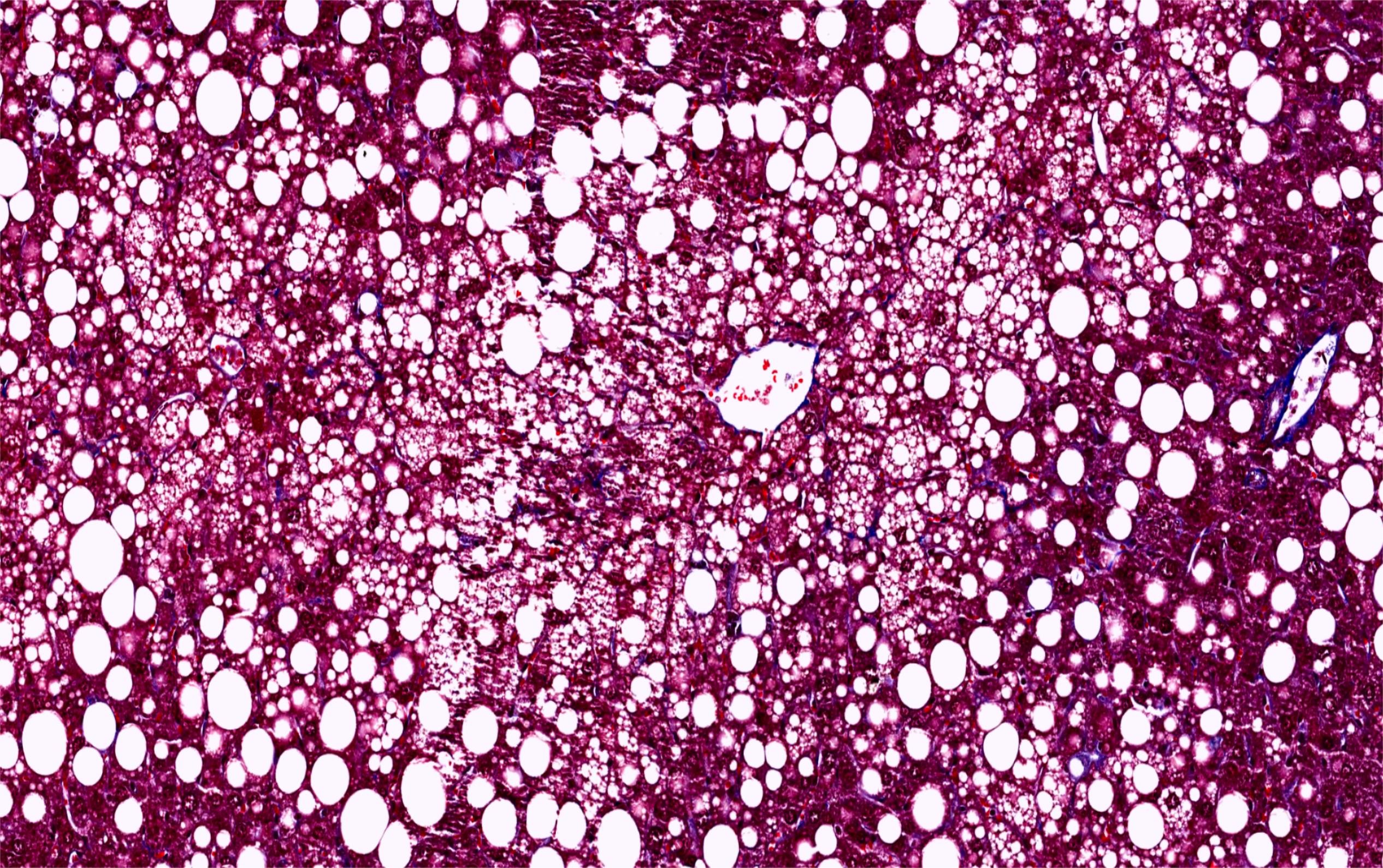

Supplement: Figure 4—source data 1. [file elife-85131-fig4-data1.zip › Figure 4-source data 1/Figure 4-raw microscopy images/Masson/ASO2.jpg]

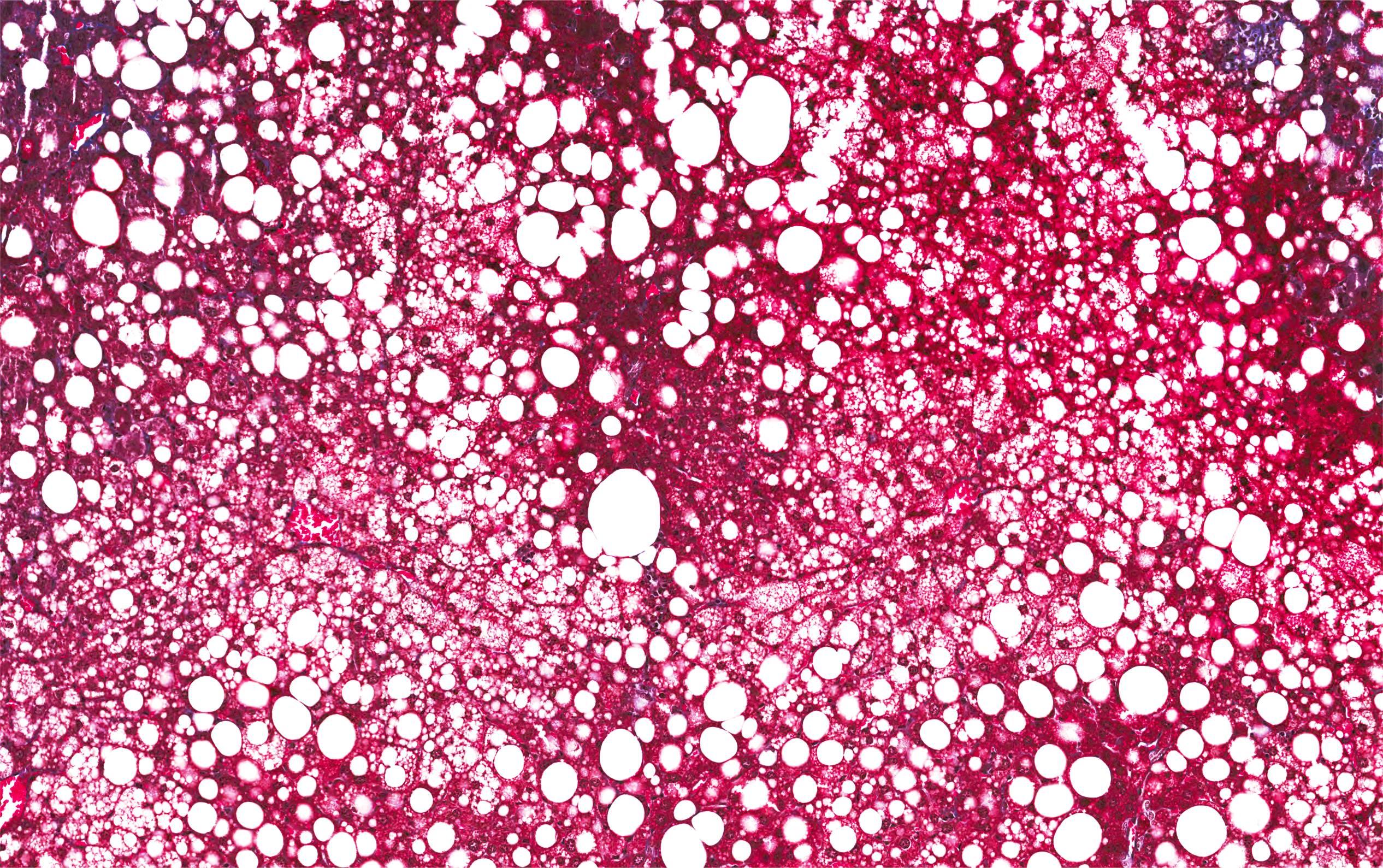

Supplement: Figure 4—source data 1. [file elife-85131-fig4-data1.zip › Figure 4-source data 1/Figure 4-raw microscopy images/Masson/CTRL.jpg]

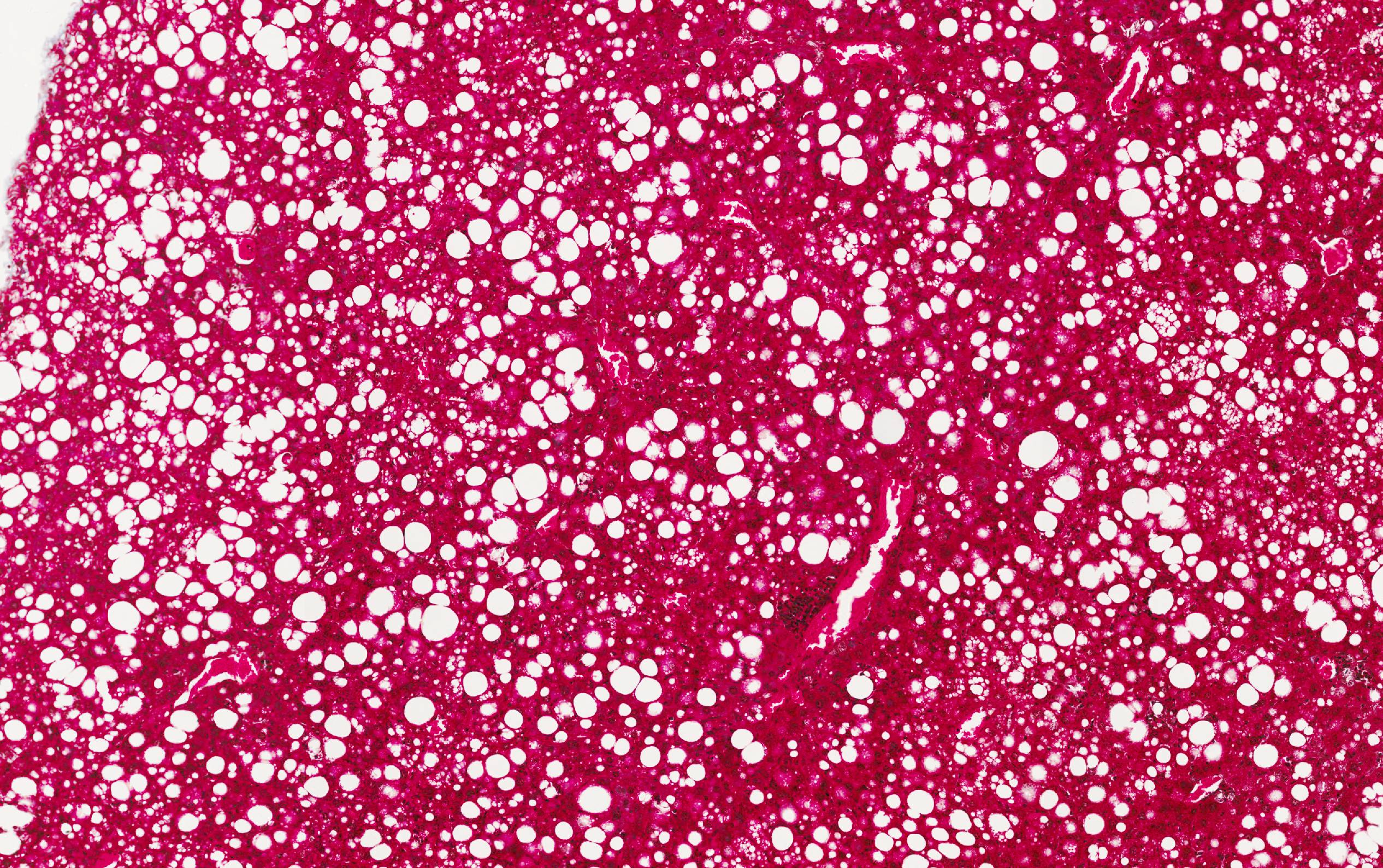

Supplement: Figure 4—source data 1. [file elife-85131-fig4-data1.zip › Figure 4-source data 1/Figure 4-raw microscopy images/Masson/ASO1.jpg]

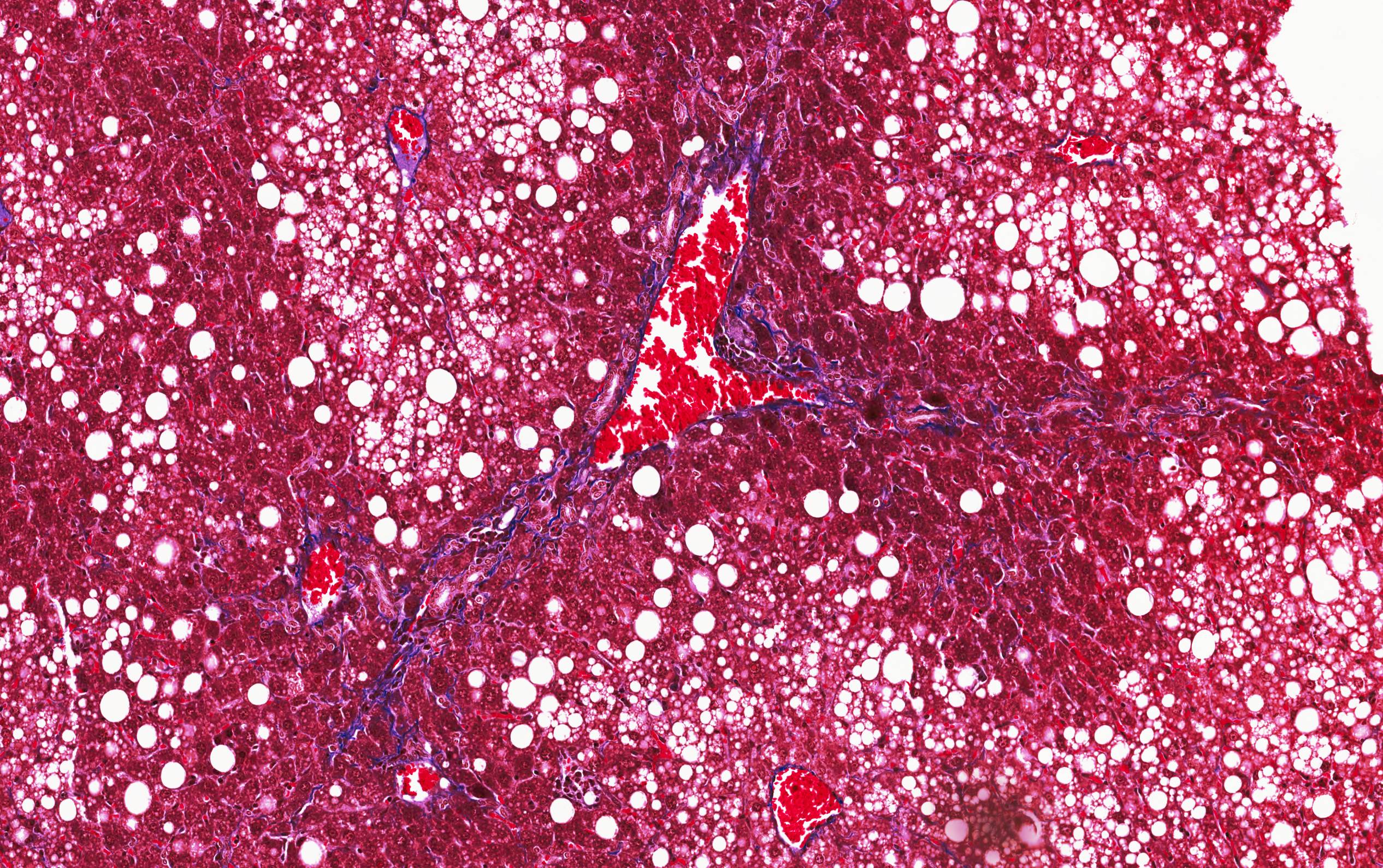

Supplement: Figure 4—source data 1. [file elife-85131-fig4-data1.zip › Figure 4-source data 1/Figure 4-raw microscopy images/Masson/AAV.jpg]

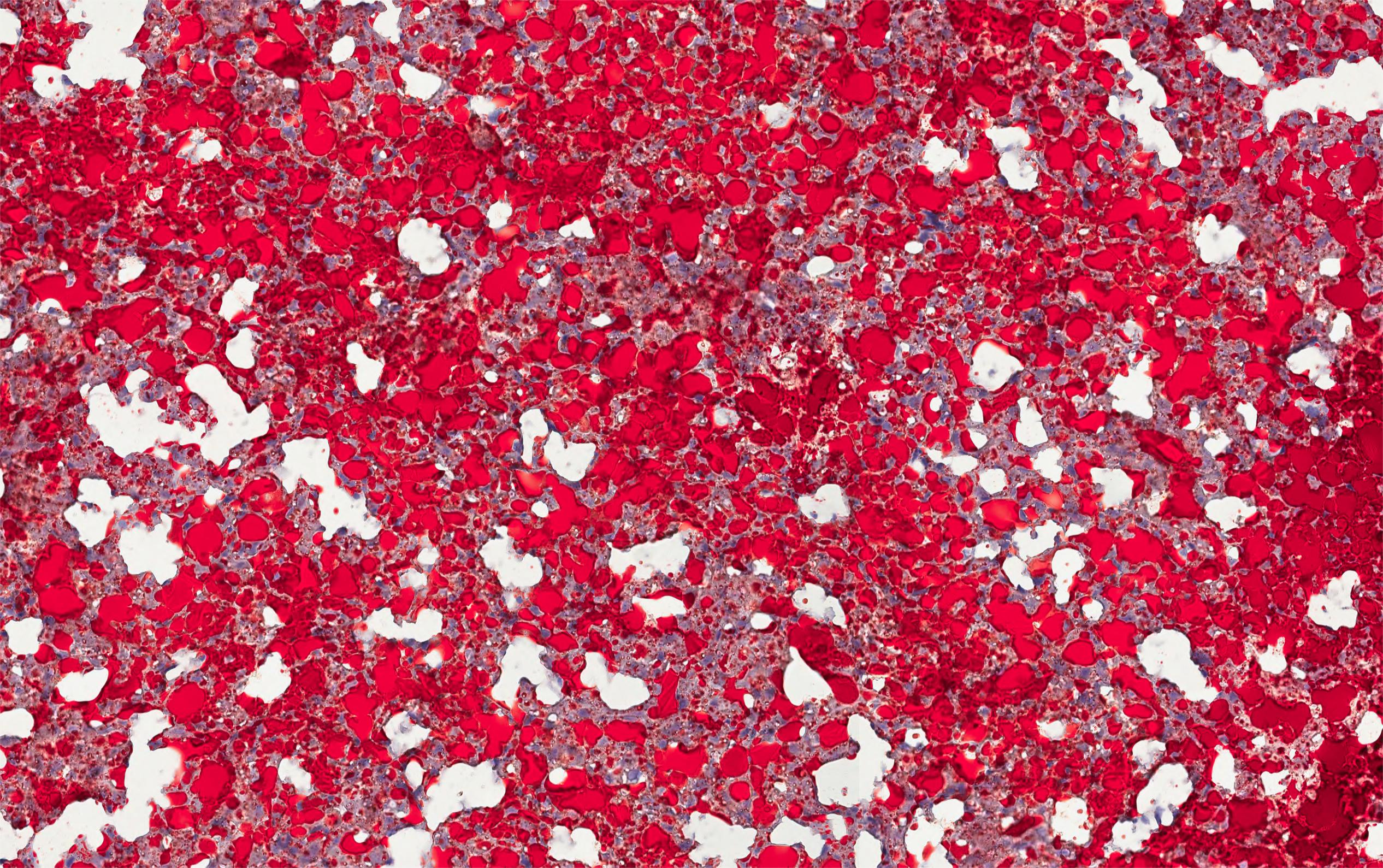

Supplement: Figure 4—source data 1. [file elife-85131-fig4-data1.zip › Figure 4-source data 1/Figure 4-raw microscopy images/Oil Red O/ASO2.jpg]

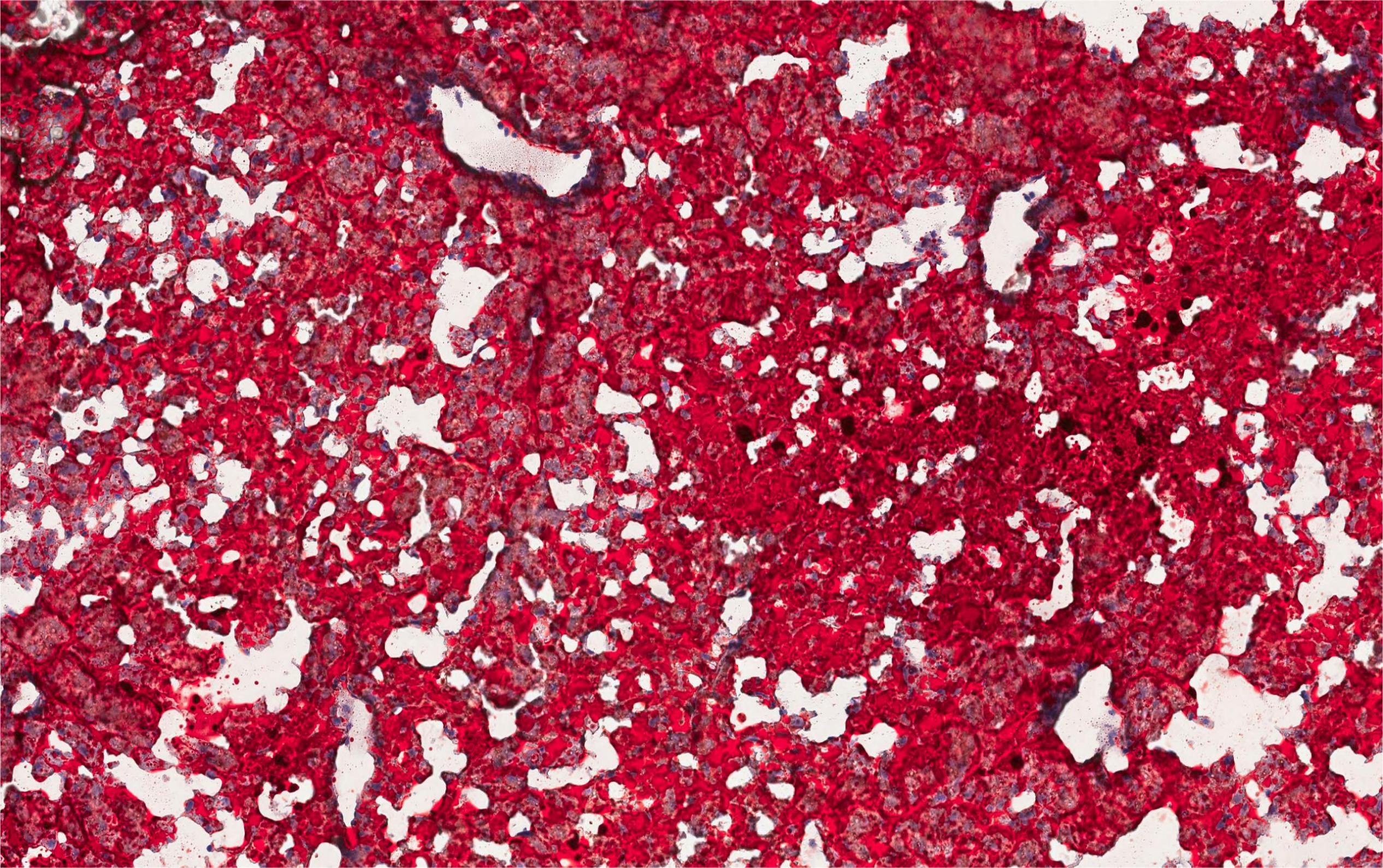

Supplement: Figure 4—source data 1. [file elife-85131-fig4-data1.zip › Figure 4-source data 1/Figure 4-raw microscopy images/Oil Red O/CTRL.jpg]

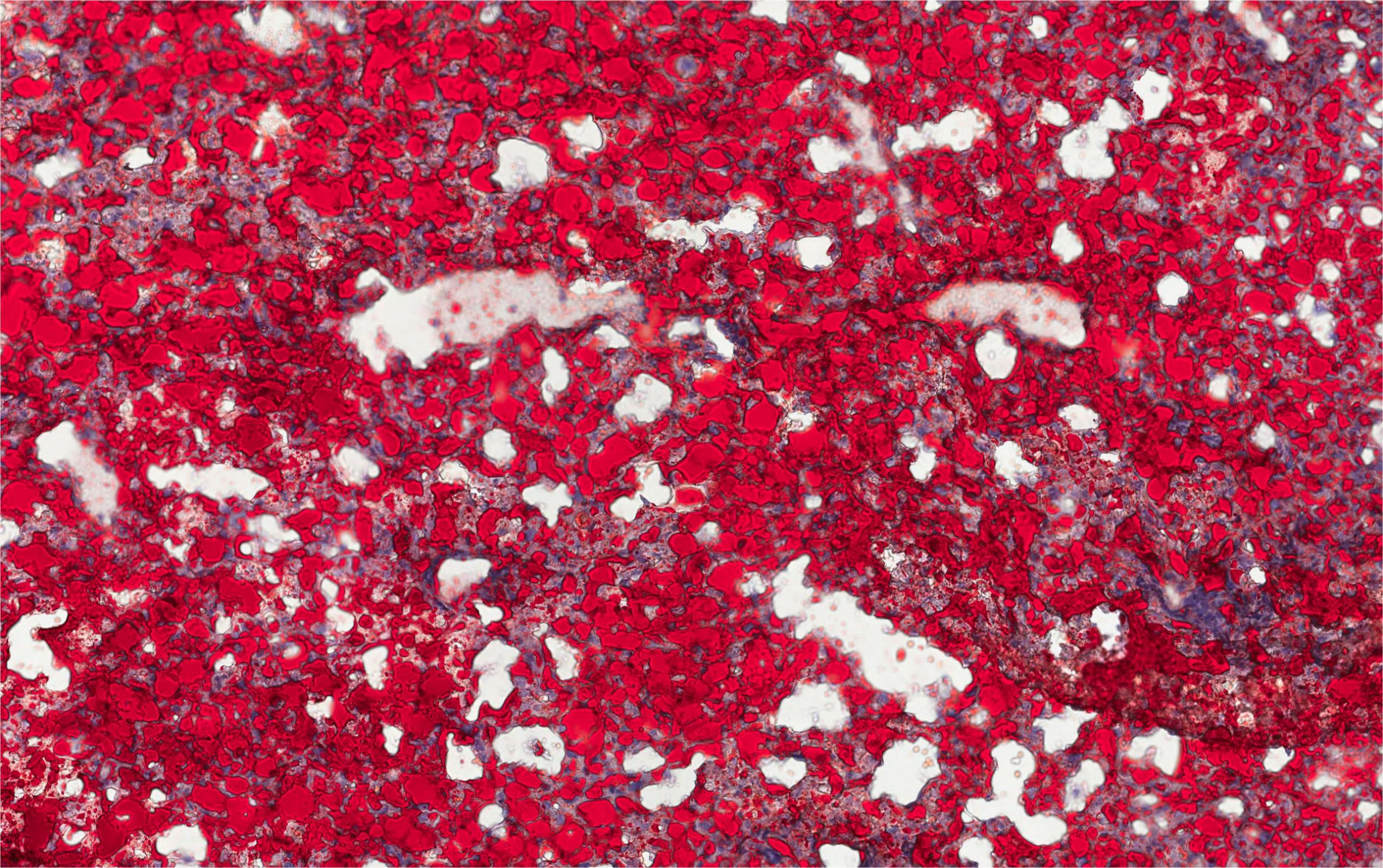

Supplement: Figure 4—source data 1. [file elife-85131-fig4-data1.zip › Figure 4-source data 1/Figure 4-raw microscopy images/Oil Red O/ASO1.jpg]

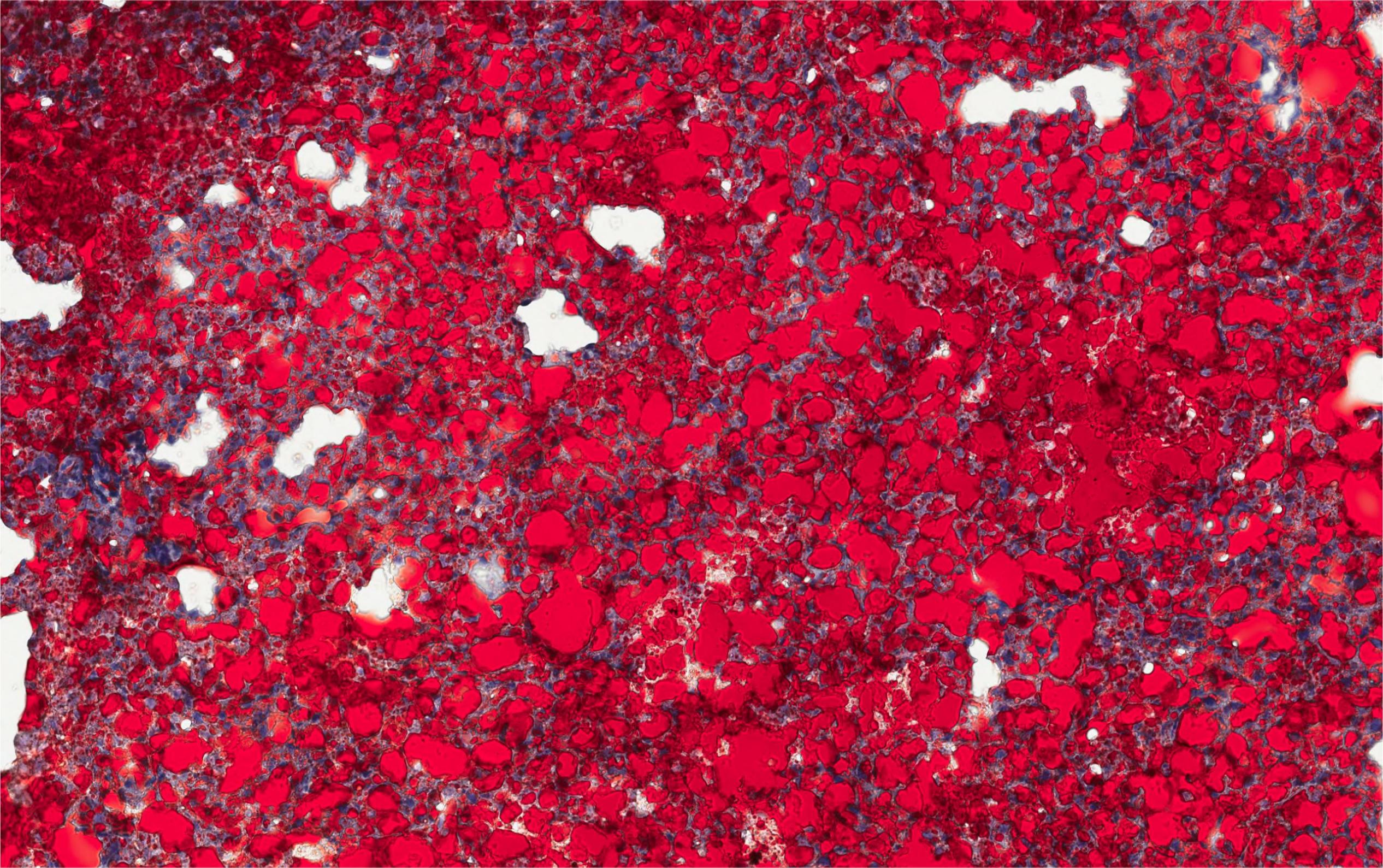

Supplement: Figure 4—source data 1. [file elife-85131-fig4-data1.zip › Figure 4-source data 1/Figure 4-raw microscopy images/Oil Red O/AAV.jpg]

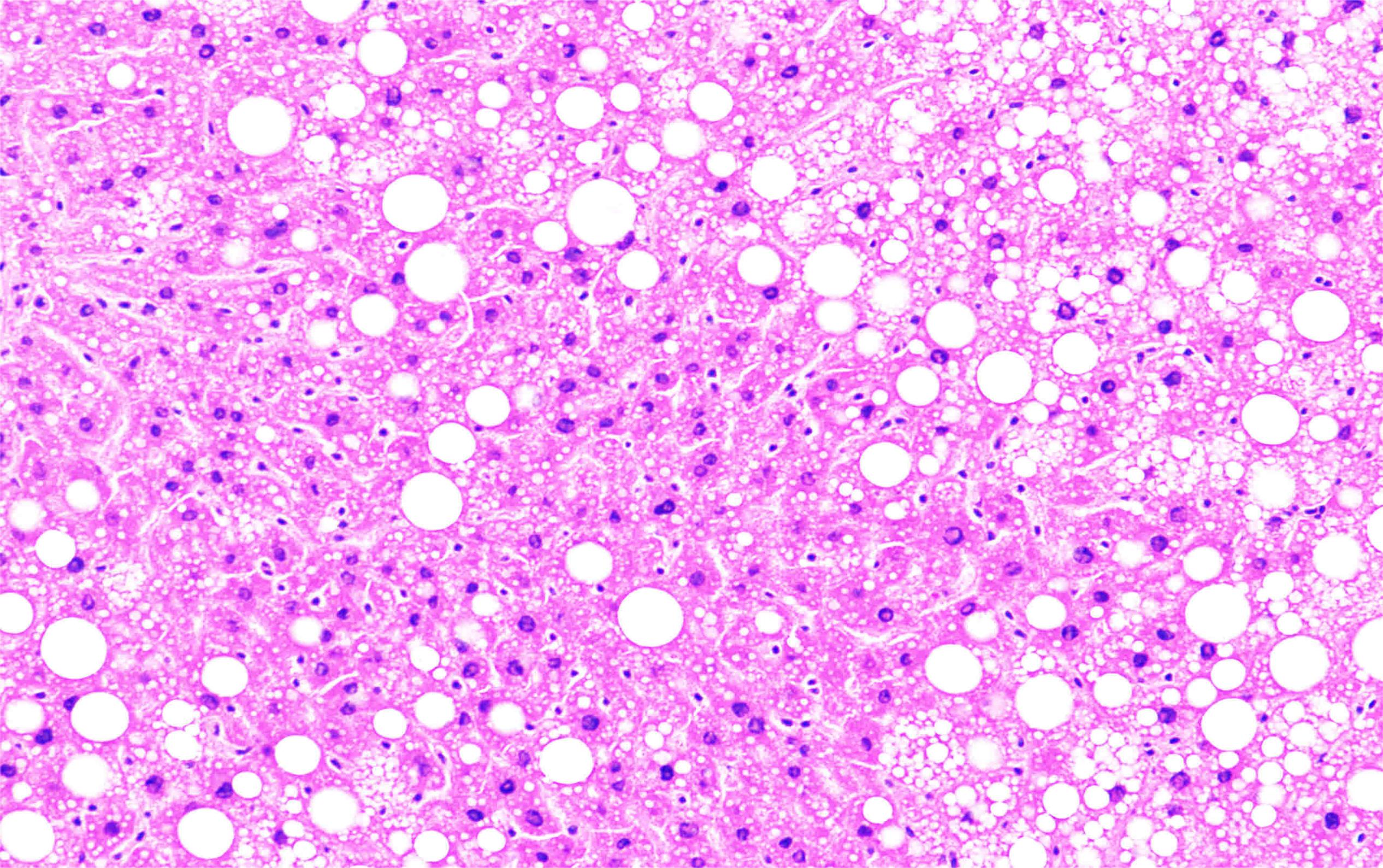

Supplement: Figure 4—source data 1. [file elife-85131-fig4-data1.zip › Figure 4-source data 1/Figure 4-raw microscopy images/H_E/ASO2.jpg]

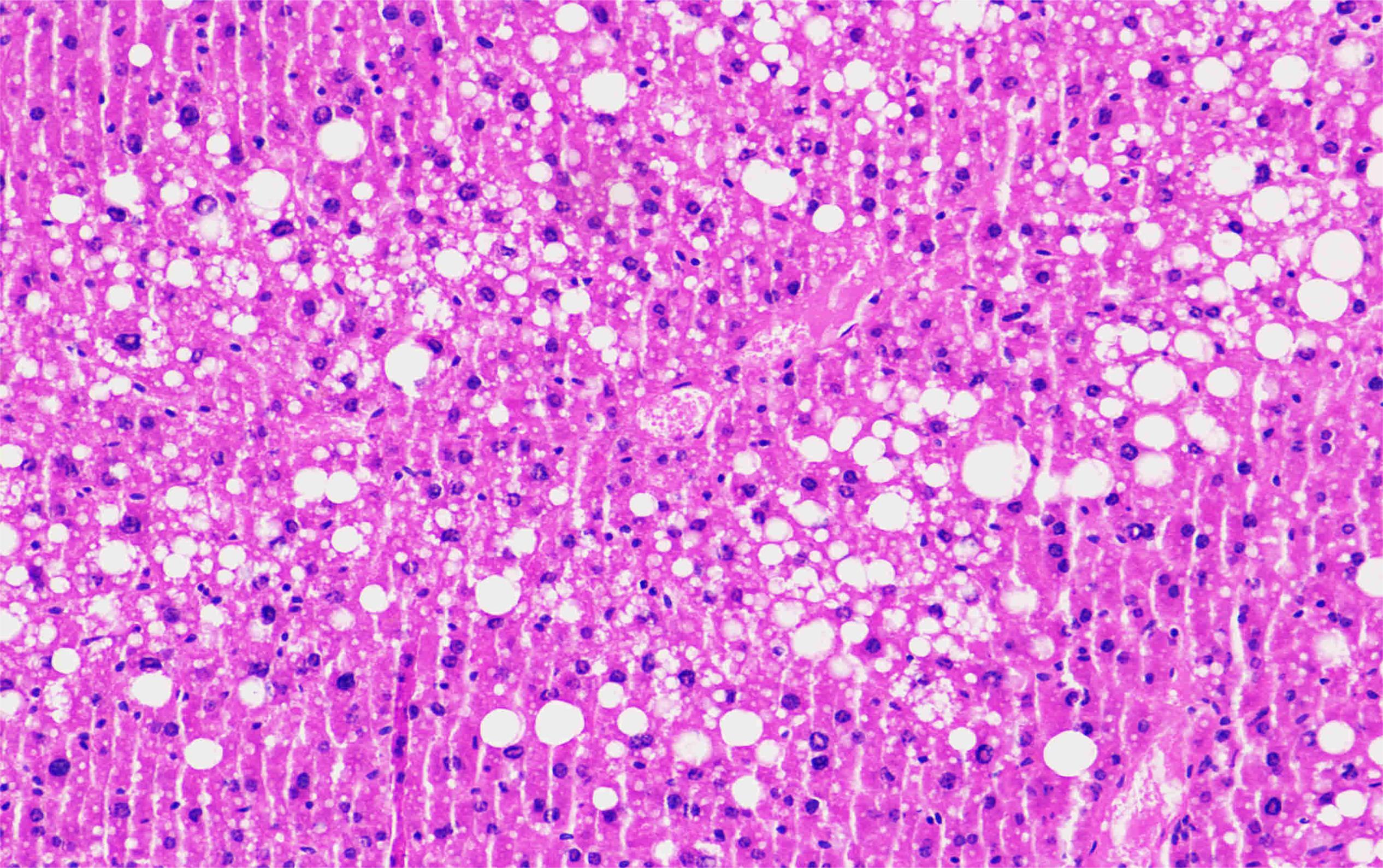

Supplement: Figure 4—source data 1. [file elife-85131-fig4-data1.zip › Figure 4-source data 1/Figure 4-raw microscopy images/H_E/CTRL.jpg]

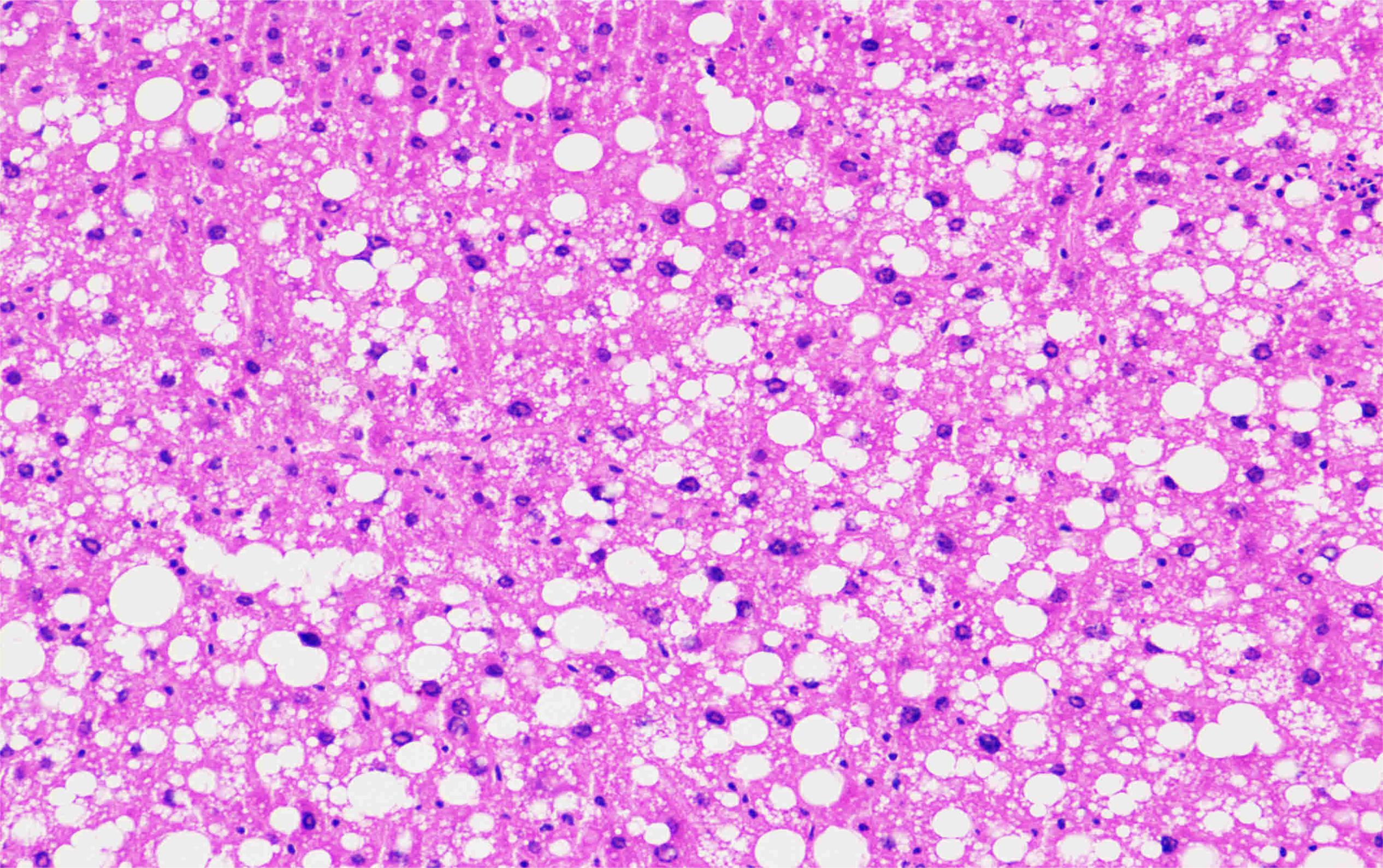

Supplement: Figure 4—source data 1. [file elife-85131-fig4-data1.zip › Figure 4-source data 1/Figure 4-raw microscopy images/H_E/ASO1.jpg]

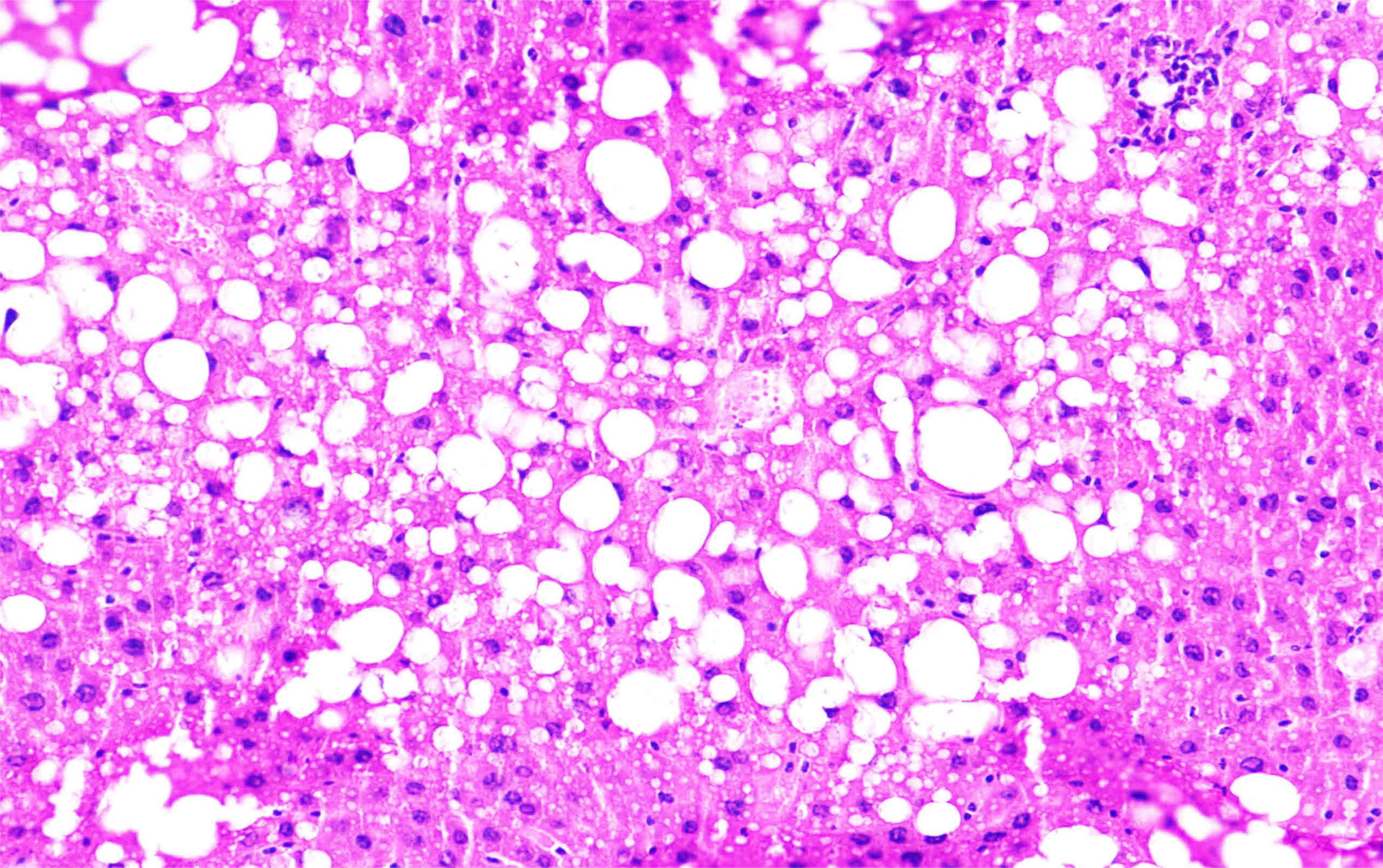

Supplement: Figure 4—source data 1. [file elife-85131-fig4-data1.zip › Figure 4-source data 1/Figure 4-raw microscopy images/H_E/AAV.jpg]

## Slide 1
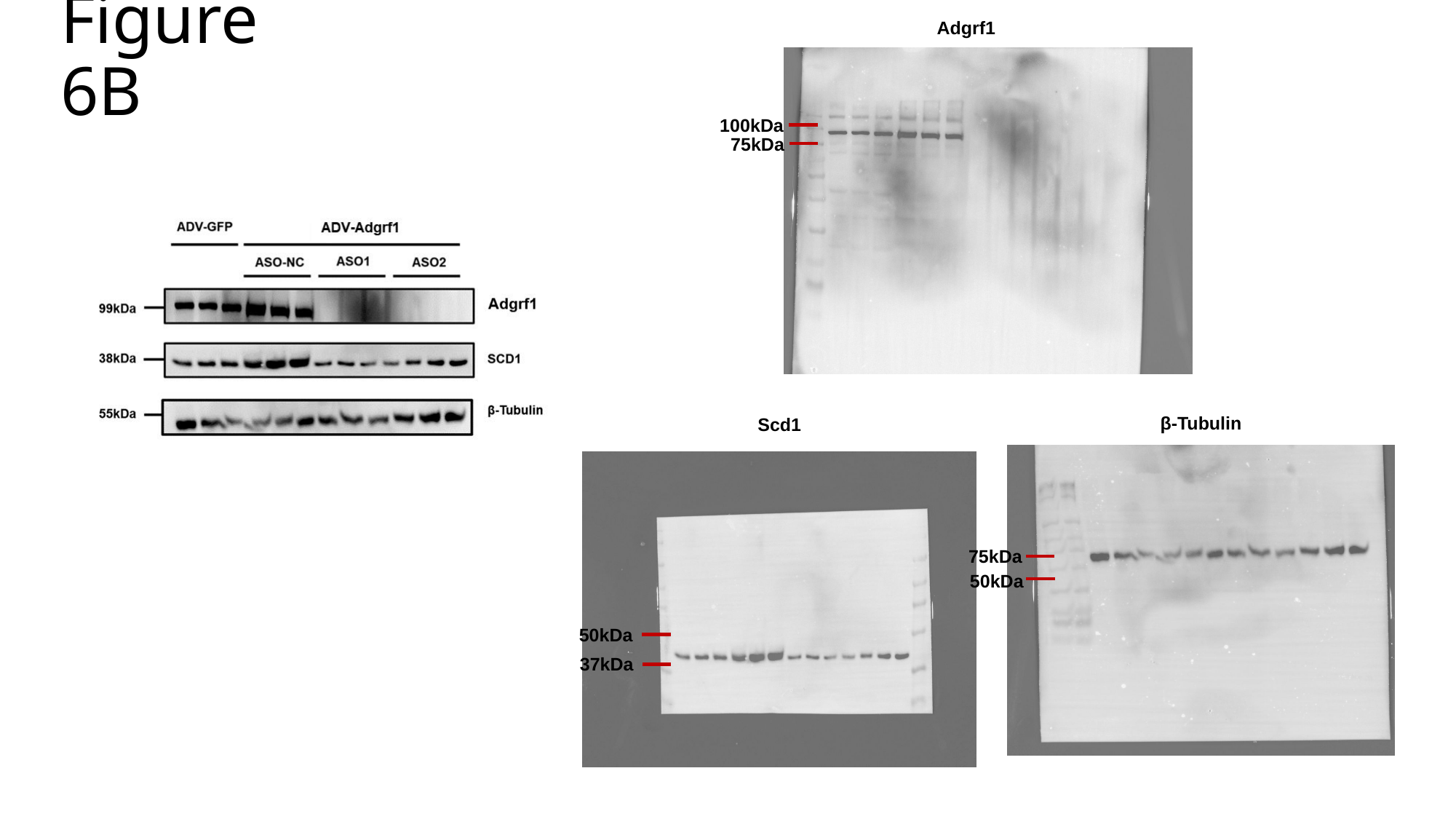

Adgrf1
100kDa
75kDa
# Figure 6B
β-Tubulin
75kDa
50kDa
Scd1
50kDa
37kDa

Supplement: Figure 6—source data 1. [file elife-85131-fig6-data1.zip › Figure 6-source data 1/Figure 6-Source 2.pptx]

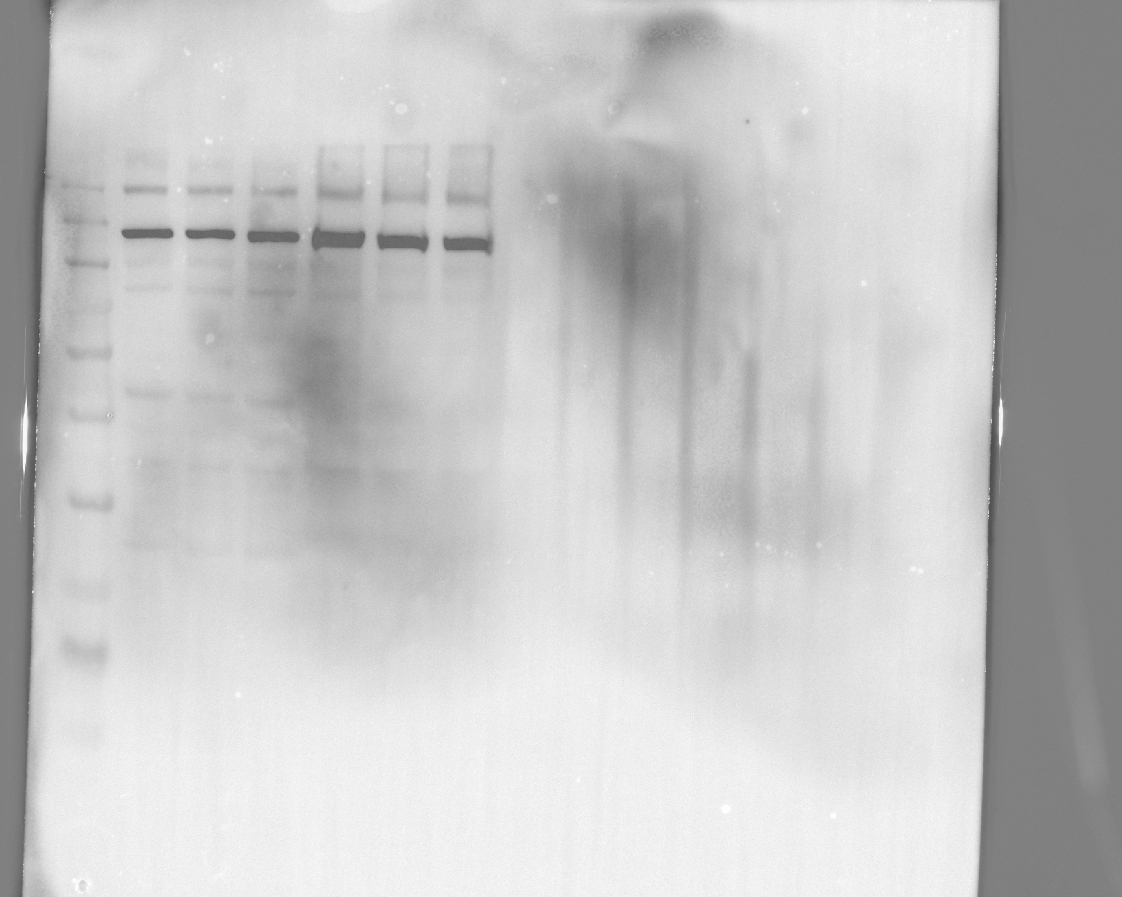

Supplement: Figure 6—source data 1. [file elife-85131-fig6-data1.zip › Figure 6-source data 1/Figure 6-raw gel image/Fig 6B Adgrf1.tif]

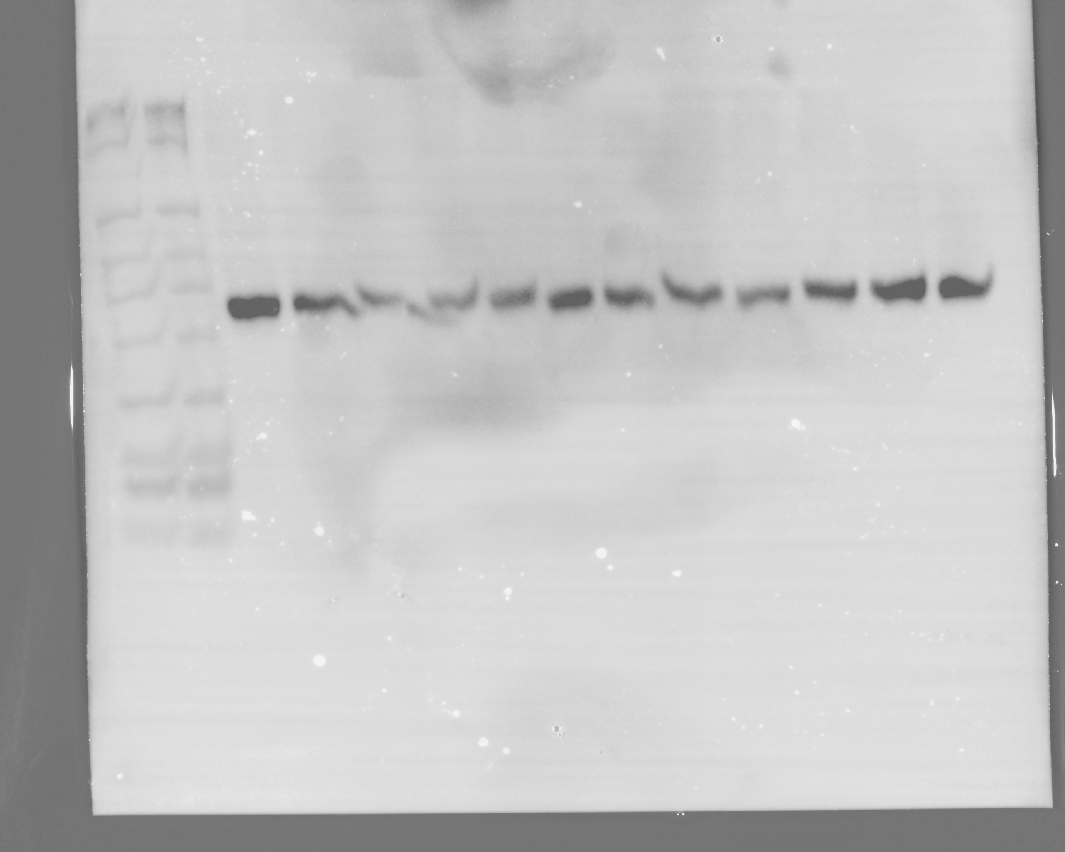

Supplement: Figure 6—source data 1. [file elife-85131-fig6-data1.zip › Figure 6-source data 1/Figure 6-raw gel image/Fig 6B b-tubulin.tif]

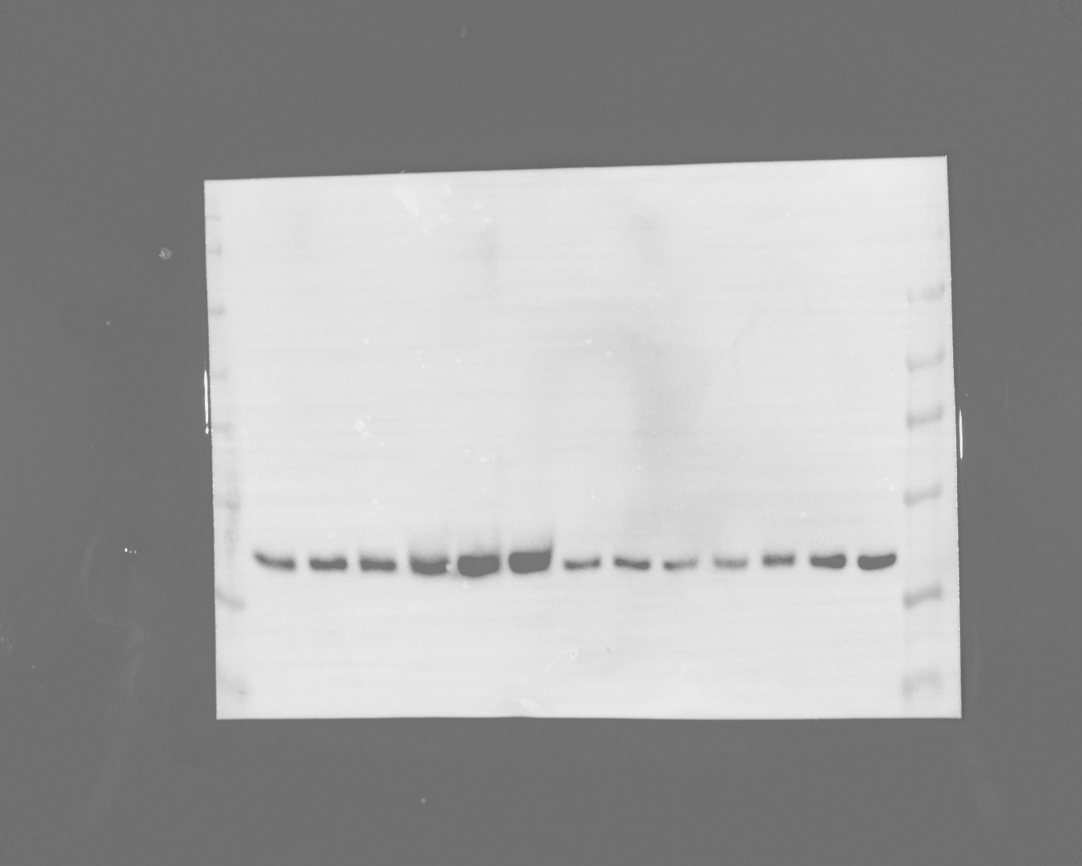

Supplement: Figure 6—source data 1. [file elife-85131-fig6-data1.zip › Figure 6-source data 1/Figure 6-raw gel image/Fig 6B Scd1.tif]

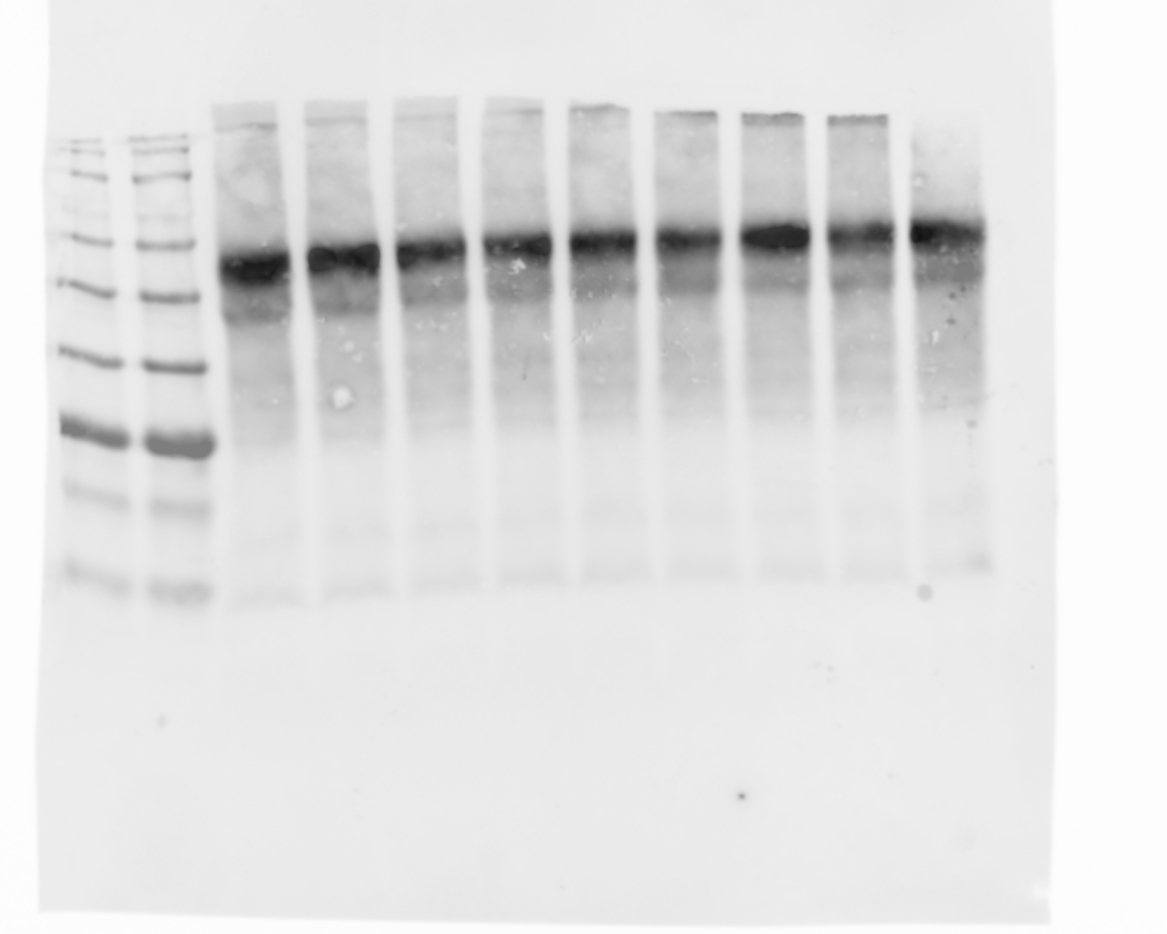

Supplement: Figure 7—source data 1. [file elife-85131-fig7-data1.zip › Figure 7-source data 1/Figure 7-raw gel image/Fig 7C b-tubulin.tif]

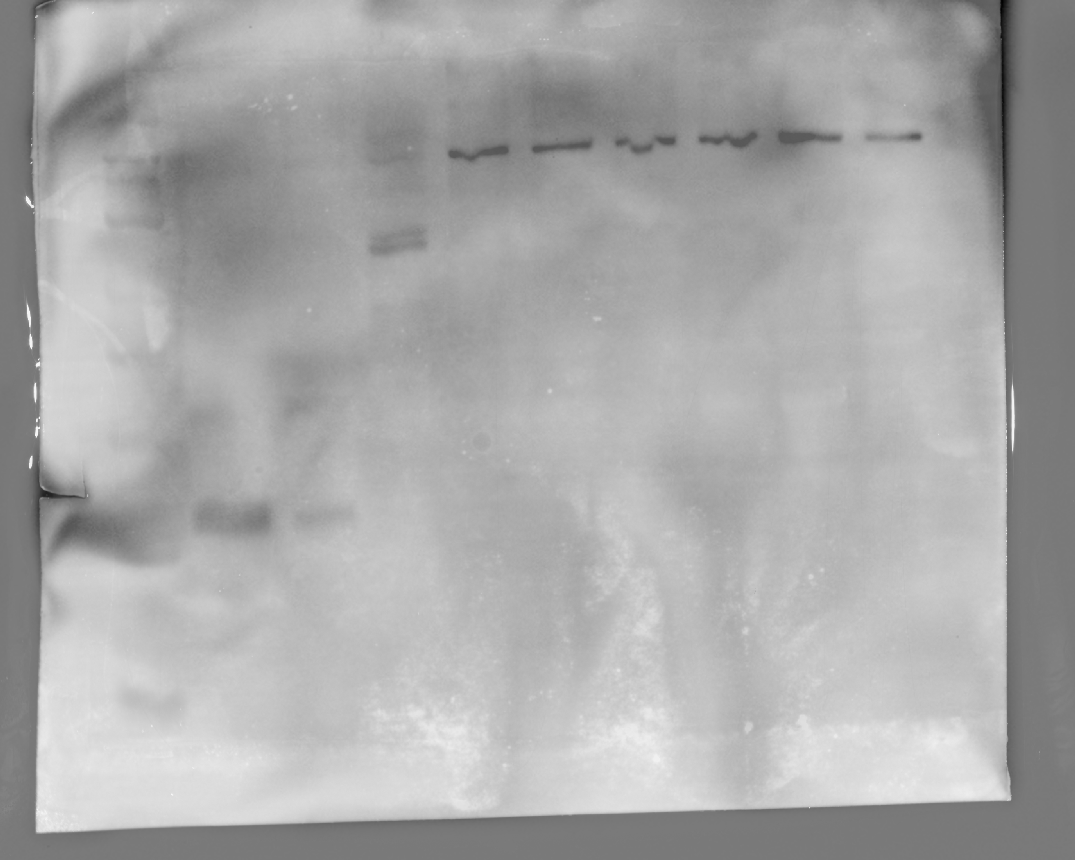

Supplement: Figure 7—source data 1. [file elife-85131-fig7-data1.zip › Figure 7-source data 1/Figure 7-raw gel image/Fig 7C Adgrf1.tif]

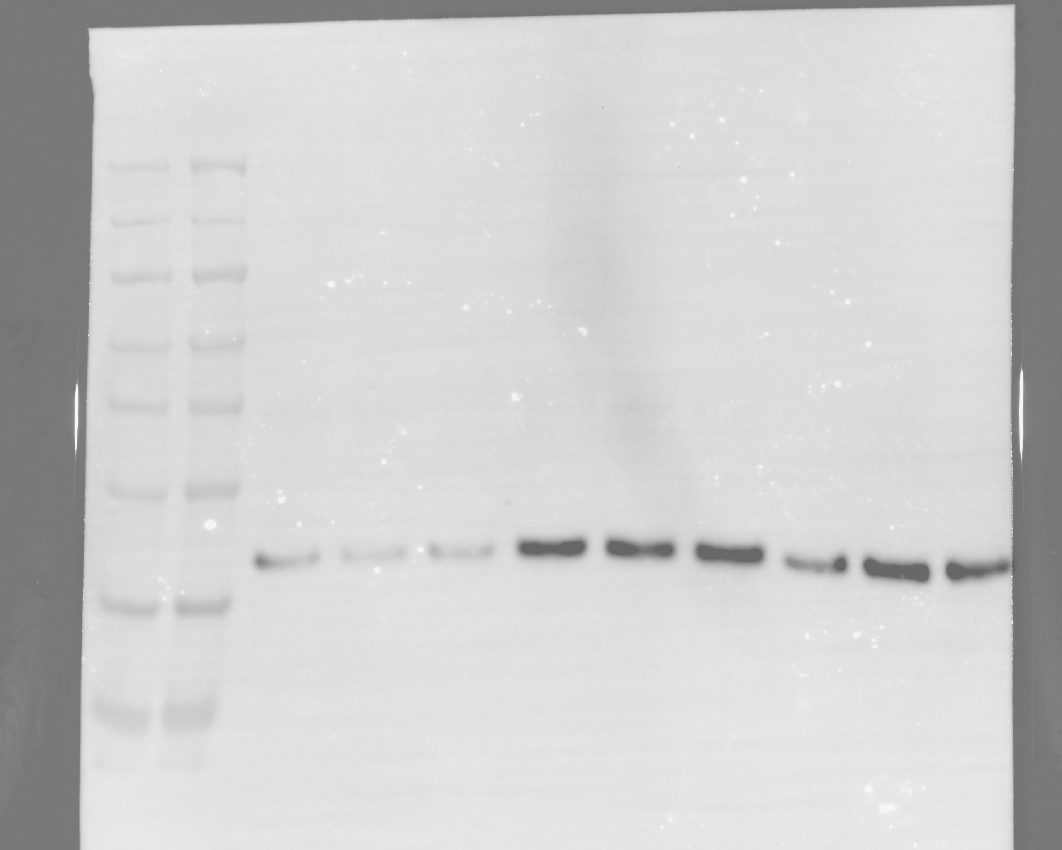

Supplement: Figure 7—source data 1. [file elife-85131-fig7-data1.zip › Figure 7-source data 1/Figure 7-raw gel image/Fig 7C Scd1.tif]

## Slide 1
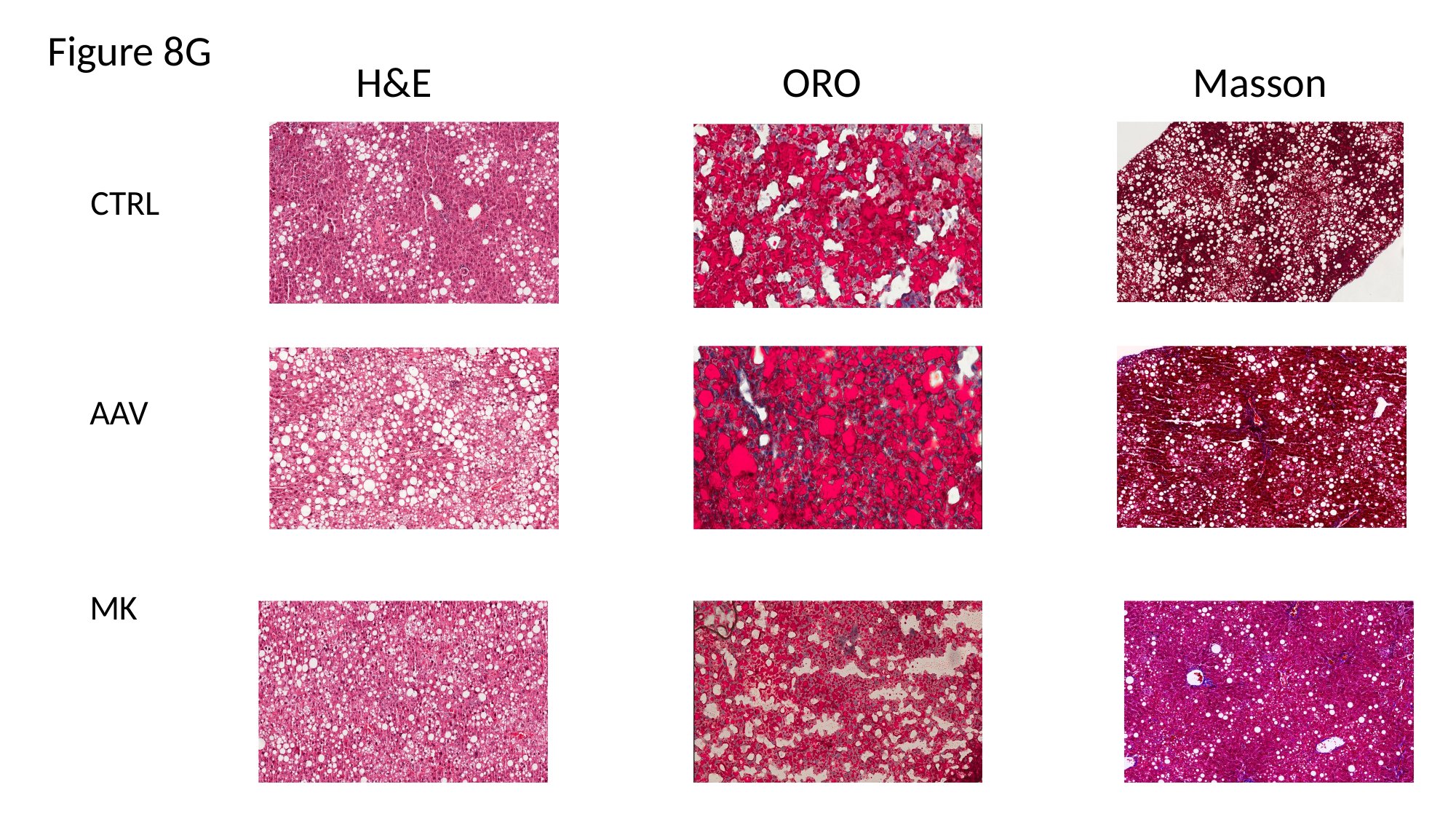

Figure 8G
Masson
H&E
ORO
CTRL
AAV
MK

Supplement: Figure 8—source data 1. [file elife-85131-fig8-data1.zip › Figure 8-source data 1/Figure 8-Source 2.pptx]

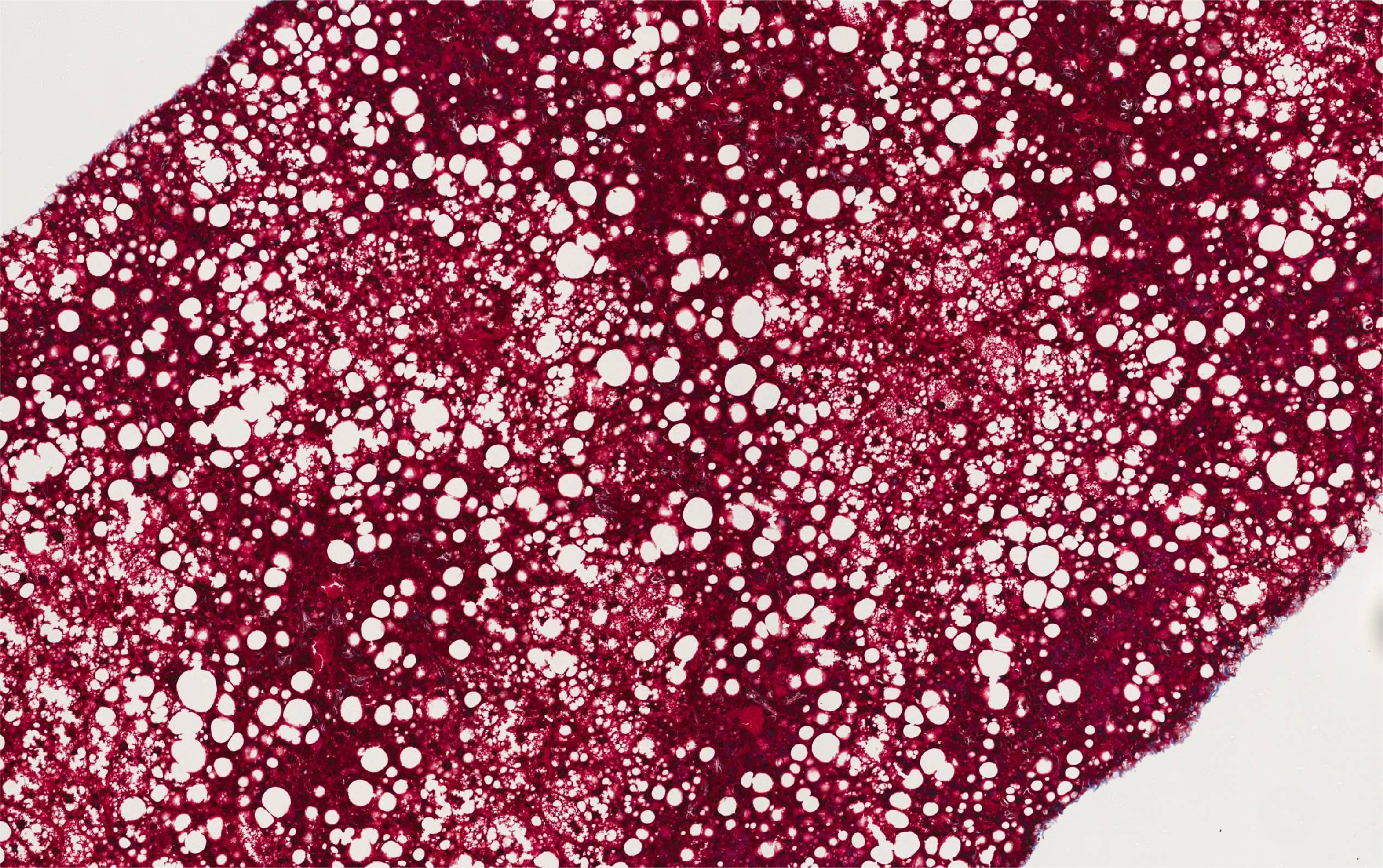

Supplement: Figure 8—source data 1. [file elife-85131-fig8-data1.zip › Figure 8-source data 1/Figure 8-raw microscopy images/Masson/CTRL.jpg]

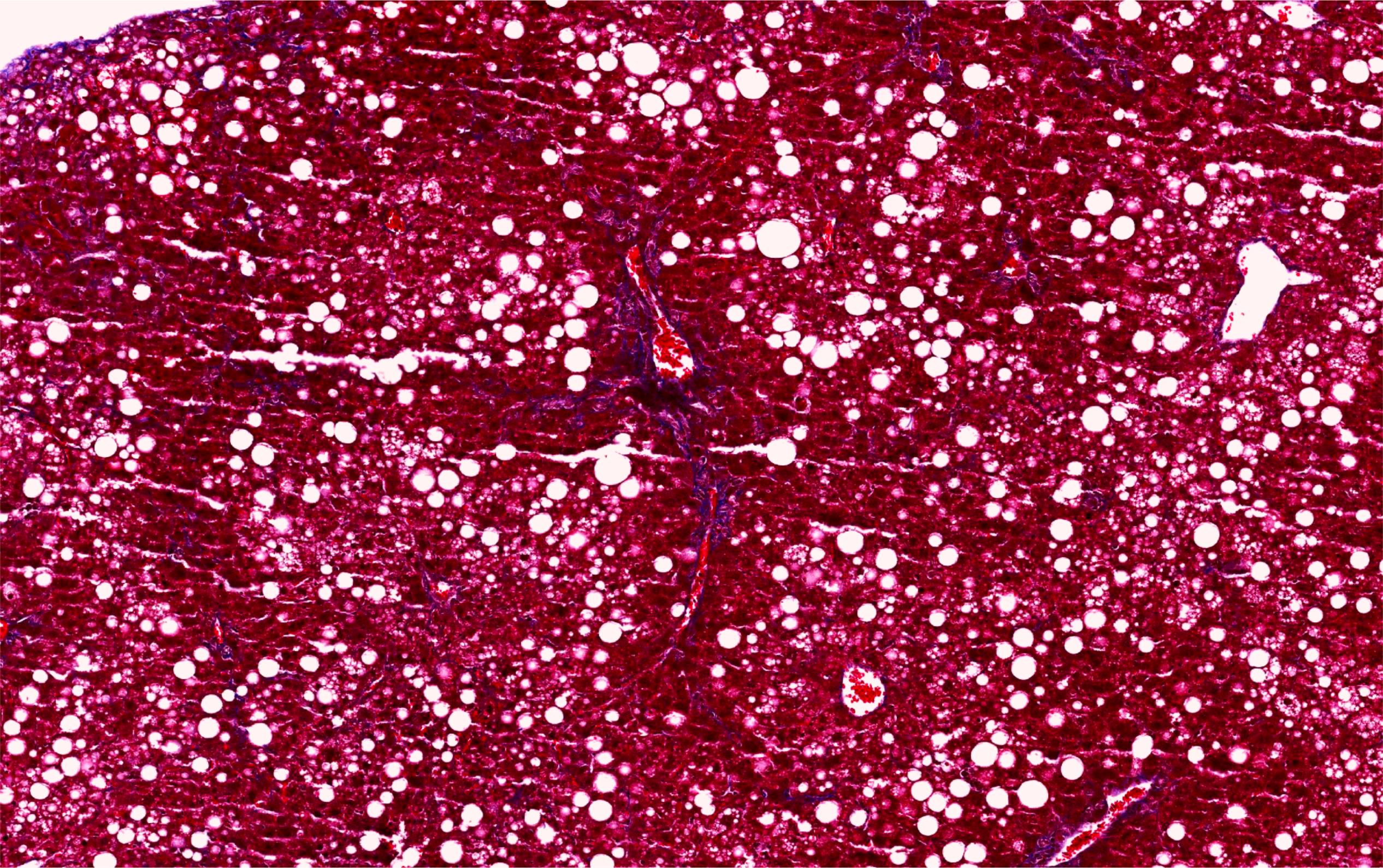

Supplement: Figure 8—source data 1. [file elife-85131-fig8-data1.zip › Figure 8-source data 1/Figure 8-raw microscopy images/Masson/AAV.jpg]

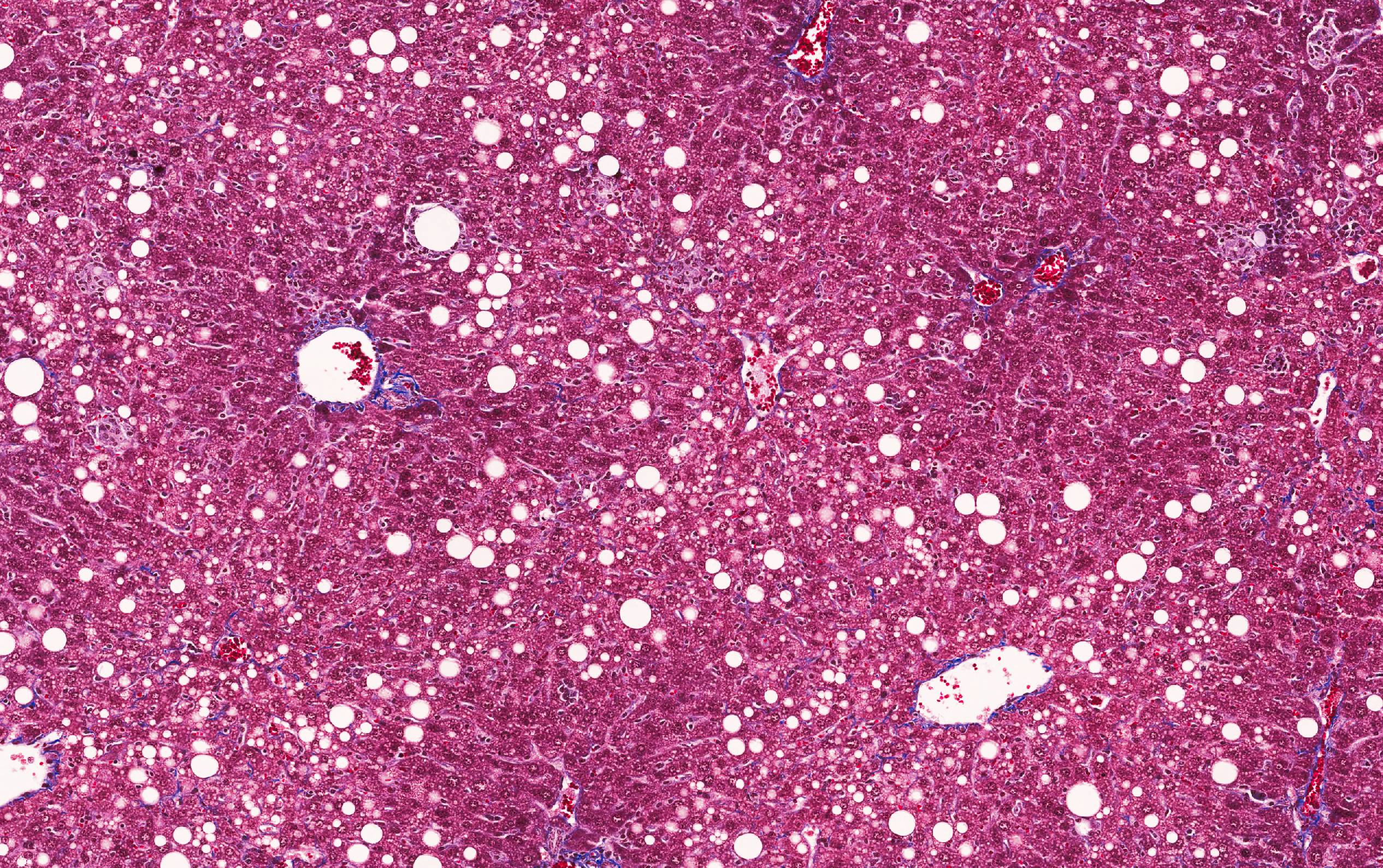

Supplement: Figure 8—source data 1. [file elife-85131-fig8-data1.zip › Figure 8-source data 1/Figure 8-raw microscopy images/Masson/MK.jpg]

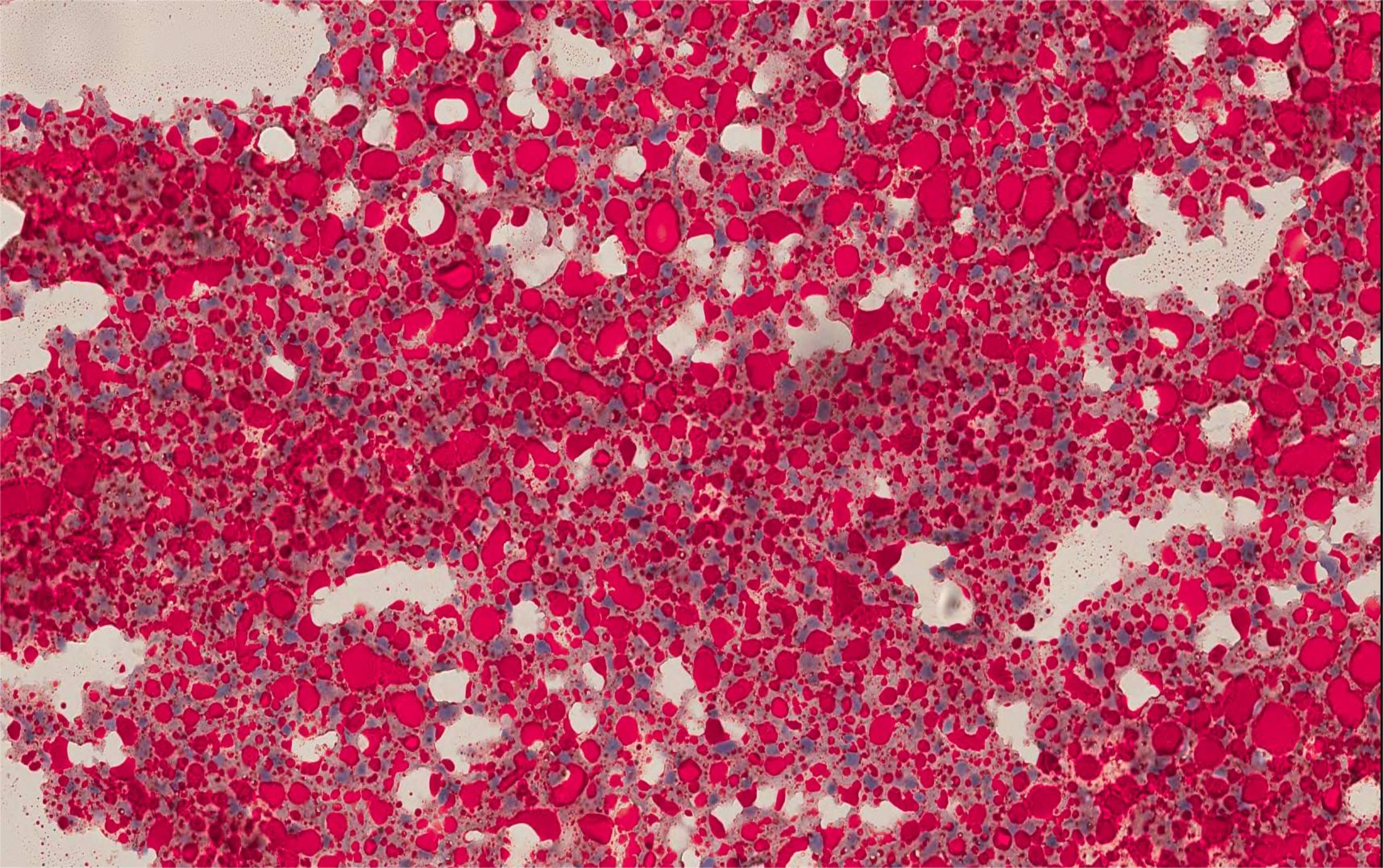

Supplement: Figure 8—source data 1. [file elife-85131-fig8-data1.zip › Figure 8-source data 1/Figure 8-raw microscopy images/Oil Red O/CTRL.jpg]

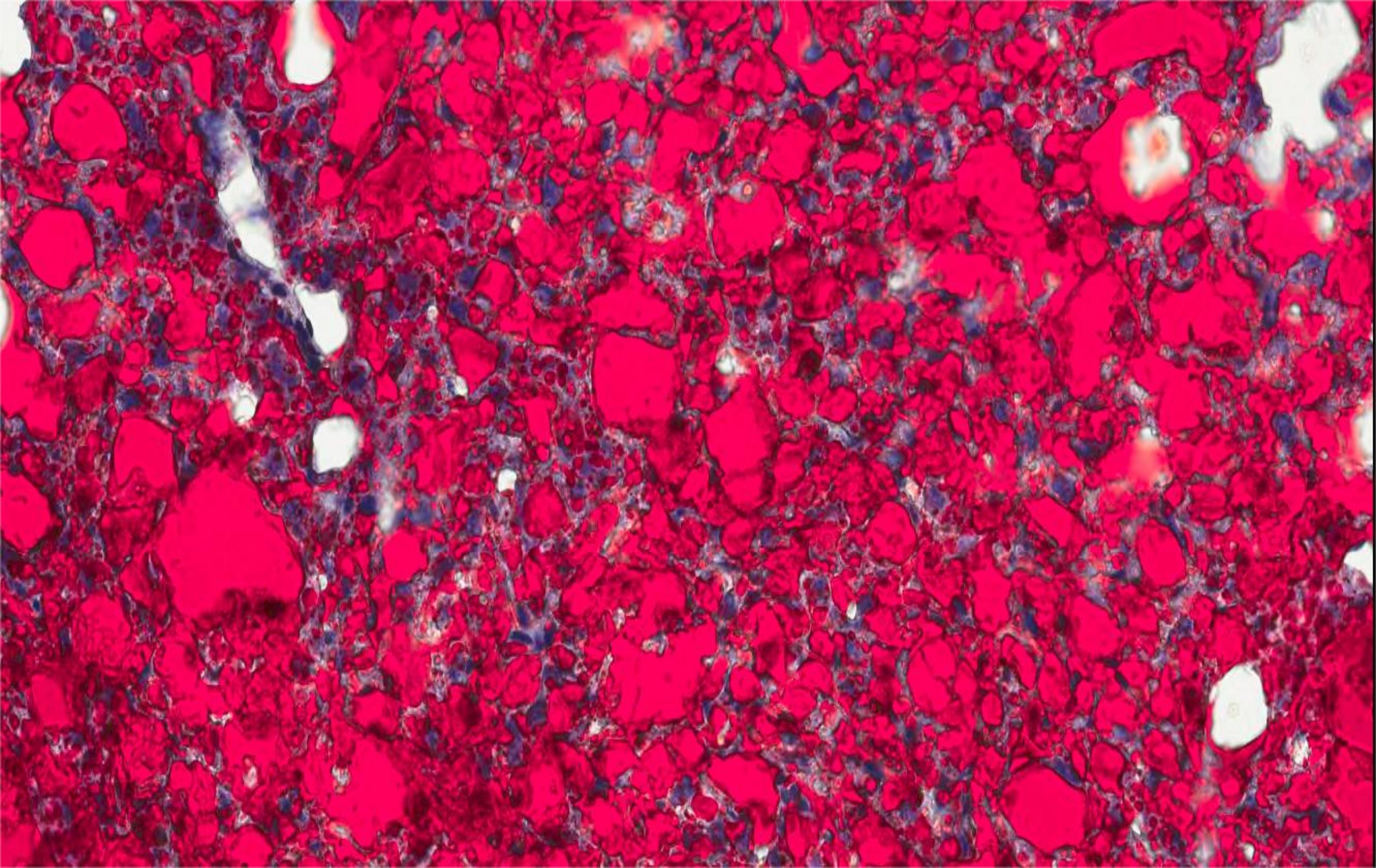

Supplement: Figure 8—source data 1. [file elife-85131-fig8-data1.zip › Figure 8-source data 1/Figure 8-raw microscopy images/Oil Red O/AAV.jpg]

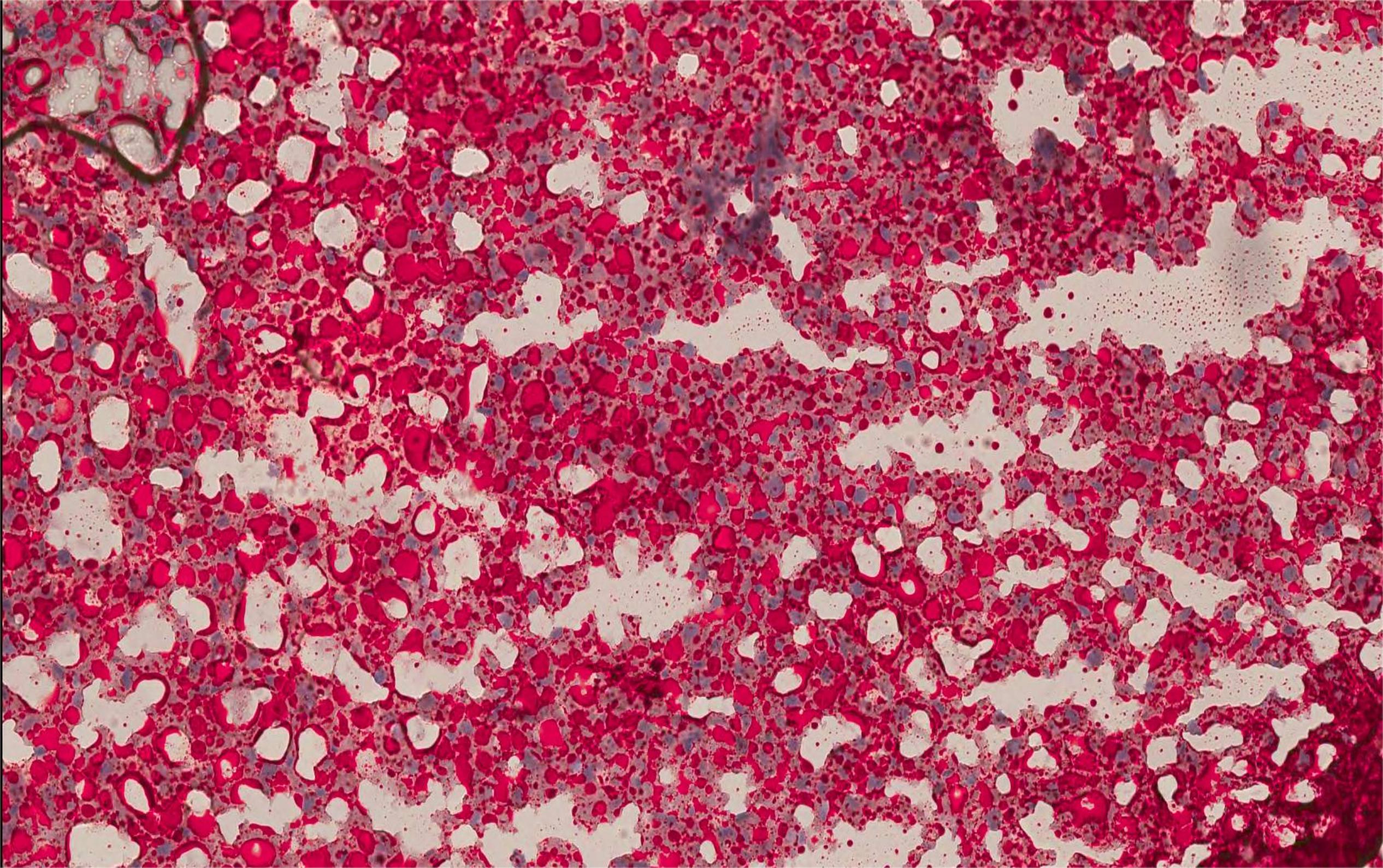

Supplement: Figure 8—source data 1. [file elife-85131-fig8-data1.zip › Figure 8-source data 1/Figure 8-raw microscopy images/Oil Red O/MK.jpg]

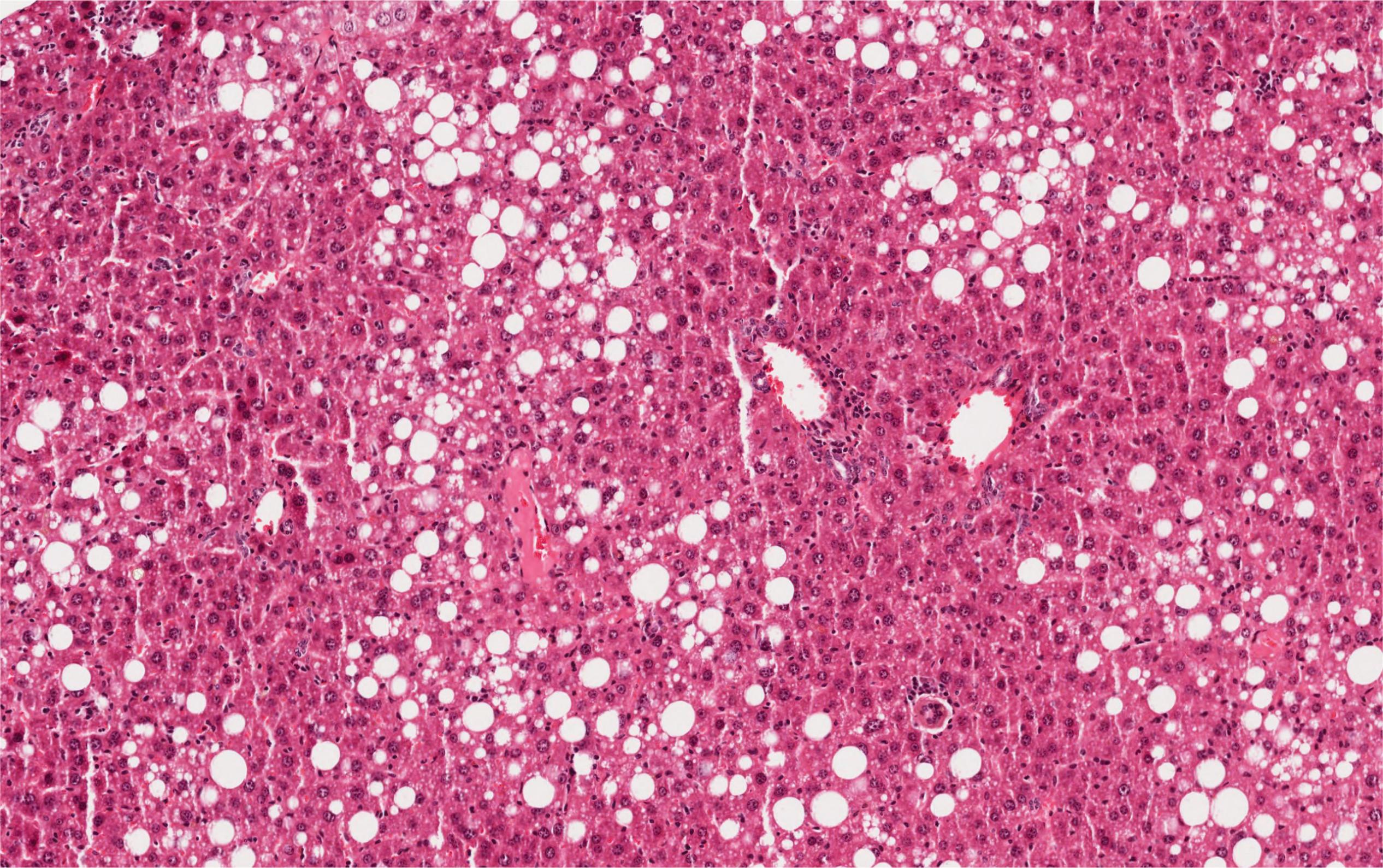

Supplement: Figure 8—source data 1. [file elife-85131-fig8-data1.zip › Figure 8-source data 1/Figure 8-raw microscopy images/H_E/CTRL.jpg]

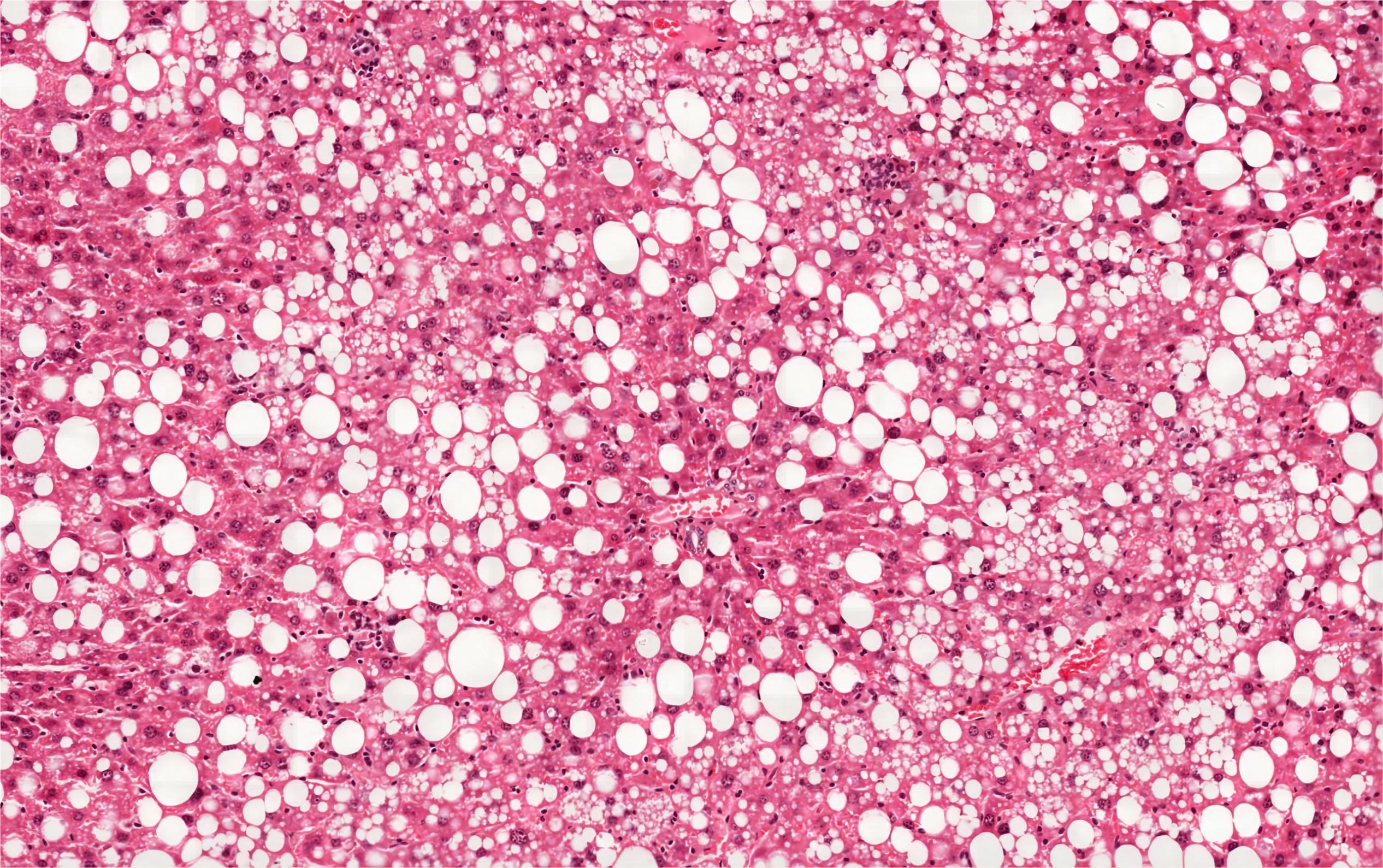

Supplement: Figure 8—source data 1. [file elife-85131-fig8-data1.zip › Figure 8-source data 1/Figure 8-raw microscopy images/H_E/AAV.jpg]

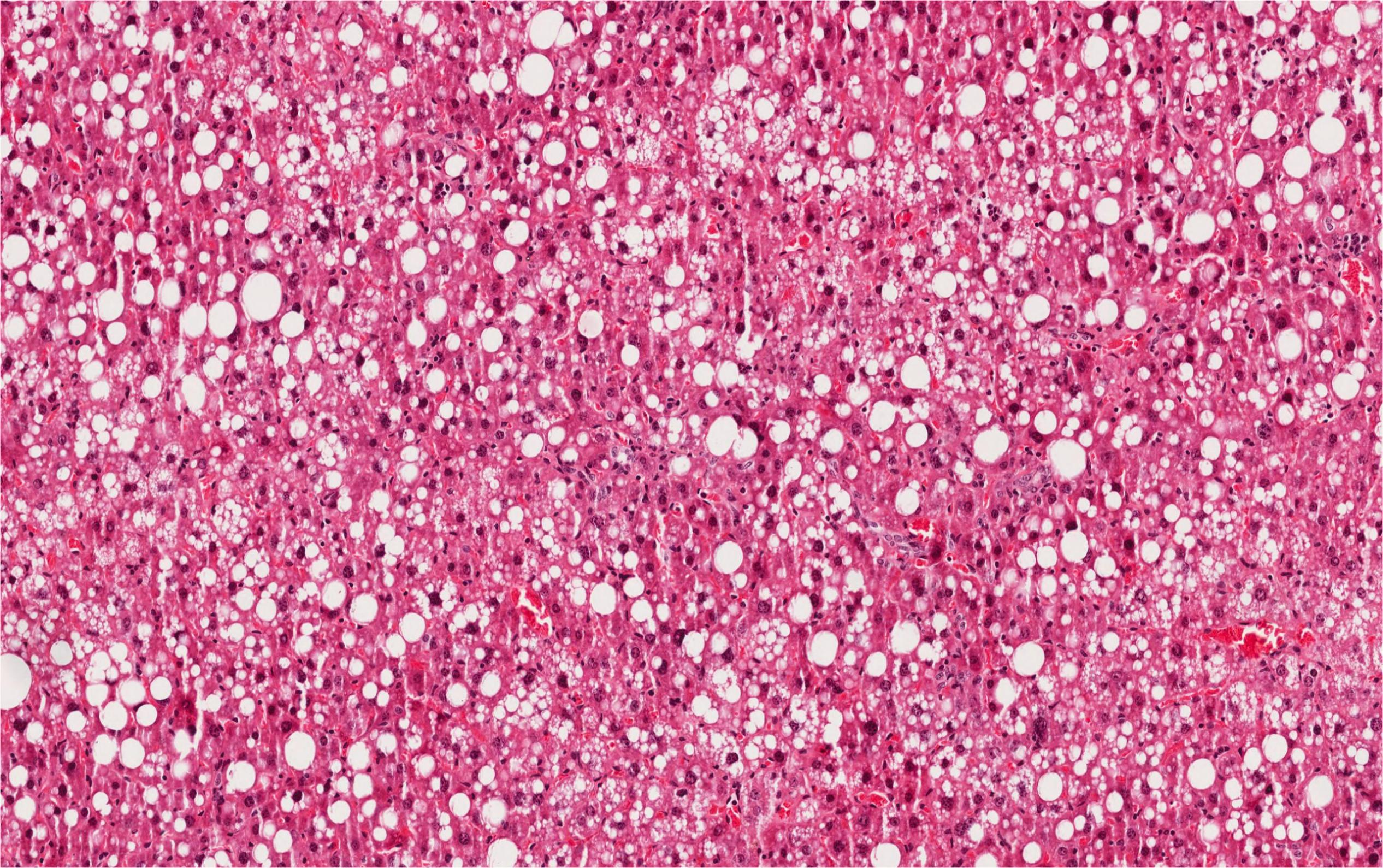

Supplement: Figure 8—source data 1. [file elife-85131-fig8-data1.zip › Figure 8-source data 1/Figure 8-raw microscopy images/H_E/MK.jpg]

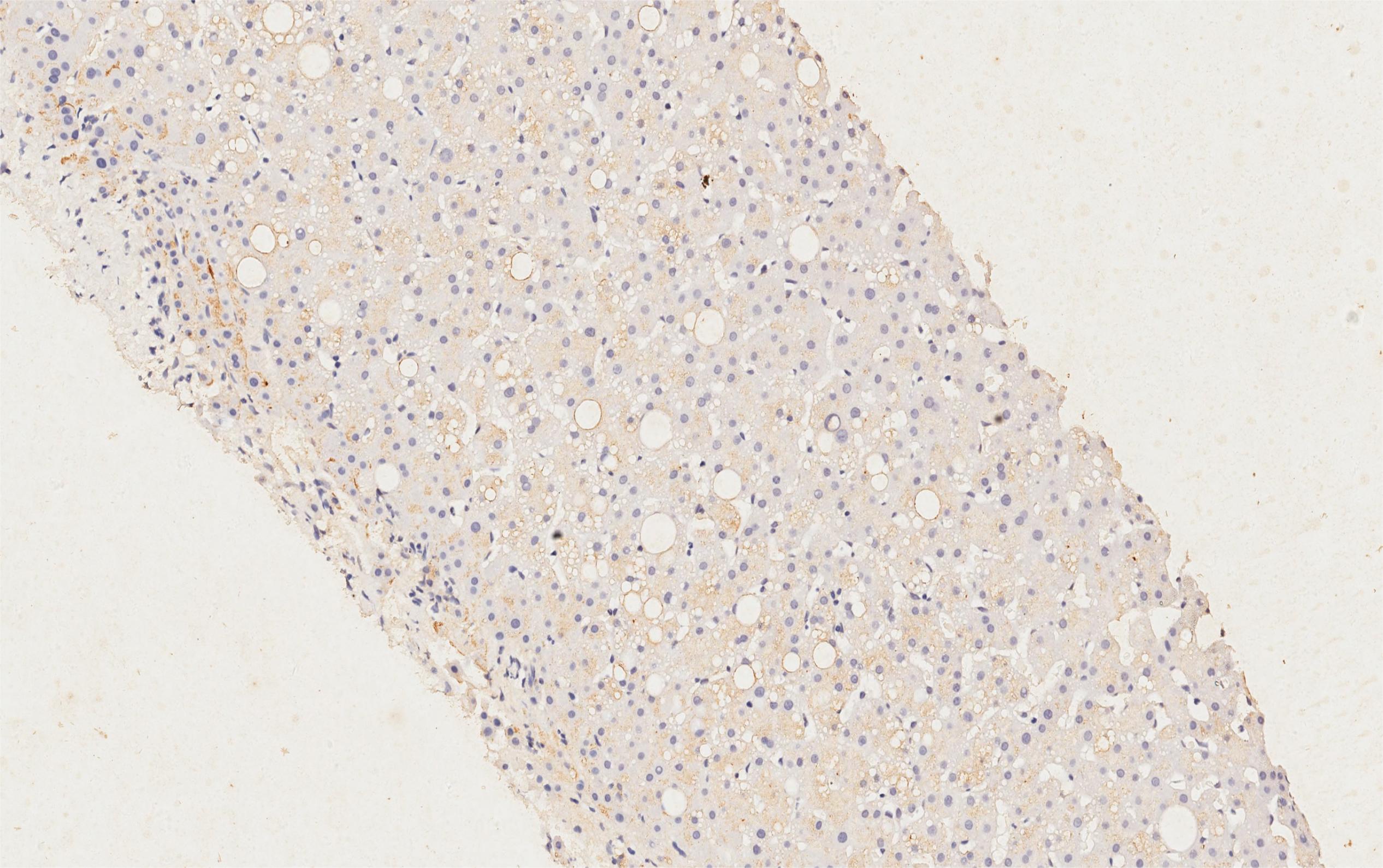

Supplement: Figure 9—source data 1. [file elife-85131-fig9-data1.zip › Figure 9-source data 1/Figure 9-raw microscopy images/IHC/Moderate.jpg]

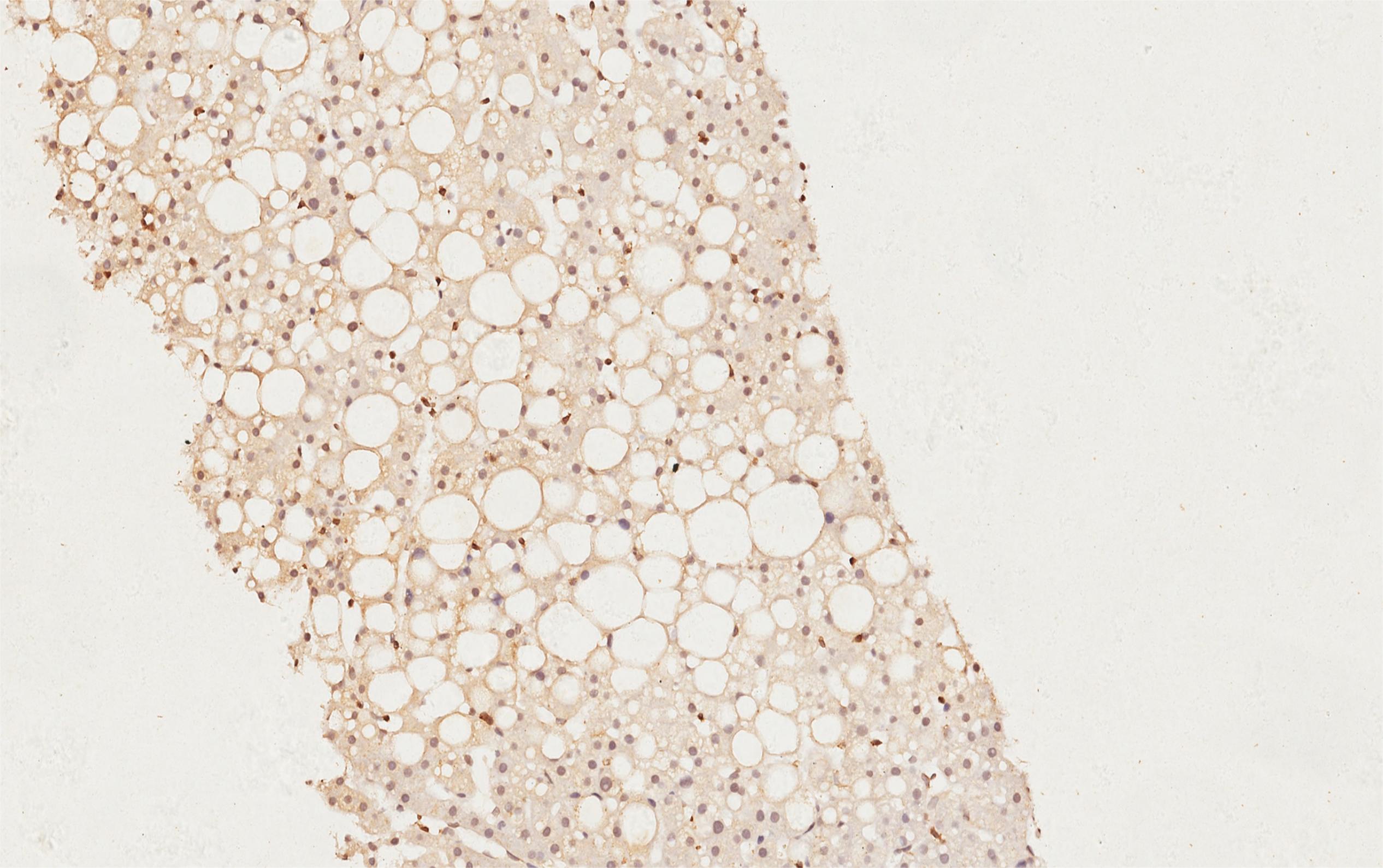

Supplement: Figure 9—source data 1. [file elife-85131-fig9-data1.zip › Figure 9-source data 1/Figure 9-raw microscopy images/IHC/Severe.jpg]

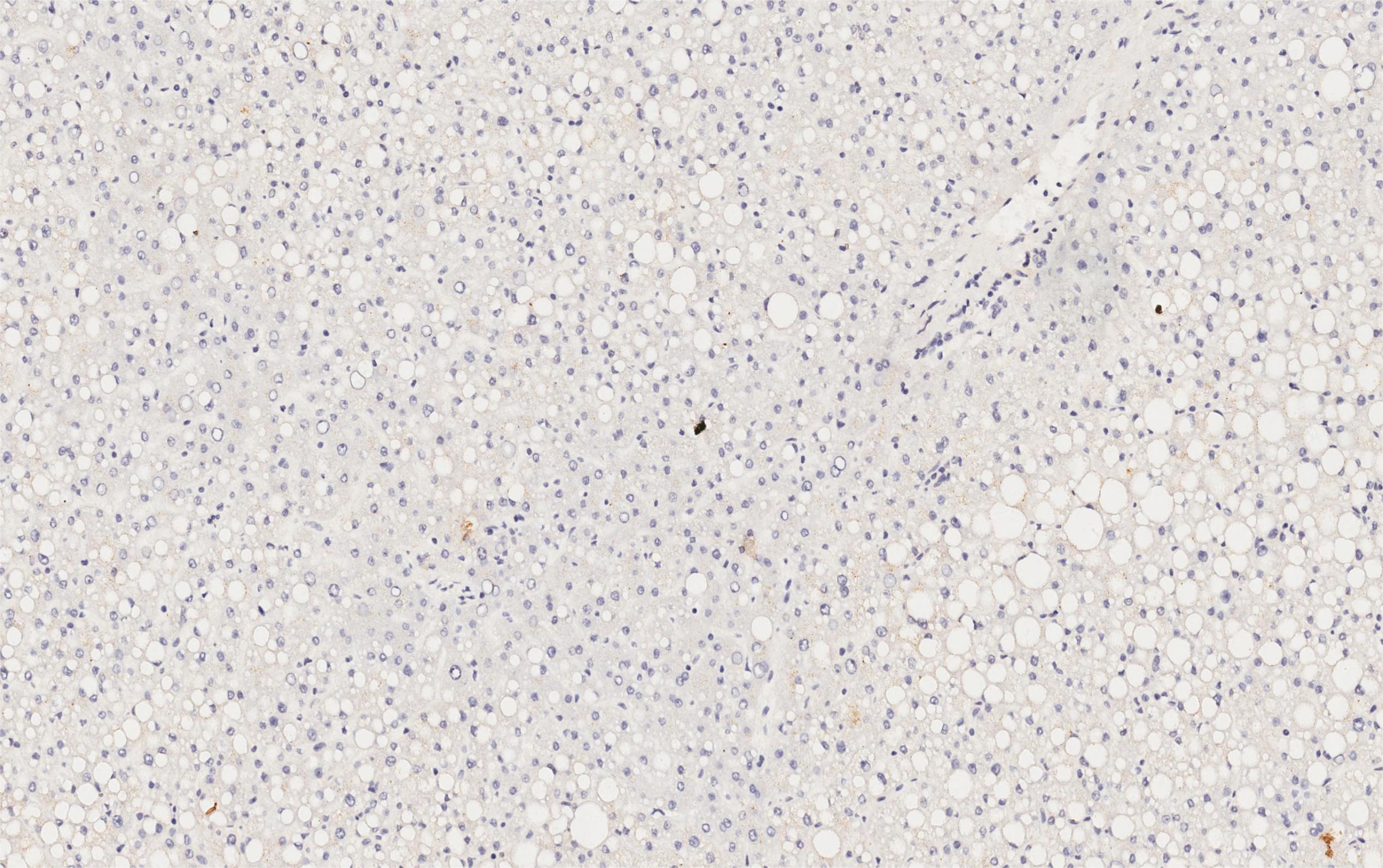

Supplement: Figure 9—source data 1. [file elife-85131-fig9-data1.zip › Figure 9-source data 1/Figure 9-raw microscopy images/IHC/Mild.jpg]

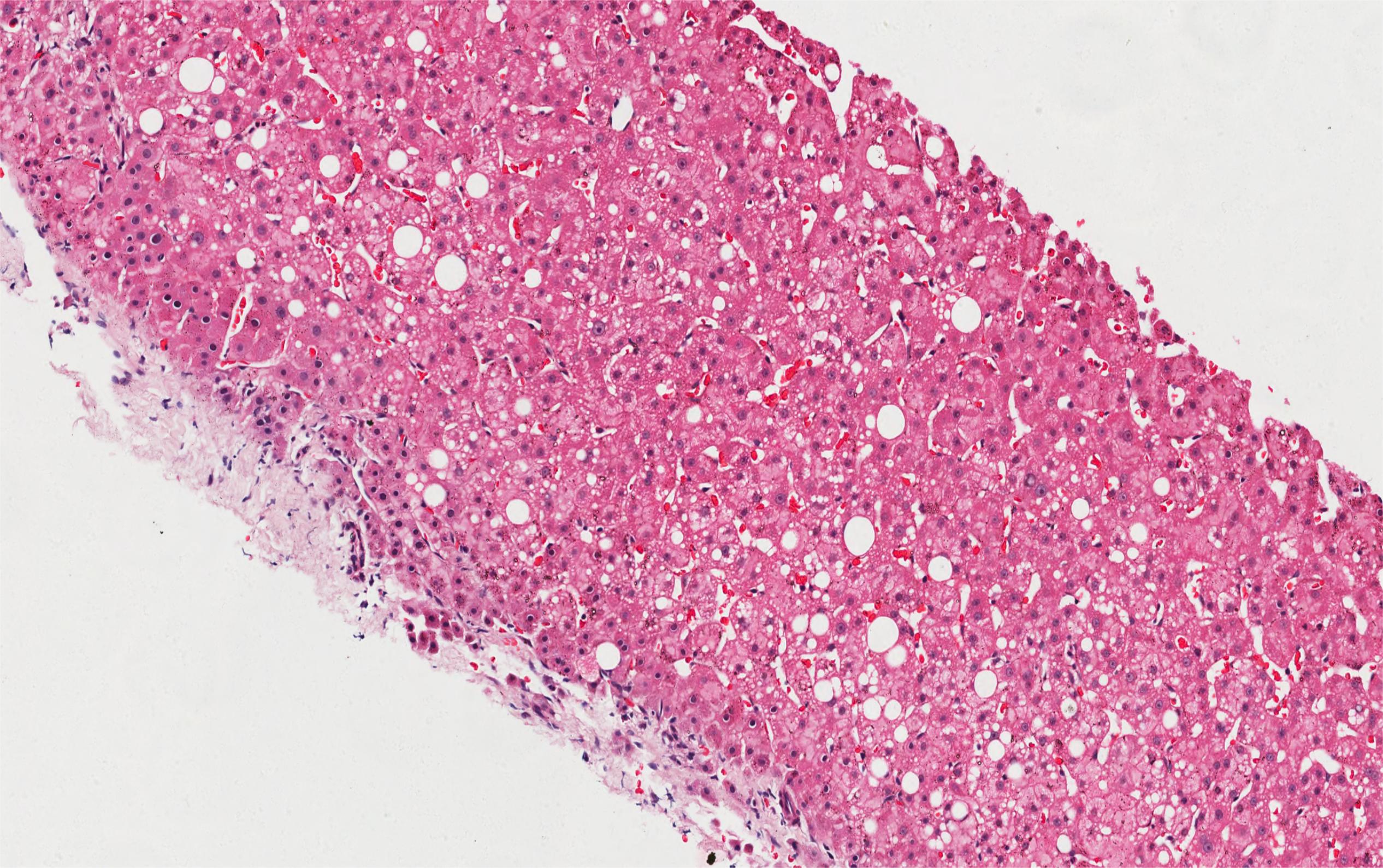

Supplement: Figure 9—source data 1. [file elife-85131-fig9-data1.zip › Figure 9-source data 1/Figure 9-raw microscopy images/H_E/Moderate.jpg]

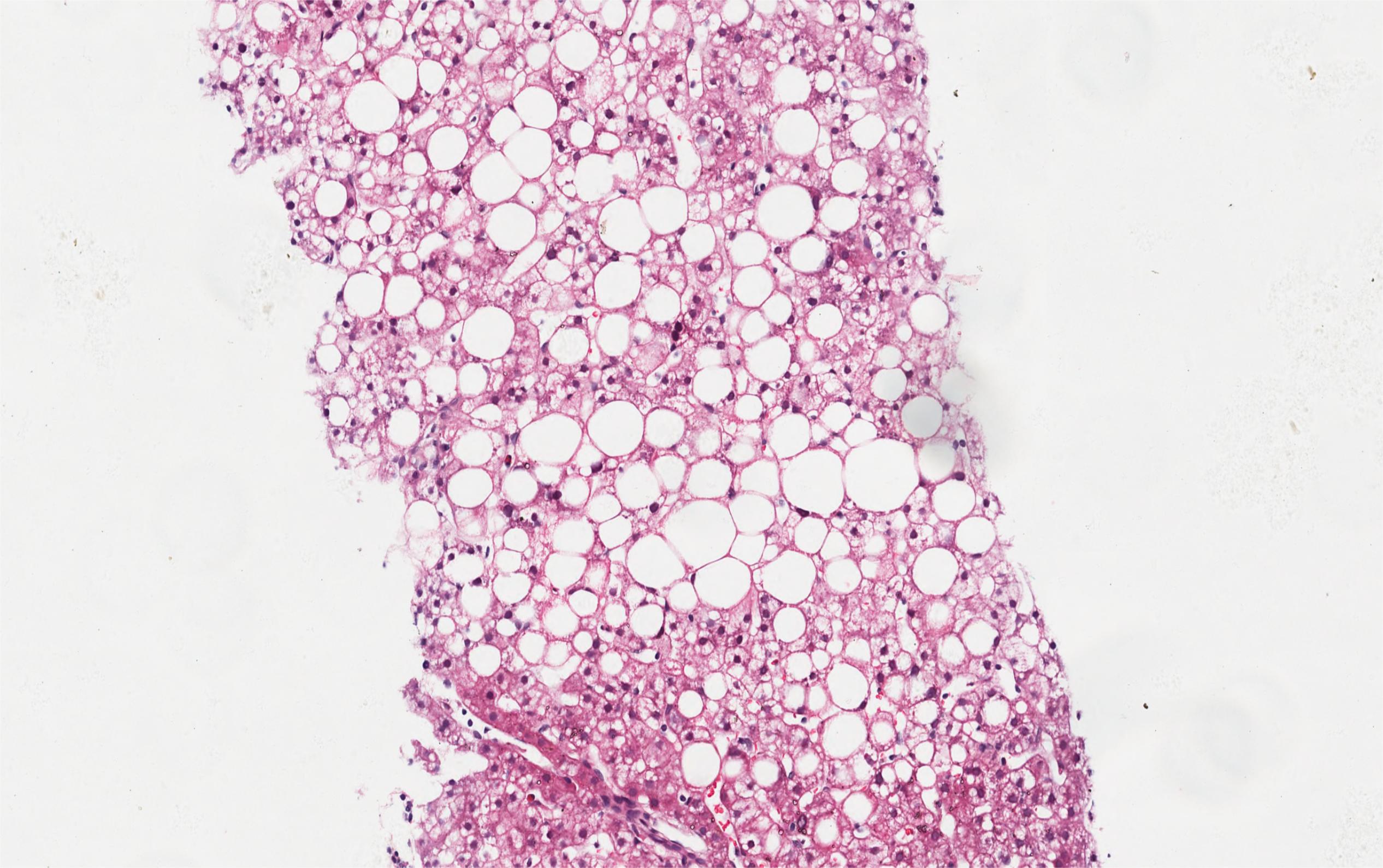

Supplement: Figure 9—source data 1. [file elife-85131-fig9-data1.zip › Figure 9-source data 1/Figure 9-raw microscopy images/H_E/Severe.jpg]

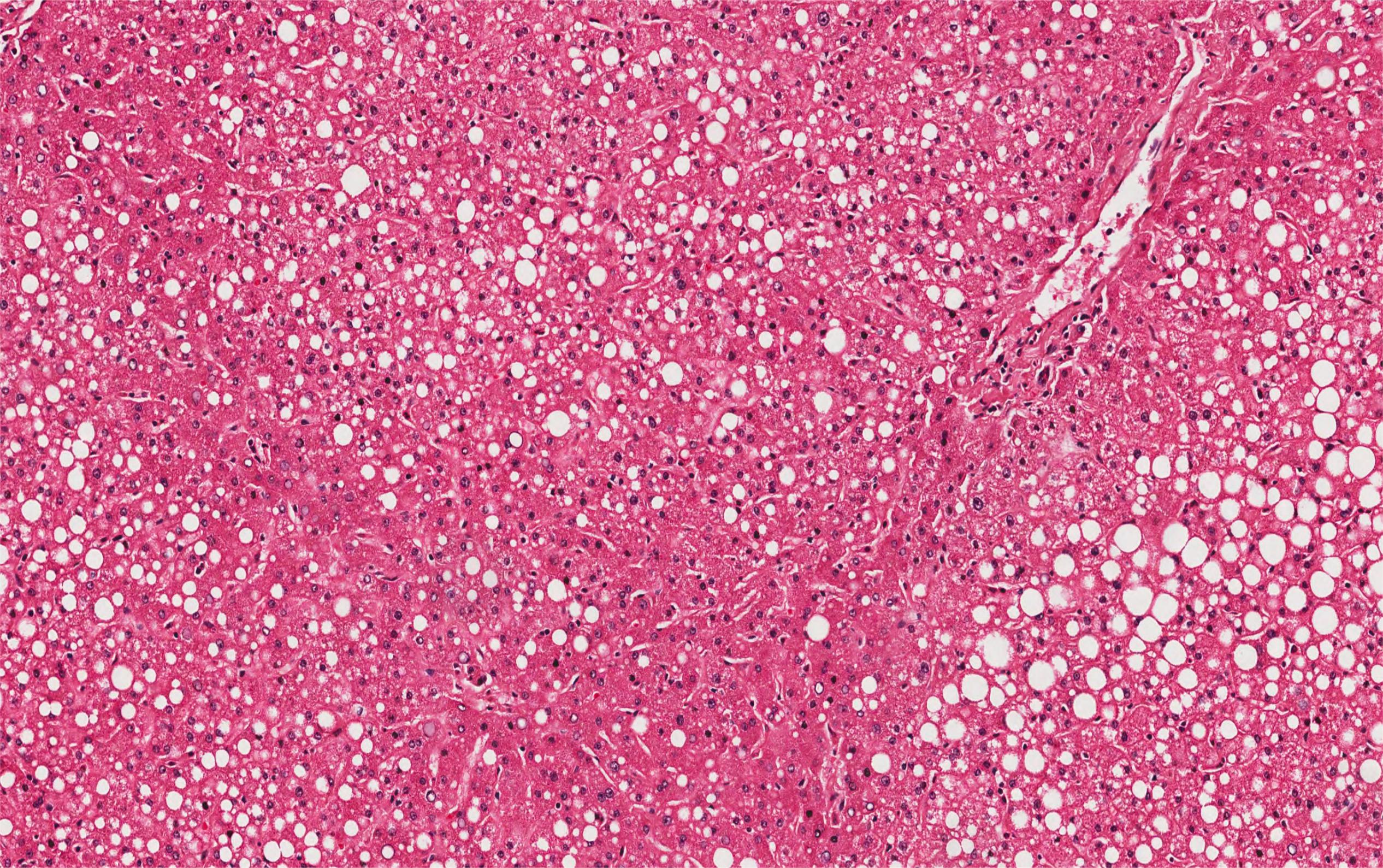

Supplement: Figure 9—source data 1. [file elife-85131-fig9-data1.zip › Figure 9-source data 1/Figure 9-raw microscopy images/H_E/Mild.jpg]
