# Supplementary material for: Amelioration of non-alcoholic fatty liver disease by targeting adhesion G protein-coupled receptor F1 (Adgrf1)
Source: eLife. 2023 Aug 15;12:e85131. doi: 10.7554/eLife.85131 (PMC10427146; doi:10.7554/eLife.85131)
Supplement: Figure 2—source data 1. [file elife-85131-fig2-data1.zip › Figure 2-source data 1/Figure 2-Source 2-WB.pptx]

## Slide 1
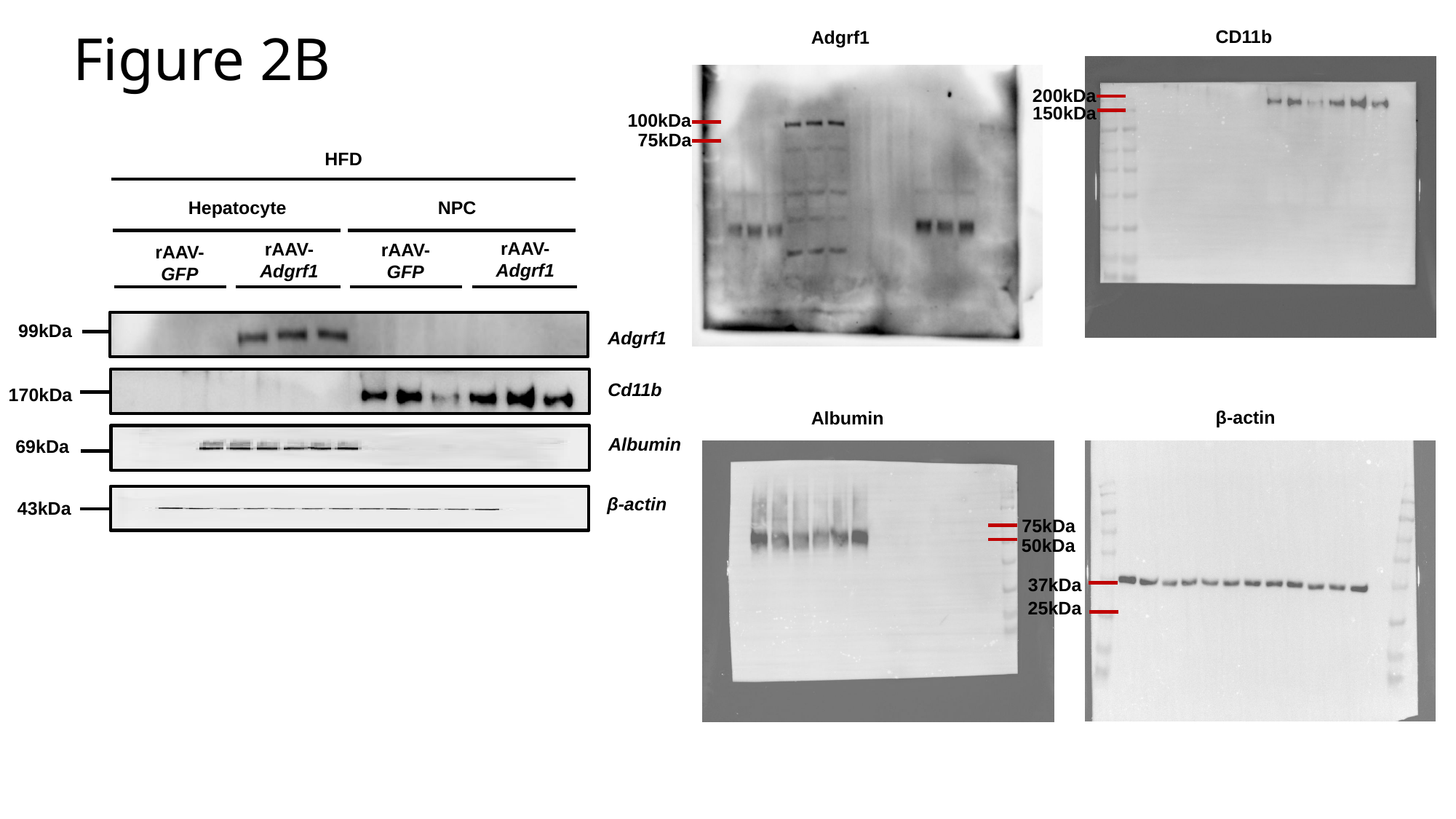

CD11b
Adgrf1
# Figure 2B
200kDa
150kDa
100kDa
75kDa
HFD
Hepatocyte
NPC
rAAV- Adgrf1
rAAV- Adgrf1
rAAV-
GFP
rAAV-
GFP
99kDa
Adgrf1
Cd11b
170kDa
Albumin
69kDa
β-actin
43kDa
β-actin
Albumin
75kDa
50kDa
37kDa
25kDa
