# Supplementary material for: Amelioration of non-alcoholic fatty liver disease by targeting adhesion G protein-coupled receptor F1 (Adgrf1)
Source: eLife. 2023 Aug 15;12:e85131. doi: 10.7554/eLife.85131 (PMC10427146; doi:10.7554/eLife.85131)
Supplement: Figure 2—figure supplement 1—source data 1. [file elife-85131-fig2-figsupp1-data1.zip › Figure 2-figure supplement 1-source data 1/Supplementary Figure 1-Source 2.pptx]

## Slide 1
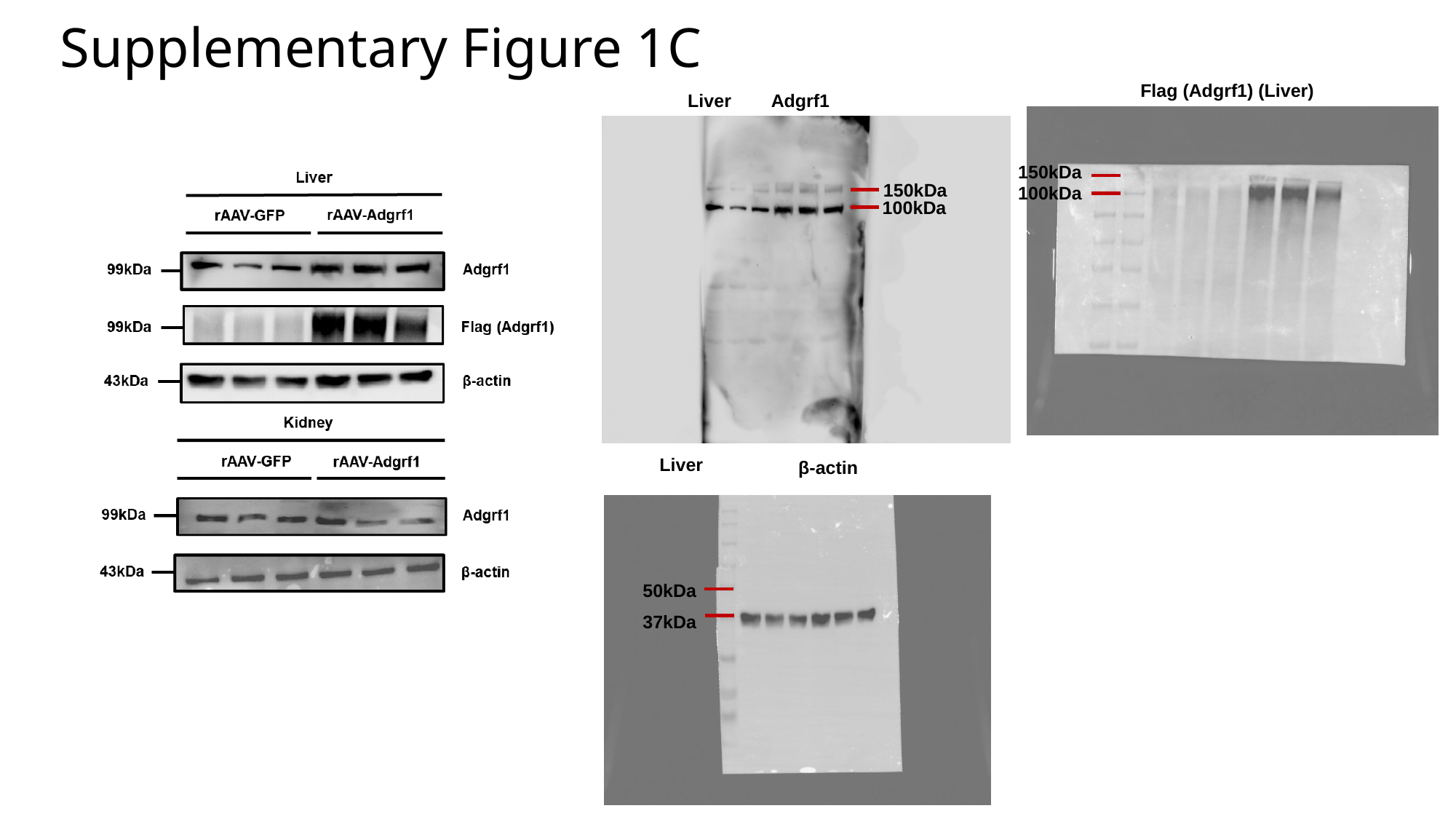

# Supplementary Figure 1C
Flag (Adgrf1) (Liver)
150kDa
100kDa
Liver
Adgrf1
150kDa
100kDa
Liver
50kDa
37kDa
β-actin

## Slide 2
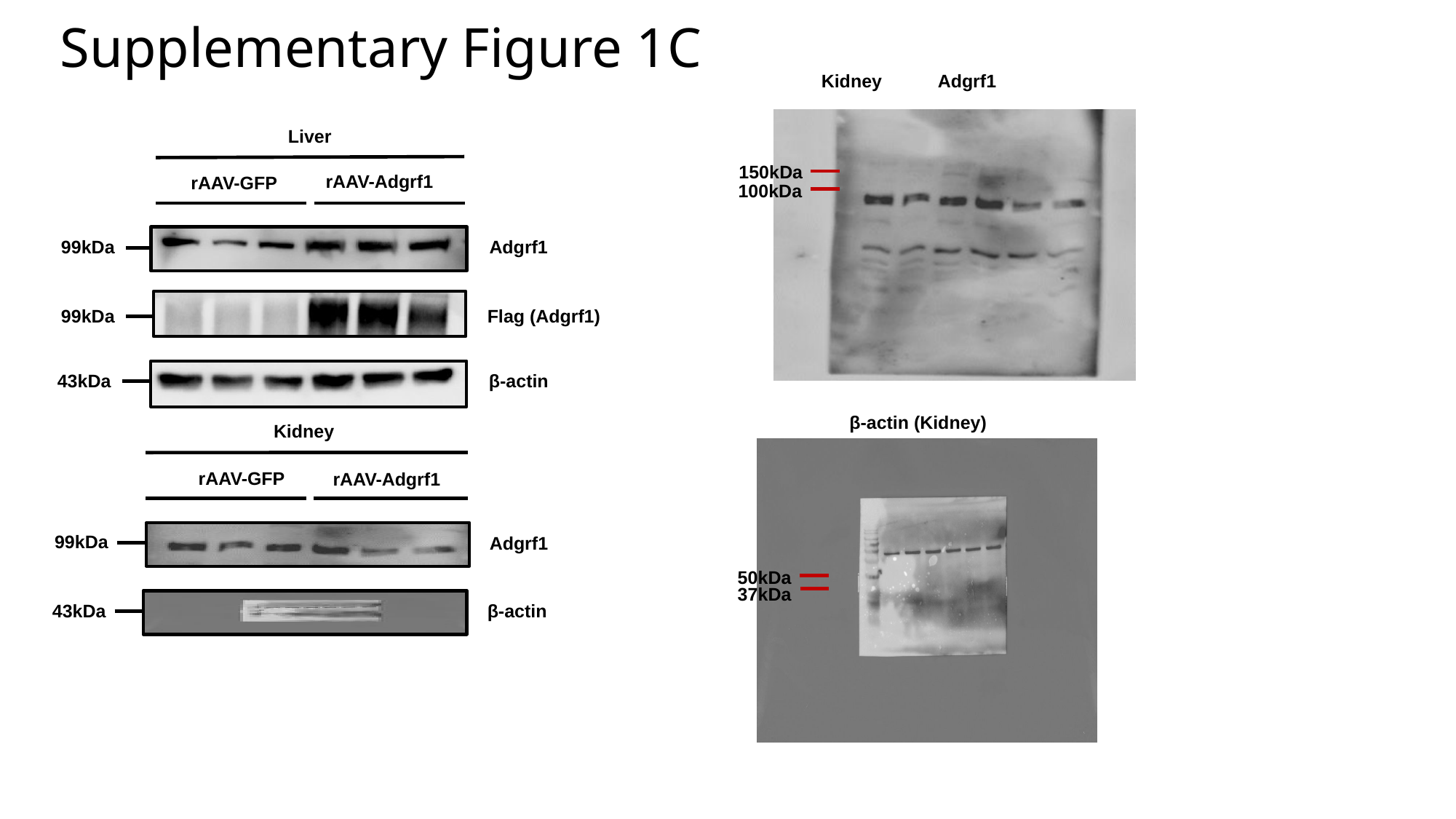

# Supplementary Figure 1C
Kidney
Adgrf1
150kDa
100kDa
Liver
rAAV-Adgrf1
rAAV-GFP
99kDa
Adgrf1
99kDa
Flag (Adgrf1)
43kDa
β-actin
Kidney
rAAV-GFP
rAAV-Adgrf1
99kDa
Adgrf1
43kDa
β-actin
β-actin (Kidney)
50kDa
37kDa
