# Supplementary material for: Amelioration of non-alcoholic fatty liver disease by targeting adhesion G protein-coupled receptor F1 (Adgrf1)
Source: eLife. 2023 Aug 15;12:e85131. doi: 10.7554/eLife.85131 (PMC10427146; doi:10.7554/eLife.85131)
Supplement: Figure 3—figure supplement 1—source data 1. [file elife-85131-fig3-figsupp1-data1.zip › Figure 3-figure supplement 1-source data 1/Supplementary Figure 3-Source 2.pptx]

## Slide 1
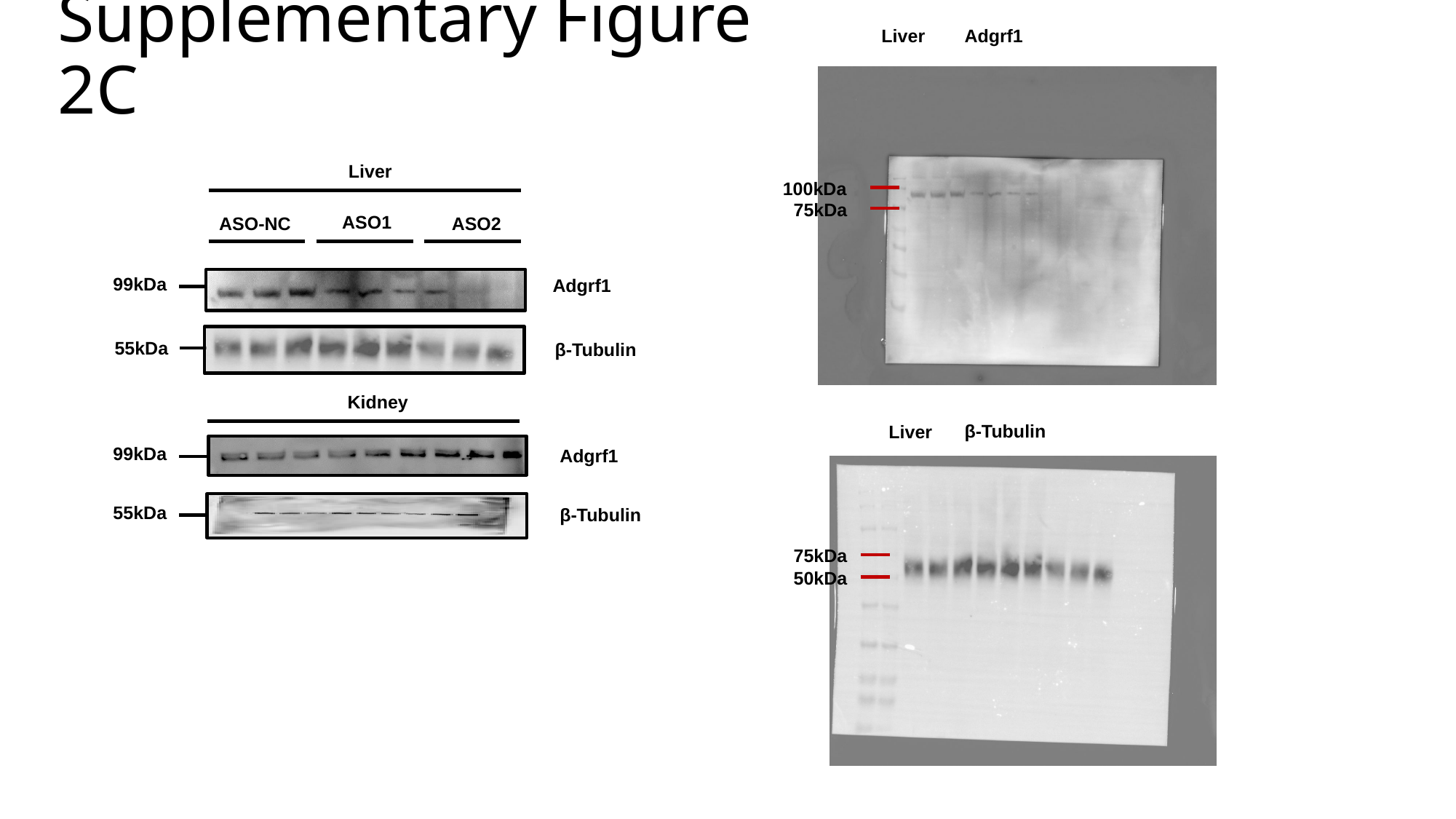

# Supplementary Figure 2C
Adgrf1
Liver
100kDa
75kDa
Liver
ASO1
ASO-NC
ASO2
99kDa
Adgrf1
55kDa
β-Tubulin
Kidney
99kDa
Adgrf1
55kDa
β-Tubulin
β-Tubulin
Liver
75kDa
50kDa

## Slide 2
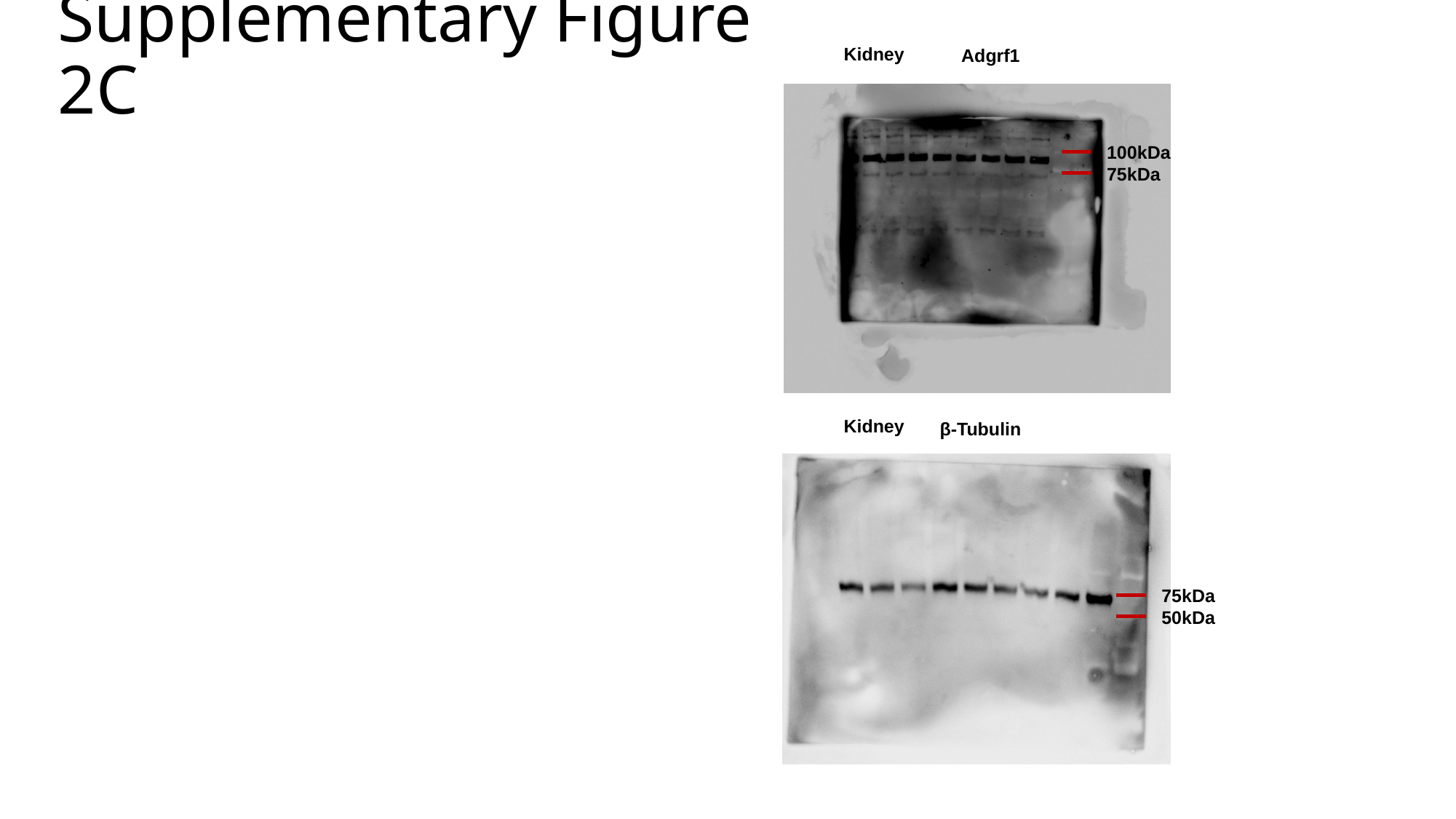

# Supplementary Figure 2C
Kidney
Adgrf1
100kDa
75kDa
Kidney
β-Tubulin
75kDa
50kDa
