# Supplementary material for: Amelioration of non-alcoholic fatty liver disease by targeting adhesion G protein-coupled receptor F1 (Adgrf1)
Source: eLife. 2023 Aug 15;12:e85131. doi: 10.7554/eLife.85131 (PMC10427146; doi:10.7554/eLife.85131)
Supplement: Figure 7—source data 1. [file elife-85131-fig7-data1.zip › Figure 7-source data 1/Figure 7-Source 2.pptx]

## Slide 1
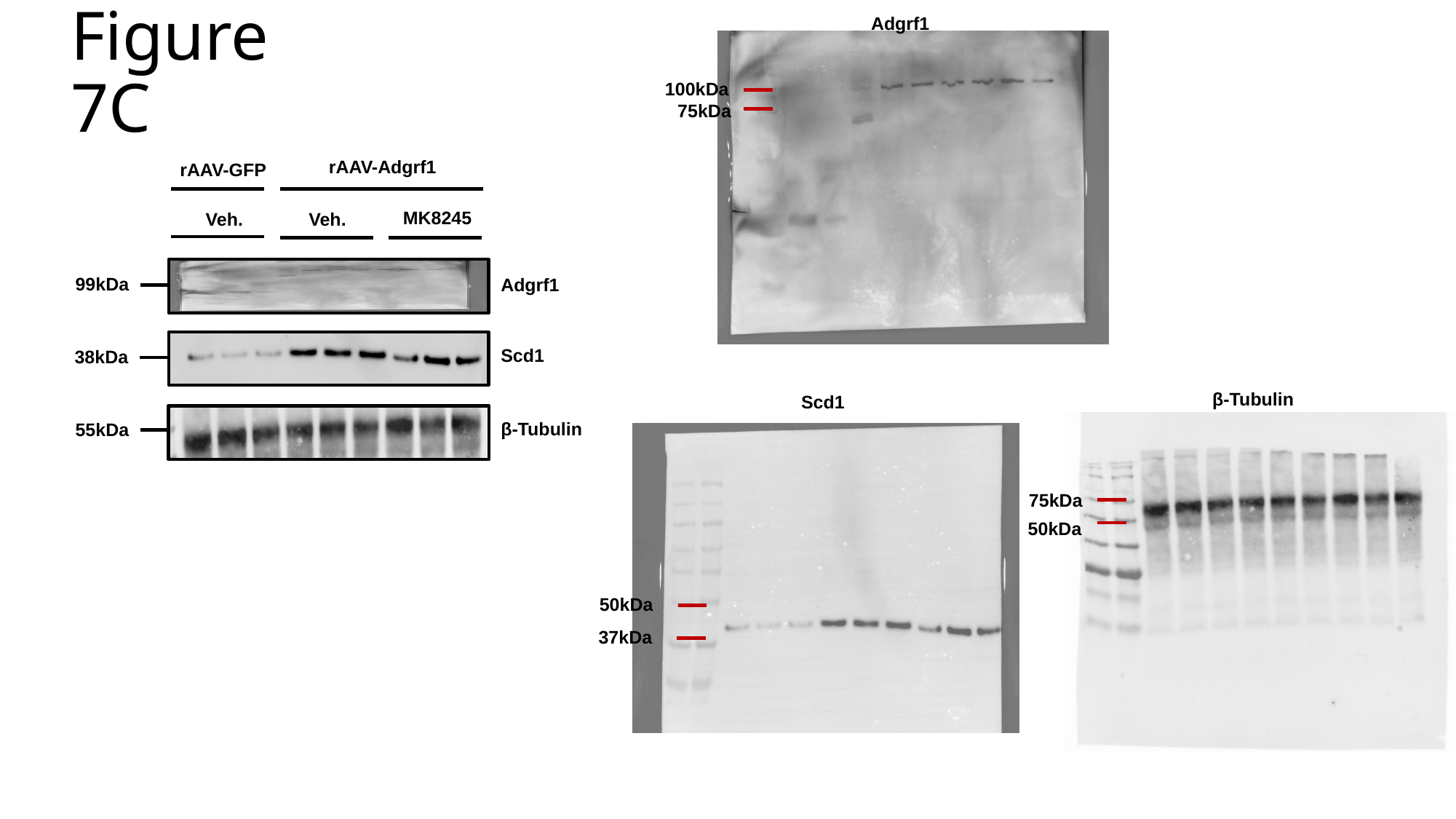

Adgrf1
100kDa
75kDa
# Figure 7C
rAAV-Adgrf1
rAAV-GFP
MK8245
Veh.
Veh.
99kDa
Adgrf1
38kDa
55kDa
Scd1
β-Tubulin
β-Tubulin
75kDa
50kDa
Scd1
50kDa
37kDa
